# Alactic Base Excess as a Predictor of Mortality in Trauma ICU Patients: Retrospective Study and Evaluation of Integration into Trauma Scoring Systems

**DOI:** 10.3390/life16101701

**Published:** 2026-10-10

**Authors:** Felix Julian Schöllhorn, Leonard Azamfirei, Oana Elena Branea

**Affiliations:** 1 Faculty of Medicine, George Emil Palade University of Medicine, Pharmacy, Science and Technology of Târgu Mureș, 540142 Târgu Mureș, Romania; 2 Department of Anaesthesiology and Intensive Care, George Emil Palade University of Medicine, Pharmacy, Science and Technology of Târgu Mureș, 540142 Târgu Mureș, Romania; leonard.azamfirei@umfst.ro (L.A.); oana.branea@umfst.ro (O.E.B.)

**Keywords:** alactic base excess, risk stratification, mortality prediction, intensive care, metabolic acidosis

## Abstract

Risk stratification in severe trauma remains a major clinical challenge: standard trauma scores (Injury Severity Score, ISS; Trauma and Injury Severity Score, TRISS) rely on anatomical and physiological data but lack metabolic components, potentially underestimating risk in haemodynamically compensated patients with underlying derangements. Alactic base excess (ABE) isolates the non-lactic fraction of metabolic acidosis by correcting base excess for lactate. We conducted a retrospective cohort study using the MIMIC-IV v3.1 database, including adult trauma patients with an ISS ≥ 16 and an arterial blood gas measurement within two hours of admission (*n* = 134), to assess ABE’s independent and incremental prognostic value for in-hospital and 30-day mortality, handling missing covariates by multiple imputation (*m* = 20). ABE was independently associated with in-hospital mortality after adjustment for age, New ISS (NISS), and mechanism of injury (odds ratio 0.84; 95% CI 0.745–0.946; *p* = 0.004; AUC 0.643), but its prognostic content overlapped substantially with lactate, and its incremental value beyond a clinical base model and beyond TRISS was small and statistically fragile; decision curve analysis showed no consistent net benefit from adding ABE to TRISS. The findings were consistent for 30-day mortality and stronger in a 60 min sensitivity cohort (*n* = 54; AUC 0.803), though this small subgroup may reflect selection effects. ABE should not be regarded as evidence of a novel physiological pathway in this trauma cohort; its practical contribution, if any, lies in immediate availability from a routine blood gas before ISS or TRISS can typically be computed. These findings are hypothesis-generating, given the single-centre design and absence of external validation, and require prospective, multicentre confirmation before clinical application.

## 1. Introduction

Trauma is amongst the leading causes of avoidable death worldwide, responsible for approximately 4.4 million deaths—or roughly 8% of global mortality—and the leading cause of death in individuals aged 5–44 years [1].

Severely injured patients pose major challenges in the ICU, with rapidly evolving pathophysiological mechanisms making early and accurate risk stratification essential. Acid–base disturbances are amongst the most common and prognostically important problems observed in this patient group. Metabolic acidosis, driven by tissue hypoperfusion, is of particular clinical significance: it impairs myocardial contractility, reduces vasopressor response, and exacerbates coagulopathy [2].

Serum lactate and base excess (BE) are the two most widely used biochemical markers of metabolic derangement in major trauma. Both are routinely obtained from the first arterial blood gas (ABG) and have been independently validated as predictors of mortality, blood transfusion requirement, and organ failure in trauma and general care settings [3,4,5]. However, each provides an incomplete picture: lactate reflects the hypoxic component of acidosis but is confounded by adrenergic stimulation and impaired hepatic clearance. BE, on the other hand, integrates all metabolic acid–base contributions but cannot identify which process is responsible [6].

Alactic base excess (ABE), introduced by Gattinoni et al. in 2019, aims to address this limitation by correcting BE for the quantifiable contribution of lactate [7]:

ABE (mmol/L) = Standard Base Excess (BE) + Lactate(1)

Adding lactate to standard BE isolates the non-lactic component of metabolic acidosis, allowing identification of processes that might affect acid–base balance, such as hyperchloraemia from crystalloid resuscitation, unmeasured anion accumulation, and early renal bicarbonate loss. A persistently negative ABE signals metabolic derangements beyond haemorrhagic shock and may carry independent prognostic significance [7].

ABE predicts mortality in sepsis [8,9], mixed ICU shock [10], and acute myocardial infarction [11]. To date, however, no study has evaluated ABE in a specifically defined severe trauma cohort, despite substantially different acid–base pathophysiology. Current trauma scoring systems, such as the Injury Severity Score (ISS), New ISS (NISS), Revised Trauma Score (RTS), and Trauma and Injury Severity Score (TRISS), contain no metabolic components, representing a recognised gap in early risk assessment [12,13].

The goals of this study were to: (1) determine whether ABE at admission is an independent predictor of in-hospital (primary outcome) and 30-day mortality (secondary outcome) in severely injured trauma ICU patients, and compare its discriminatory performance against established trauma scores; and (2) assess whether ABE provides incremental prognostic value beyond a clinical base model. The main aim is biomarker evaluation; multivariable regression serves as the analytical framework. As a prediction model development analysis reported in accordance with TRIPOD+AI, no external validation cohort was available.

## 2. Materials and Methods

### 2.1. Study Design and Setting

This retrospective, observational cohort study used the Medical Information Mart for Intensive Care IV (MIMIC-IV) v3.1 database, which contains de-identified clinical data from adult ICU admissions at the Beth Israel Deaconess Medical Center (BIDMC), a tertiary academic trauma centre in Boston, MA, USA, spanning 2008–2022 [14]. The observational aspects of this study adhere to the STROBE reporting guidelines for observational research (File S3) [15]. Prediction model development is reported in accordance with the TRIPOD+AI statement for transparent reporting of multivariable prediction models (File S4) [16].

### 2.2. Ethical Approval

MIMIC-IV data collection was approved by the Institutional Review Board (IRB) of the BIDMC and MIT (Protocol No. 2001-P-001699). All records are fully de-identified prior to release. PhysioNet access requires credentialed registration and CITI training. Local ethics review at the George Emil Palade University of Medicine, Pharmacy, Science and Technology of Târgu Mureș was waived on the basis of the retrospective, fully de-identified nature of the data. The study was conducted in accordance with the principles of the Declaration of Helsinki [17].

### 2.3. Participants

Adult patients were included if they: (1) were admitted to an ICU during the index hospitalisation; (2) had an ABG and serum lactate measurement within two hours of hospital admission; (3) had at least one trauma-related ICD-10 diagnosis code (S00–T88); and (4) had an ISS ≥ 16. Patients with pre-existing conditions significantly affecting acid–base homeostasis were excluded (full ICD-10-coded list: Table S1). Table S1 categorises each exclusion into a three-tier hierarchy: Tier 1 conditions have a direct, well-established effect on lactate or base excess (e.g., diabetic ketoacidosis, hepatic failure, septic shock); Tier 2 conditions have a plausible but indirect or variable effect, excluded conservatively (e.g., chronic hepatitis, COPD); and Tier 3 conditions have no established direct effect but were excluded to limit physiological heterogeneity (e.g., SIADH, hypernatraemia). This hierarchy was applied to minimise misclassification risk at the expense of some generalisability and cohort size. These inclusion criteria restrict the study population to severely injured trauma patients who survived to, and were clinically selected for, early arterial blood gas sampling. Therefore, the findings should be interpreted as applying to this population specifically rather than to all severe trauma ICU admissions. Patients with missing ABE components or primary outcome data were also excluded.

### 2.4. Data Processing

Demographic, laboratory, vital sign and outcome data were extracted from MIMIC-IV v3.1 using structured SQL queries via Google BigQuery, linking relational tables by hospital admission identifier (hadm_id). Base excess and lactate were extracted from the same arterial blood gas record, ensuring temporal alignment between the two components of ABE. Pre-admission (negative-time) draws were included to capture patients whose first blood gas was obtained during pre-hospital or trauma bay resuscitation shortly before formal admission timestamps were recorded. Where multiple qualifying measurements existed within the window, the earliest was retained. Admission time was defined as the hospital admission timestamp recorded in MIMIC-IV; for patients arriving via the emergency department, this timestamp can follow actual arrival, so the true interval from patient arrival to blood gas sampling may, in some cases, have exceeded the nominal window. Prior to analysis, lactate values were verified against raw database exports to detect locale-specific date-format artefacts (e.g., numeric values auto-converted to date strings in locale-Excel environments). Similarly, values inconsistent with physiologically plausible lactate concentrations were flagged as artefacts. Affected records were excluded from the 60 min sensitivity cohort. The identical plausibility screen was also applied to the primary 2 h cohort; no records were affected, confirming that this artefact was specific to the Excel-based export format of the 60 min sensitivity file and did not affect the primary CSV-based extraction. Extraction queries are provided in the Supplementary Materials (File S2). No study protocol was prospectively prepared, and the study was not pre-registered.

Generative artificial intelligence (Claude Sonnet 4.6, Anthropic, San Francisco, CA, USA) was used to assist with drafting SQL extraction queries, R code generation, and preliminary bias assessment during data processing and analysis. All AI-assisted outputs were reviewed and validated by the authors, who take full responsibility for the content and accuracy of the analysis.

### 2.5. Variables and ABE Calculation

ABE was calculated as the sum of BE and lactate as per Equation (1) [7]. Mechanism of injury was classified as blunt, penetrating or other/unknown based on the primary trauma ICD-10 diagnosis code.

The ISS and NISS were calculated via the ICDPIC-R package (v4.0) in R [18,19]. The RTS was derived from admission GCS, SBP, and respiratory rate. The TRISS probability of survival was calculated using the Boyd et al. (1987) coefficients [20], with the GCS values derived from multiply imputed datasets for participants with missing admission data.

### 2.6. Outcomes

The primary outcome was in-hospital mortality, determined from discharge disposition data; 30-day mortality was the secondary outcome, derived from the MIMIC-IV *dod* variable, which combines in-hospital death records with post-discharge mortality data from the Massachusetts state vital registry data for out-of-hospital deaths, flagged when death occurred within 30 days of admission.

### 2.7. Statistical Analysis

Continuous variables are presented as median [IQR]; categorical variables are presented as absolute frequencies and percentages. Comparisons between survivors and non-survivors used the Mann–Whitney U test and chi-squared or Fisher’s exact test, as appropriate.

Discrimination was assessed by ROC curve analysis with 95% CIs by DeLong’s method [21]. Standardised mean differences were calculated for key metabolic markers. The Youden index identified the optimal ABE threshold; sensitivity, specificity, PPV, and NPV were reported alongside. Pairwise AUC comparisons used DeLong’s test.

No formal sample size calculation was performed. All eligible patients within MIMIC-IV v3.1 were included (*n* = 134; 39 primary outcome events). The events-per-variable ratio was 7.8 (five parameters: ABE, age, NISS, and two mechanism dummy variables) at the lower boundary of conventional recommendations for logistic regression stability [22].

Missing covariate data were handled by multiple imputation by chained equations (MICE; 20 datasets, predictive mean matching), pooled by Rubin’s rules [23]. The GCS was missing in 47% of participants (predominantly intubation-related) and SBP in 10.4%. All primary predictors and outcome variables were complete.

All continuous predictors were entered without transformation; mechanism of injury was entered as a three-level categorical variable (blunt as reference). Predictor sets were specified a priori [24]. No stepwise selection was performed. Two prespecified sensitivity analyses assessed consistency.

Univariable logistic regression was performed before constructing three multivariable logistic regression models. Model 1 included ABE, age, ISS, and mechanism of injury. Model 2 substituted NISS for ISS and, given the recognised superiority of NISS in capturing multiple severe injuries, was designated the primary model [25,26]. Model 3 further built on Model 2 by additionally incorporating serum lactate. Given the Hosmer–Lemeshow test’s limited statistical power at small sample sizes [27], model calibration was additionally assessed by the Brier score, CITL, and calibration slope.

Model calibration was additionally assessed by the Brier score, calibration-in-the-large (CITL), and calibration slope with 95% bootstrap CI (500 iterations). A LOESS calibration plot is provided in the Supplementary Materials (Figure S1). Bootstrap optimism correction (500 iterations, Harrell’s method) was applied to the primary model AUC to account for overfitting in the development dataset.

Incremental value of ABE beyond the base model (age + NISS + mechanism) was assessed by the likelihood ratio test (LRT), the net reclassification index (NRI), and integrated discrimination improvement (IDI) (500-iteration bootstrap, 95% CIs).

Decision curve analysis was performed for the base model and TRISS, with and without ABE, to assess net clinical benefit across the plausible 10–35% threshold range [28].

Integration of ABE into TRISS was assessed by adding ABE to a logistic model incorporating TRISS probability of survival, calculated using Boyd et al. (1987) coefficients [20]. Incremental improvement was assessed by LRT, NRI and IDI [29].

Five sensitivity analyses were performed: one restricting ABE sampling to within 60 min of admission, one excluding patients with a concurrent sepsis diagnosis (ICD-10: A40/41, R6520/R6521), a third restricting the TRISS integration analysis to patients with observed, non-imputed GCS, given that GCS absence was concentrated in intubated patients and was not plausibly missing completely at random, a fourth excluding patients with alcohol-related diagnosis codes (ICD-10: F10.0, F10.1, T51.x), and a fifth adjusting the primary model for admission creatinine. Given the smaller sample size and sparse non-blunt-mechanism subgroup in the 60 min cohort, Firth’s penalised logistic regression was used for this sensitivity analysis to reduce small-sample bias and separation risk. The primary model, with a substantially larger sample, did not require this adjustment.

A two-tailed *p* < 0.05 was considered statistically significant throughout. All analyses were performed in R (v4.5.1; key packages: mice, pROC, logistf, gtsummary, ResourceSelection, readxl; full script in File S1).

## 3. Results

### 3.1. Cohort Characteristics

Of 17,599 adult ICU admissions with a trauma-related ICD-10 diagnosis, 15,987 (13,788 unique patients) lacked a qualifying arterial blood gas measurement within the inclusion window and were therefore not eligible for this study. This excluded population was older (median age 64 vs. 47 years) and comprised fewer men (56.5% vs. 75.4%) than the final cohort, with lower in-hospital mortality (11.9% vs. 29.1%; Table S2). This suggests that the excluded population was not predominantly composed of the highest-acuity patients who died before an early blood gas could be obtained but rather included a substantial portion of lower-acuity or clinically stable trauma admissions for whom an arterial blood gas was not indicated within the sampling window.

Of 134 patients meeting the inclusion criteria (Figure S2), 75.4% were male, with a median age of 47 years (IQR 29–64). Blunt trauma predominated (82.1%), mainly composed of motor vehicle collisions (*n* = 50, 37.3%) and falls (*n* = 44, 32.8%). The median ISS was 25 (IQR 17–32), and the median NISS was 34 (IQR 25–41). In-hospital mortality was 29.1% (*n* = 39), and 30-day mortality was 31.3% (*n* = 42). Baseline characteristics are presented in Table 1.

### 3.2. ABE Distribution, Threshold Analysis and Univariable Performance

ABE ranged from −14.6 to +6.7 mmol/L across the cohort (median −2.2 mmol/L, IQR −5.0 to +0.3), with a left-skewed distribution consistent with a predominance of metabolic acidosis. Non-survivors had significantly lower ABE than survivors (median −3.5 [IQR −6.1 to −1.5] vs. −1.2 [IQR −4.4 to +0.5] mmol/L; *p* = 0.010). The standardised mean difference for ABE (0.553) was smaller than for lactate (1.380) and BE (1.027), indicating that ABE carries a weaker univariable signal in this population.

Using the Youden index, the optimal ABE threshold for in-hospital mortality prediction was −1.45 mmol/L (sensitivity 76.9% [95% CI 64.1–89.7%], specificity 52.6% [95% CI 42.1–63.2%], PPV 40.0% [95% CI 33.8–47.1%], NPV 84.7% [95% CI 77.2–92.5%]), consistent for 30-day mortality (NPV 81.4%). This threshold was derived and evaluated on the same data and should be considered exploratory pending external validation.

Univariable analysis confirmed ABE as a significant predictor of in-hospital mortality (OR 0.870 per mmol/L, 95% CI 0.787–0.962, *p* = 0.007).

### 3.3. Multivariable Analysis

In the primary multivariable model (ABE + age + NISS + mechanism), ABE was independently associated with in-hospital mortality (OR 0.840 per mmol/L, 95% CI 0.745–0.946; *p* = 0.004; Table 2). The results were consistent for Model 1 (OR 0.842, *p* = 0.004). When lactate was added as an additional covariate (Model 3), ABE was no longer independently associated with either outcome (OR 0.982, *p* = 0.812), while lactate remained strongly significant (OR 1.631 per mmol/L, *p* < 0.001), indicating that the prognostic signal of ABE overlaps substantially with its lactate component. No formal mediation analysis was performed, and this overlap follows structurally from ABE’s mathematical derivation (ABE = BE + lactate) rather than from a causal mediating pathway. All findings were consistent for 30-day mortality (primary model: OR 0.841, 95% CI 0.747–0.946; *p* = 0.004).

All three models demonstrated adequate calibration (Hosmer–Lemeshow: Model 1 *p* = 0.467; Model 2 *p* = 0.843; Model 3 *p* = 0.901; complete case, *n* = 134). For the primary model, the Brier score was 0.145 (scaled Brier 0.295), calibration-in-the-large was 0.000 (95% CI −0.455 to 0.437), and calibration slope was 1.000 (95% CI 0.650 to 1.413), confirming no systematic over- or under-prediction. It should be noted that evaluating this formula at central (e.g., median) predictor values yields a lower probability than the cohort’s observed mortality rate. This reflects Jensen’s inequality for this nonlinear model given the right-skewed distribution of NISS and age, rather than a miscalibration, and is consistent with the near-zero CITL reported here, which reflects the average predicted probability across all patients rather than the probability at central predictor values. A LOESS-smoothed calibration plot is provided in the Supplementary Materials (Figure S1).

The predicted probability of death was calculated as:

P(death) = 1/(1 + e^−^*^L^*) (2)

where *L* = −7.1885 − 0.1747(*ABE*) + 0.0517(*Age*) + 0.0792(*NISS*) + 1.8620(*Pen*) + 1.7002(*Other*), with:

- *ABE* in mmol/L;
- *Age* in years;
- *NISS* in points;
- *Pen* = 1 if mechanism is penetrating, 0 if not;
- *Other* = 1 if mechanism is Other/Unknown, 0 if not.

This equation is reported for transparency and reproducibility only and should not be applied to individual patients or used for clinical decision-making without prospective external validation.

### 3.4. Discriminatory Performance and Incremental Value

**Figure 1:**
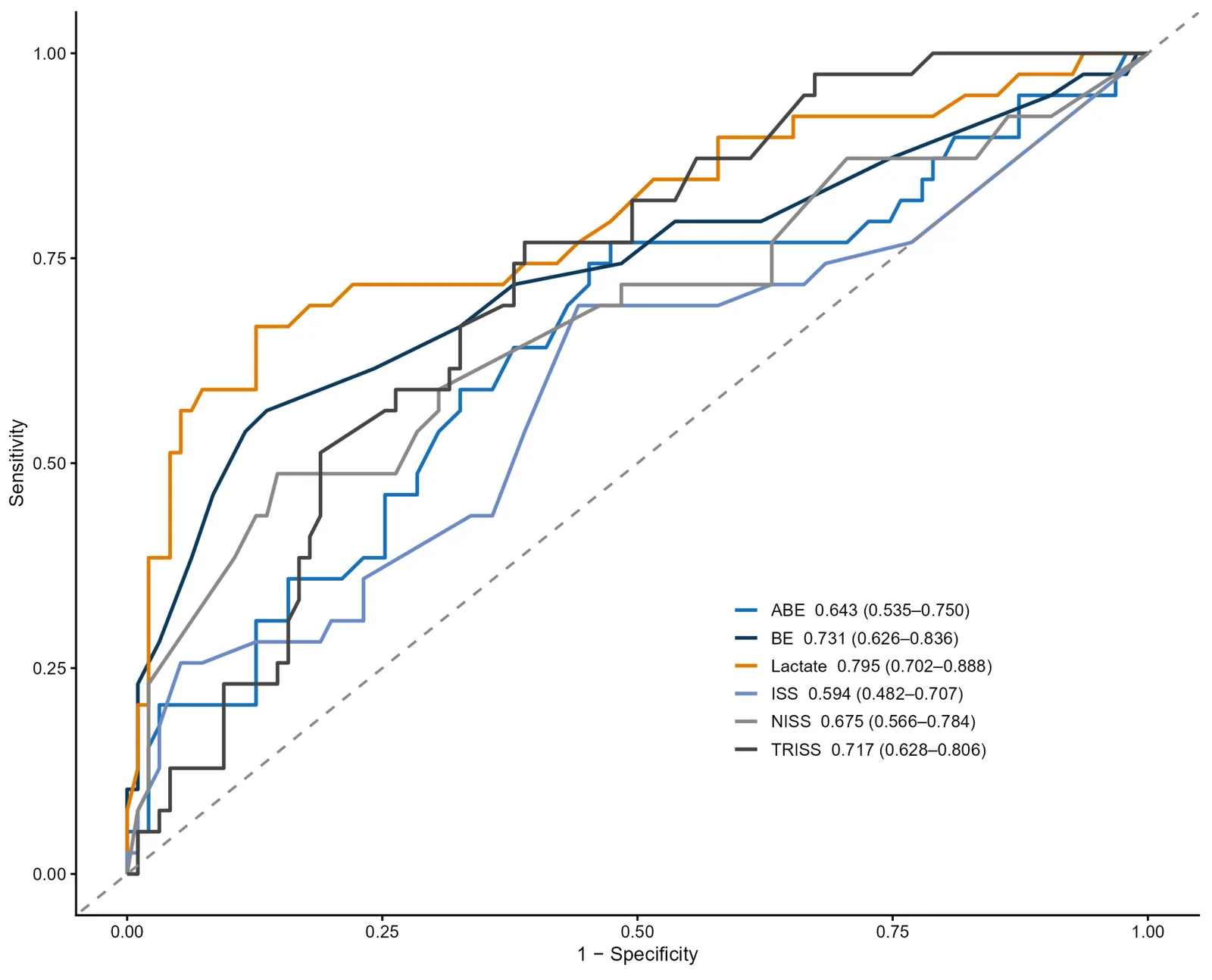
ROC curves for individual markers against in-hospital mortality.

ABE achieved an AUC of 0.643 (95% CI 0.535–0.750) for in-hospital mortality. This showed no statistically detectable difference from the NISS (0.675; DeLong *p* = 0.655) and a numerically higher, but not statistically significant, AUC than ISS (0.594; DeLong *p* = 0.493); both comparisons were underpowered at *n* = 134 to exclude differences of the observed magnitude. ABE was significantly inferior to lactate (0.795; *p* = 0.001) and BE (0.731; *p* < 0.001). The full results are shown in Figure 1.

Adding ABE to the base clinical model improved the AUC from 0.799 to 0.826 (*p* = 0.360). LRT confirmed significant incremental model fit improvement (LRT *p* = 0.002), with an NRI of 0.681 (95% CI 0.346–1.015; *p* = 0.002) and an IDI of 0.057 (*p* = 0.020). Secondary outcome analyses were consistent (30-day: LRT *p* = 0.002; NRI 0.590, *p* = 0.002; IDI 0.054, *p* = 0.024). The full metrics are shown in Table 3.

Decision curve analysis (Figure S4) assessed whether these incremental discrimination and reclassification metrics translate into higher net clinical benefit across plausible risk thresholds. For the base model, adding ABE produced a small and largely consistent net benefit gain across the 10–35% threshold range (net benefit 0.141–0.230 for base + ABE vs. 0.114–0.221 for base model alone), with the clearest difference at the highest threshold examined. For TRISS, adding ABE did not consistently improve net benefit: at lower-to-moderate risk thresholds (10–20%), TRISS+ABE showed lower net benefit than TRISS alone (e.g., at 20%: 0.132 vs. 0.149), with an advantage only apparent at higher thresholds (29–35%; e.g., at 29%: 0.103 vs. 0.083). These findings do not provide consistent evidence of improved clinical decision-making from integrating ABE into either model.

Bootstrap optimism correction (500 iterations, Harrell’s method) yielded adjusted AUCs of 0.793 for the primary model (apparent AUC 0.826; mean optimism 0.033) and 0.775 for the base model (apparent AUC 0.799; mean optimism 0.024), with an optimism-corrected ΔAUC attributable to ABE of 0.018.

### 3.5. TRISS Integration

TRISS probability of survival was calculated for all 134 patients using the Boyd et al. (1987) coefficients [20] (AUC 0.717, 95% CI 0.628–0.806). Adding ABE increased the AUC to 0.727 (DeLong *p* = 0.727). The likelihood ratio testing confirmed significant incremental improvement (LRT *p* = 0.029); IDI was 0.039 (*p* = 0.040), and the NRI was 0.328 (*p* = 0.072). The results were consistent for 30-day mortality (LRT *p* = 0.041; NRI 0.291, *p* = 0.12; IDI 0.033, *p* = 0.036). The primary outcome findings are summarised in Table 4.

In a sensitivity analysis restricted to the 71 patients (53.0%) with observed, non-imputed GCS, TRISS alone reached a substantially higher AUC (0.877, 95% CI 0.794–0.960) than in the full, imputed-GCS cohort. Adding ABE did not improve discrimination in this subset (AUC 0.861, 95% CI 0.769–0.952; DeLong *p* = 0.309; LRT *p* = 0.263; NRI 0.165, *p* = 0.516; IDI 0.016, *p* = 0.312).

### 3.6. Sensitivity Analysis

Of 61 patients (45.5%) with ABG values obtained within 60 min of admission, 54 had valid ABE after excluding seven date-format artefacts. In this early-measurement subset, ABE performance improved substantially: AUC 0.803 (95% CI 0.686–0.920), OR 0.610 per mmol/L (Firth penalised logistic regression; 95% CI 0.409–0.798; *p* < 0.001), and NPV 95.5% (95% CI 85.7–100.0%) at an optimal threshold of −2.85 mmol/L (Figure S3); this threshold was likewise derived and evaluated on the same data and should be regarded as descriptive rather than a validated clinical cut-off. The in-hospital mortality rate (29.5%) was consistent with the primary cohort, suggesting comparable baseline mortality risk.

A secondary sensitivity analysis excluding the eight patients with concurrent sepsis-related diagnoses (A40/A41, R6520, R6521) produced virtually identical results (*n* = 126; AUC 0.637, 95% CI 0.528–0.747; OR 0.844, 95% CI 0.749–0.952; *p* = 0.006; optimal threshold unchanged at −1.45 mmol/L), confirming that concurrent sepsis coding did not materially influence the primary findings.

Excluding the 20 patients (14.9%) with alcohol-related diagnosis codes produced a result consistent with the primary analysis (*n* = 114; AUC 0.687, 95% CI 0.582–0.792; OR 0.768, 95% CI 0.667–0.885; *p* < 0.001).

Adjusting the primary model for admission creatinine did not materially attenuate the ABE association (*n* = 127 complete case; OR 0.852, 95% CI 0.754–0.962; *p* = 0.010), while creatinine itself was not independently associated with mortality in this model (OR 1.481, 95% CI 0.497–4.413; *p* = 0.480).

## 4. Discussion

ABE at hospital admission was independently associated with in-hospital mortality in severely injured trauma ICU patients (age, NISS, and mechanism adjusted), with consistent findings for 30-day mortality. To our knowledge, this is the first ABE evaluation in a cohort specifically defined by severe traumatic injury (ISS ≥ 16).

These findings extend the existing ABE literature, currently dominated by sepsis [8,9,10], to the trauma setting. Performance was numerically higher in the early-ABG sensitivity cohort (AUC 0.803 vs. 0.643 in the primary cohort). This subgroup, however, is small (*n* = 54) and non-randomly selected, so the stronger performance may partly reflect selection or spectrum effects rather than purely reflecting an earlier measurement timepoint. For instance, patients sampled within 60 min may differ systematically in injury pattern or acuity. This finding should therefore be regarded as hypothesis-generating rather than establishing a superior early diagnostic threshold, consistent with survivorship-bias concerns raised in comparable trauma cohorts using delayed metabolic measurements [30].

The collinearity finding—ABE losing independence when lactate is added to the model—follows structurally from ABE’s mathematical derivation: when lactate is the primary driver of metabolic acidosis in haemorrhagic shock, BE and lactate necessarily co-vary, making them collinear by definition. ABE therefore primarily functions as a convenient composite marker rather than an independent signal of non-lactic acidosis in the acute trauma phase.

The ROC hierarchy (lactate outperforming ABE [AUC 0.795 vs. 0.643]) follows directly from this collinearity. ABE showed no statistically detectable difference from NISS (0.675; DeLong *p* = 0.655) and a numerically higher, but not statistically significant, AUC than ISS (0.594; DeLong *p* = 0.493). Both comparisons were underpowered at *n* = 134 to exclude differences of this magnitude, and neither can or should be read as evidence that ABE outperforms these anatomical scores. Performance was broadly comparable to that in mixed shock ICU populations (AUC 0.687 [10]), suggesting that the prognostic signal is preserved but not enhanced in trauma. These findings do not support using ABE in place of lactate or BE in early trauma care; rather, ABE’s potential role is as a convenient single-number composite when both components are already being measured.

The incremental value analysis suggests a modest and statistically fragile contribution from ABE. ABE did not significantly improve the AUC beyond the base model by DeLong’s test (ΔAUC 0.026, *p* = 0.360; optimism-corrected ΔAUC 0.018), though the LRT (which tests whether ABE improves model fit, a different question from DeLong’s test of discrimination) confirmed genuine improved model fit (*p* = 0.002), and the IDI (0.057, *p* = 0.020) demonstrated that predicted risks were better separated between survivors and non-survivors. The NRI was also significant (category-free NRI 0.681, *p* = 0.002); however, this category-free approach can reward reclassification that does not correspond to any change in clinical decision-making, and the real value of this magnitude in a cohort of 134 patients should be interpreted cautiously. Taken together, these metrics indicate a statistically detectable but small and fragile incremental effect. On their own, though, these findings do not establish that ABE meaningfully changes clinical decision-making, and any such claim would require external validation and formal decision-analytic evaluation. Decision curve analysis reinforced this cautious interpretation: net benefit gains from adding ABE to the base model were small across plausible thresholds, providing limited evidence of improved clinical decision-making (Figure S4).

While LRT confirmed incremental improvement in TRISS model fit (*p* = 0.029) and IDI demonstrated better probability separation (0.039; *p* = 0.040), NRI did not reach significance (0.328; *p* = 0.072), reflecting the limited statistical power in this cohort. Critically, ABE integration did not impair TRISS calibration (χ^2^ = 4.40, *p* = 0.819), preserving the score’s prognostic architecture [31]. These TRISS integration findings require external validation before clinical implementation. Decision curve analysis tempers this further: net benefit from adding ABE to TRISS was lower than TRISS alone at low-to-moderate risk thresholds and only modestly higher at higher thresholds (Figure S4), providing no consistent evidence that this integration improves clinical decision-making in its current form. Notably, TRISS’s own discrimination differed substantially between the imputed-GCS cohort (AUC 0.717) and the observed-GCS-only subset (AUC 0.877), illustrating that GCS imputation may materially affect TRISS-based comparisons and should be considered when interpreting the TRISS integration findings.

Timing is central to ABE’s practical rationale, though this rationale should be stated carefully. Our primary cohort defines early sampling as within two hours of admission, which is broader than the hyper-acute “golden hour” window in which the most time-critical trauma decisions are made. This means that lactate and base excess trends may already be partially established by that time. ABE’s comparative advantage is therefore not that it is uniquely instantaneous, but that it—unlike ISS or TRISS—requires no additional coding, specialist input or delay beyond the blood gas result itself, and so remains computable at time points when the anatomical scores are typically not yet available in routine clinical practice. This is a modest, practical advantage rather than evidence of a distinct golden-hour diagnostic capability, and the NPV-based rule-out utility reported here (84.7% at the optimal threshold, 95.5% in the 60 min cohort) should be treated as exploratory pending external validation.

### Strengths and Limitations

Strengths include MIMIC-IV’s publicly available data, complete primary variable availability, MICE for missing covariates, and four complementary incremental value metrics beyond AUC alone. The sensitivity analyses provide convergent validation.

Limitations include a single-centre cohort (*n* = 134) limiting subgroup analyses and generalisability. BIDMC’s specific resuscitation protocols may not represent district-level or resource-limited settings. Performance estimates derive from the development dataset, and without external validation, some optimism in discrimination and calibration cannot be excluded. GCS was missing in 47% of patients due to intubation. TRISS coefficients predate modern damage control resuscitation. Patients sampled within 60 min may represent a higher-acuity subgroup, partly explaining the stronger discriminatory performance in that cohort; because admission time reflects the MIMIC-IV hospital admission timestamp rather than emergency department arrival, the true time from arrival to sampling may exceed these nominal windows for some patients. Because eligibility required survival to, and clinical selection for, early arterial blood gas sampling, the study population is not representative of all severe trauma ICU admissions. Findings can therefore only be generalised to patients undergoing similarly early metabolic assessment. Acute alcohol intoxication and acute kidney injury were not applied as primary exclusion criteria, though both were addressed in sensitivity analyses: excluding alcohol-related diagnoses and adjusting for admission creatinine each left the ABE association materially unchanged (Section 3.6), suggesting neither is a major driver of the primary result. Acute kidney injury was deliberately retained as a direct consequence of haemorrhagic shock rather than treated as a pre-existing confounder. No correction for multiple comparisons was applied across the numerous secondary analyses, metrics, and sensitivity analyses reported, which should be considered when interpreting nominally significant results. No formal analysis of model fairness across sociodemographic subgroups was conducted. The sample size precluded stratified performance estimates, and the BIDMC derivation cohort may not represent demographic diversity across trauma populations. ABE should be interpreted as a marker of physiological derangement, not a therapeutic target. The full SQL extraction and R analysis code provided (Files S1 and S2) can be adapted to other sources, such as the eICU Collaborative Research Database or institutional trauma registries, to support external validation. Serial measurements (e.g., trends in ABE, lactate clearance, or repeated base excess over the first hours of resuscitation) may carry more prognostic information than a single admission value and represent a natural direction for future work.

## 5. Conclusions

ABE at hospital admission was independently associated with in-hospital mortality in severely injured trauma ICU patients, but its incremental value beyond established clinical markers and scoring systems was modest and statistically fragile. Its prognostic content overlapped substantially with lactate, on which it is mathematically based. These findings should not be read as evidence that ABE reflects a novel physiological process in this cohort. Instead, ABE’s practical contribution is more likely that of a convenient single-number composite, available immediately from a routine blood gas at timepoints when ISS and TRISS are typically not yet computable, as opposed to a superior standalone predictor. Given the single-centre design, modest sample size, and absence of external validation, these findings are hypothesis-generating rather than directly clinically actionable, and the thresholds as well as coefficients reported here should not be applied clinically before prospective, multicentre validation in larger and more diverse cohorts is undertaken.

## Figures and Tables

**Table 1:** Baseline characteristics stratified by in-hospital mortality outcome.

Variable	Overall (*n* = 134)	Survived (*n* = 95)	Died (*n* = 39)
Age, years—median [IQR]	47 [29–64]	43 [28–62]	57 [39–77] a
Male sex—*n* (%)	101 (75.4)	74 (77.9)	27 (69.2)
Blunt mechanism—*n* (%)	110 (82.1)	80 (84.2)	30 (76.9)
ISS—median [IQR]	25 [17–32]	22 [17–29]	26 [17–45]
NISS—median [IQR]	34 [25–41]	29 [22–41]	38 [26–50] b
ABE, mmol/L—median [IQR]	−2.2 [−5, 0.3]	−1.2 [−4.4, 0.5]	−3.5 [−6.1, −1.5] b
BE, mmol/L—median [IQR]	−5 [−10, −2]	−4 [−7, −1]	−11 [−14, −4] c
Lactate, mmol/L—median [IQR]	2.85 [1.80–5.10]	2.50 [1.6–3.6]	6.40 [2.7–8] c
GCS d—median [IQR]	6 [3–11]	9 [6–13]	3 [3–6] c
SBP, mmHg e—median [IQR]	123 [108–143]	128 [113–146]	109 [96–136] b
Hospital LOS, days—median [IQR]	10.5 [3.4–20.8]	14.3 [8.1–24.7]	1.3 [0.3–4.4] c

Values expressed as median [IQR] unless otherwise stated. a: *p* < 0.05; b: *p* < 0.01; c: *p* < 0.001 (Mann–Whitney U test for continuous, chi-square for categorical variables). ABE: alactic base excess; BE: base excess; GCS: Glasgow Coma Scale; ISS: Injury Severity Score; LOS: length of stay; NISS: New Injury Severity Score; SBP: systolic blood pressure. d: GCS missing in *n* = 63 (47%); e: SBP missing in *n* = 14 (10.4%). All other variables complete. *p*-values are presented for descriptive purposes only and should be interpreted as exploratory; between-group comparisons were not specified.

**Table 2:** Primary multivariable logistic regression model (ABE + age + NISS + mechanism) for in-hospital mortality.

Variable	OR	95% CI	*p*-Value
ABE (per 1 mmol/L increase)	0.840	0.745–0.946	0.004
Age (per year)	1.053	1.025–1.082	<0.001
NISS (per point)	1.082	1.042–1.124	<0.001
Penetrating mechanism (vs. blunt)	6.436	1.274–32.51	0.025
Other/unknown mechanism (vs. blunt)	5.475	1.098–27.31	0.038

Results based on 20 multiply imputed datasets pooled by Rubin’s rules. OR: odds ratio; CI: confidence interval; ABE: alactic base excess; NISS: New Injury Severity Score.

**Table 3:** Incremental value of ABE.

Metric	In-Hospital	30-Day
AUC—Base model (95% CI)	0.799 (0.713–0.886)	0.791 (0.706–0.875)
AUC—Base + ABE (95% CI)	0.826 (0.75–0.902)	0.82 (0.746–0.893)
ΔAUC	0.026	0.029
DeLong *p*	0.36	0.297
LRT χ^2^	9.254	9.389
LRT *p*	0.002	0.002
NRI	0.681	0.59
NRI 95% CI	0.346–1.015	0.23–0.927
NRI *p*	0.002	0.002
NRI+ (events)	0.333	0.286
NRI− (non-events)	0.347	0.304
IDI	0.057	0.054
IDI 95% CI	0.008–0.107	0.006–0.104
IDI *p*	0.02	0.024

NRI: net reclassification improvement; IDI: integrated discrimination improvement; 500-iteration bootstrap; *p*-values test H0 = 0. Base model: age + NISS + mechanism. Results from pooled multiple imputation (m = 20, Rubin’s rules). Bootstrap optimism-corrected AUC: primary model 0.793 (apparent 0.826, optimism 0.033); base model 0.775 (apparent 0.799, optimism 0.024); optimism-corrected ΔAUC 0.018.

**Table 4:** Discriminatory performance of TRISS alone versus TRISS augmented with ABE for in-hospital mortality.

Model	AUC (95% CI)	DeLong *p*	LRT *p*	NRI (95% CI; *p*)	IDI (95% CI; *p*)
TRISS alone	0.717 (0.628–0.806)	–	–	–	–
TRISS + ABE	0.727 (0.633–0.821)	0.727	0.029	0.328 (−0.022–0.735; *p* = 0.072)	0.039 (0.003–0.076; *p* = 0.04)

DeLong *p*-values are for comparison against the TRISS alone model. NRI and IDI from 500-iteration bootstrap; *p*-values test H0 = 0. Results from pooled multiple imputation (m = 20, Rubin’s rules). LRT: likelihood ratio test; NRI: net reclassification improvement; IDI: integrated discrimination improvement; TRISS: Trauma and Injury Severity Score; ABE: alactic base excess.

## Data Availability

Restrictions apply to the availability of these data. Data were obtained from PhysioNet [32] and are available at https://physionet.org/content/mimiciv/3.1/ (accessed on 7 October 2026) with the permission of the MIMIC-IV data use agreement, following completion of required CITI training and credentialed registration. The R analysis script and SQL extraction queries used to generate and analyse the dataset for this study are available in the Supplementary Materials (Files S1 and S2).

## Supplementary Materials

The following supporting information can be downloaded at: https://www.mdpi.com/article/10.3390/life16101701/s1. Table S1: Exclusion diagnoses with ICD-10-CM codes and rationale; Table S2: Characteristics of patients excluded for missing an early arterial blood gas; Figure S1: LOESS-smoothed calibration plot for the primary model; Figure S2: Participant flow diagram; Figure S3: Comparison of ABE discriminatory performance, primary versus 60-minute sensitivity cohort; Figure S4: Decision curve analysis for the base model and TRISS, with and without ABE; File S1: R analysis script; File S2: SQL extraction queries; File S3: STROBE checklist; File S4: TRIPOD+AI checklist.

## Abbreviations

The following abbreviations are used in this manuscript:

ABE	Alactic base excess
ABG	Arterial blood gas
AI	Artificial intelligence
AIS	Abbreviated Injury Scale
AUC	Area under the receiver operating characteristic curve
BE	Base excess
BIDMC	Beth Israel Deaconess Medical Center
CI	Confidence interval
CITI	Collaborative Institutional Training Initiative
CITL	Calibration-in-the-large
GCS	Glasgow Coma Scale
ICD-10	International Classification of Diseases, 10th Revision
ICDPIC-R	International Classification of Diseases Programs for Injury Categorization (R package)
IDI	Integrated discrimination improvement
IQR	Interquartile range
IRB	Institutional Review Board
ISS	Injury Severity Score
LOESS	Locally estimated scatterplot smoothing
LOS	Length of stay
LRT	Likelihood ratio test
MICE	Multiple imputation by chained equations
MIMIC-IV	Medical Information Mart for Intensive Care IV
MIT	Massachusetts Institute of Technology
NISS	New Injury Severity Score
NPV	Negative predictive value
OR	Odds ratio
PPV	Positive predictive value
ROC	Receiver operating characteristic
RTS	Revised Trauma Score
SBP	Systolic blood pressure
SQL	Structured Query Language
STROBE	STrengthening the Reporting of OBservational studies in Epidemiology
TRIPOD+AI	Transparent Reporting of a multivariable prediction model for Individual Prognosis Or Diagnosis + Artificial Intelligence
TRISS	Trauma and Injury Severity Score
