# Supplementary material for: Alactic Base Excess as a Predictor of Mortality in Trauma ICU Patients: Retrospective Study and Evaluation of Integration into Trauma Scoring Systems
Source: Life (Basel). 2026 Oct 10;16(10):1701. doi: 10.3390/life16101701 (PMC13706226; doi:10.3390/life16101701)
Supplement: Supplementary file 1 [file life-16-01701-s001.zip › FigureS1.pdf]

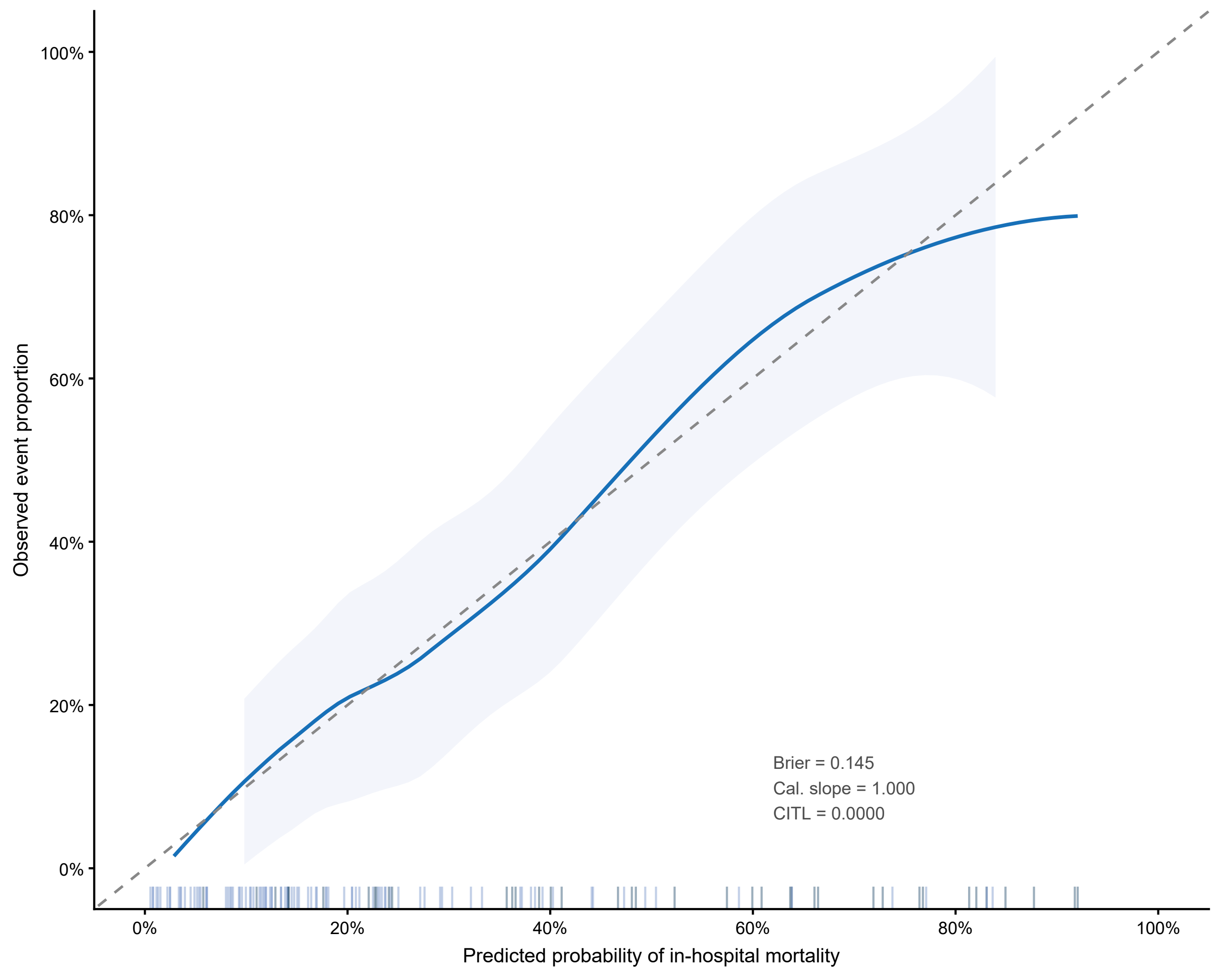

Observed event proportion
100%
80%
60%
40%
20%
0%
0%
20%
40%
60%
80%
100%
Predicted probability of in-hospital mortality
Brier = 0.145
Cal. slope = 1.000
CITL = 0.0000
