# Supplementary material for: Alactic Base Excess as a Predictor of Mortality in Trauma ICU Patients: Retrospective Study and Evaluation of Integration into Trauma Scoring Systems
Source: Life (Basel). 2026 Oct 10;16(10):1701. doi: 10.3390/life16101701 (PMC13706226; doi:10.3390/life16101701)
Supplement: Supplementary file 1 [file life-16-01701-s001.zip › FigureS2.pdf]

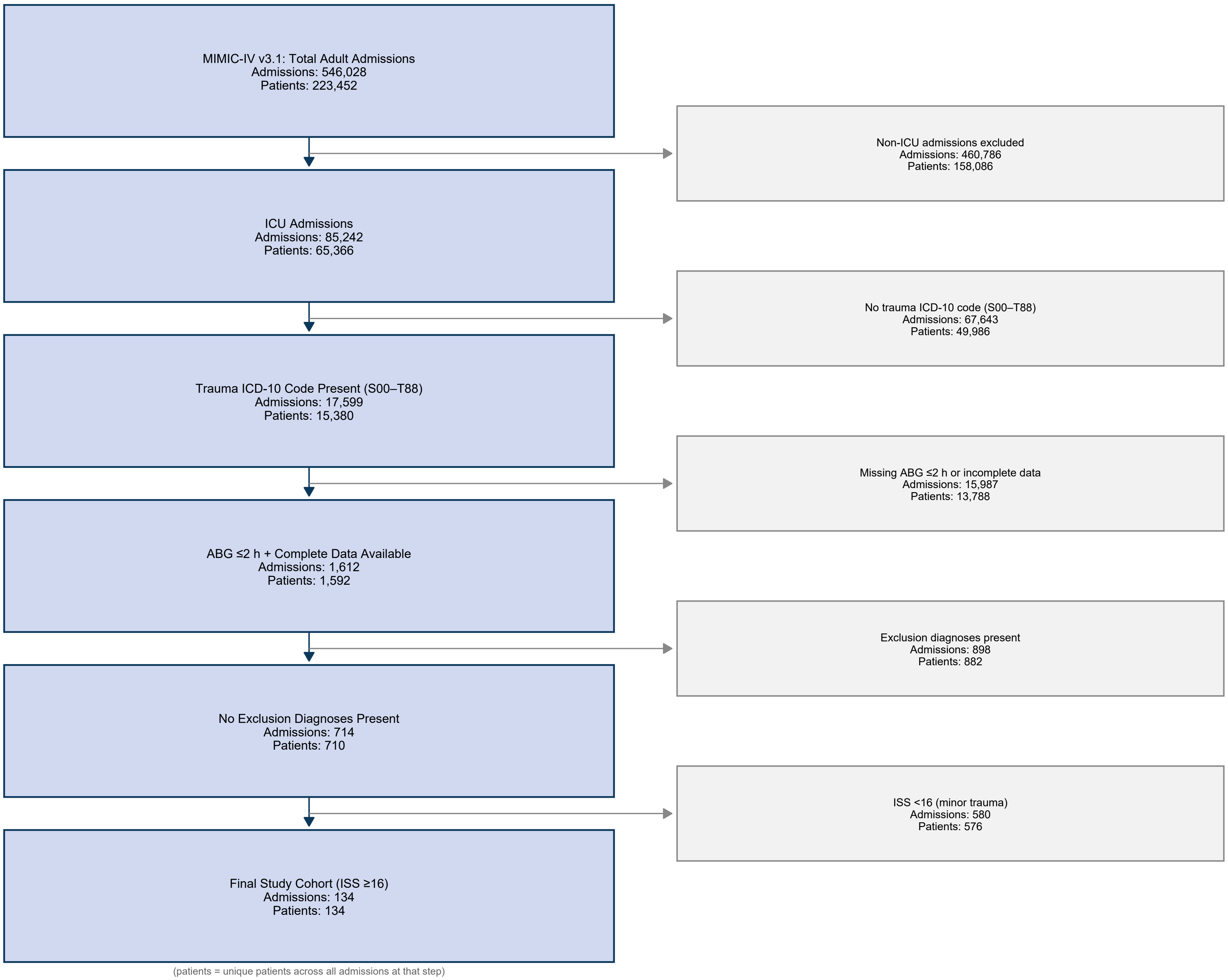
MIMIC-IV v3.1: Total Adult Admissions
Admissions: 546,028
Patients: 223,452
Non-ICU admissions excluded
Admissions: 460,786
Patients: 158,086
ICU Admissions
Admissions: 85,242
Patients: 65,366
No trauma ICD-10 code (S00–T88)
Admissions: 67,643
Patients: 49,986
Trauma ICD-10 Code Present (S00–T88)
Admissions: 17,599
Patients: 15,380
Missing ABG ≤2 h or incomplete data
Admissions: 15,987
Patients: 13,788
ABG ≤2 h + Complete Data Available
Admissions: 1,612
Patients: 1,592
Exclusion diagnoses present
Admissions: 898
Patients: 882
No Exclusion Diagnoses Present
Admissions: 714
Patients: 710
ISS <16 (minor trauma)
Admissions: 580
Patients: 576
Final Study Cohort (ISS ≥16)
Admissions: 134
Patients: 134
(patients = unique patients across all admissions at that step)
