# Supplementary material for: Alactic Base Excess as a Predictor of Mortality in Trauma ICU Patients: Retrospective Study and Evaluation of Integration into Trauma Scoring Systems
Source: Life (Basel). 2026 Oct 10;16(10):1701. doi: 10.3390/life16101701 (PMC13706226; doi:10.3390/life16101701)
Supplement: Supplementary file 1 [file life-16-01701-s001.zip › FigureS3.pdf]

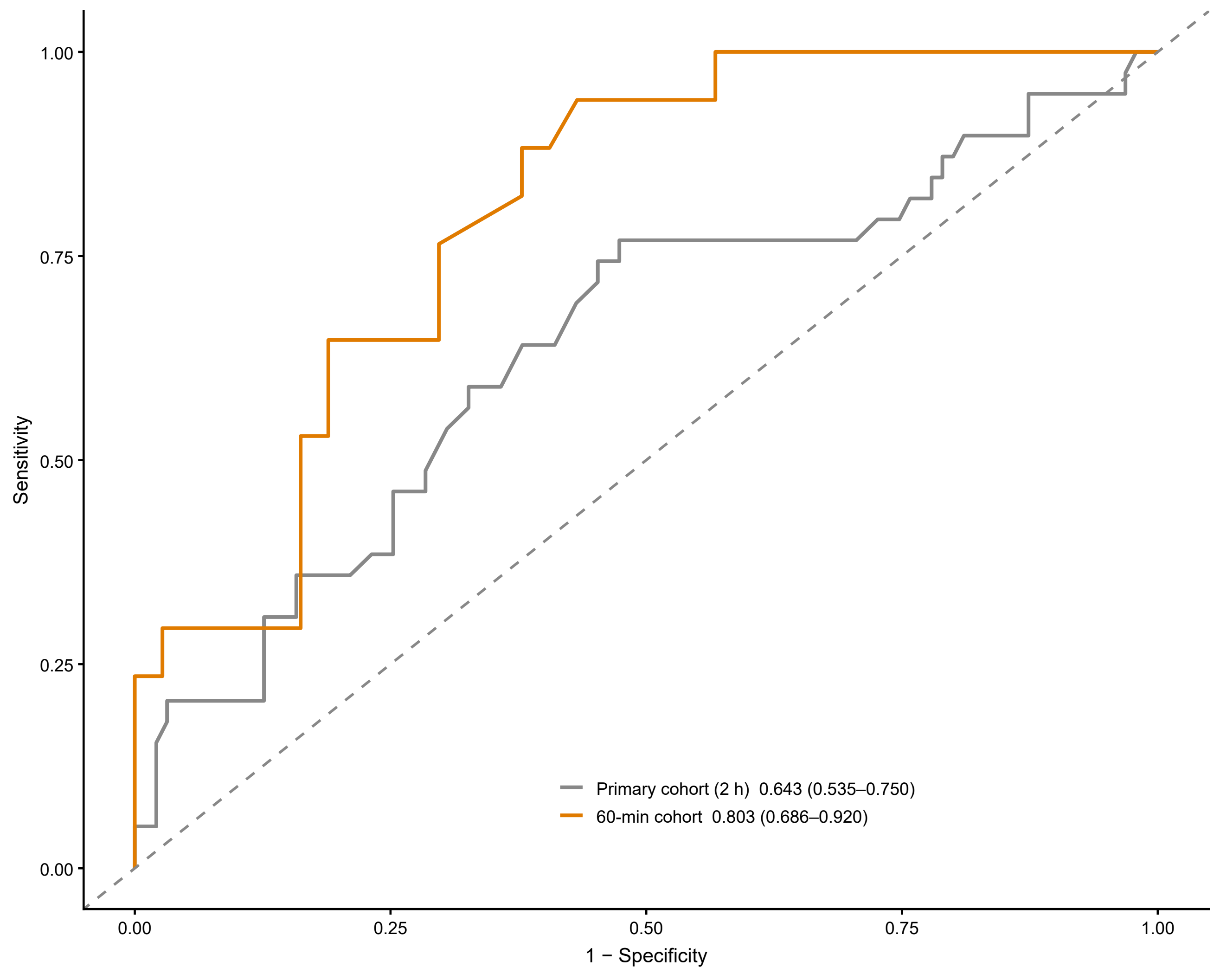

1.00
0.75
0.50
0.25
0.00
Sensitivity
0.00
0.25
0.50
0.75
1.00
1 − Specificity
Primary cohort (2 h) 0.643 (0.535–0.750)
60-min cohort 0.803 (0.686–0.920)
