# Supplementary material for: Alactic Base Excess as a Predictor of Mortality in Trauma ICU Patients: Retrospective Study and Evaluation of Integration into Trauma Scoring Systems
Source: Life (Basel). 2026 Oct 10;16(10):1701. doi: 10.3390/life16101701 (PMC13706226; doi:10.3390/life16101701)
Supplement: Supplementary file 1 [file life-16-01701-s001.zip › FigureS4.pdf]

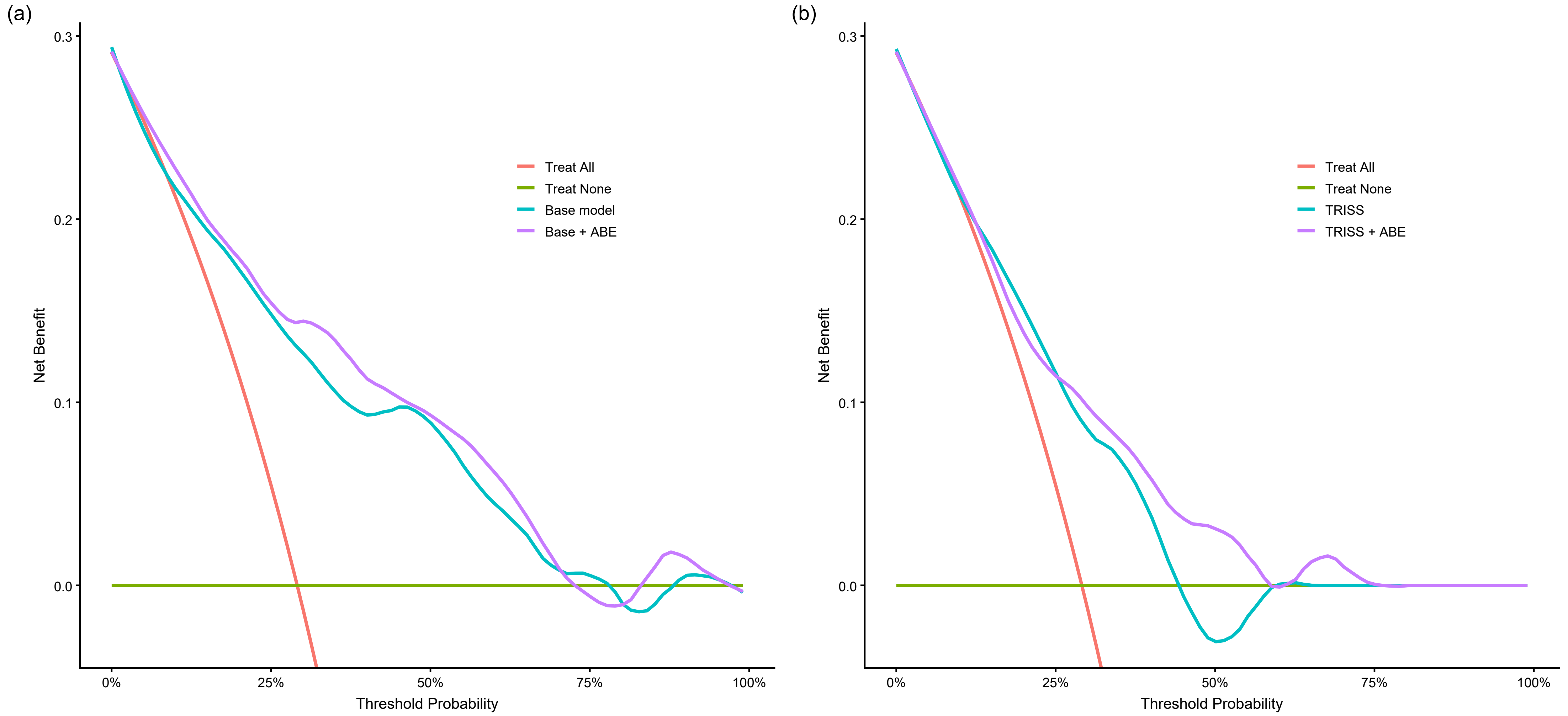

(a)
0.3
0.2
0.1
0.0
Net Benefit
0%
25%
50%
75%
100%
Threshold Probability
Treat All
Treat None
Base model
Base + ABE
(b)
0.3
0.2
0.1
0.0
Net Benefit
0%
25%
50%
75%
100%
Threshold Probability
Treat All
Treat None
TRISS
TRISS + ABE
