# Supplementary material for: Alactic Base Excess as a Predictor of Mortality in Trauma ICU Patients: Retrospective Study and Evaluation of Integration into Trauma Scoring Systems
Source: Life (Basel). 2026 Oct 10;16(10):1701. doi: 10.3390/life16101701 (PMC13706226; doi:10.3390/life16101701)
Supplement: Supplementary file 1 [file life-16-01701-s001.zip › FileS1.pdf]

# File S1. R analysis script

Schöllhorn FJ, Azamfirei L, Branea OE. Alactic Base Excess as a Predictor of Mortality in Trauma ICU Patients: Retrospective Study and Evaluation of Integration into Trauma Scoring Systems.

```
# ════════════════════════════════════════════════════════════
# Alactic Base Excess as a Predictor of Mortality in Trauma
# ICU Patients — R Analysis Script
#
# Authors: Schöllhorn FJ, Azamfirei L, Branea OE
# R version: 4.5.1
# File S1
# ════════════════════════════════════════════════════════════

# ==================================================================
# COMPLETE ANALYSIS SCRIPT
# ==================================================================
# Input and output folders are set under "USER SETTINGS" below.
#
# OUTPUT (in out_dir):
#   ABE_Analysis_Output.docx    (Word report with all tables)
#   Figures/                    (PNG — for the Word report)
#   Figures_PDF/                (PDF — print-quality figures)
#
# REQUIRED INPUT FILES (in data_dir):
#   1. cohort_final.csv                 primary cohort (n = 134)
#   2. PATIENTS_FINAL_120M.csv          120-min extraction
#   3. Patients_ICDPICR_60Minutes.xlsx  60-min sensitivity cohort
#   4. FlowchartCounts.csv              flow-diagram counts (Query 2, File S2)
#
# The input files are derived from MIMIC-IV v3.1 with the SQL queries in
# File S2. MIMIC-IV requires PhysioNet credentialed access, so the
# patient-level data are not redistributed here.

# ==================================================================

# ── 0. PACKAGES ──────────────────────────────────────────────
pkgs <- c("tidyverse","readxl","janitor","pROC","mice","gtsummary",
          "flextable","officer","ResourceSelection","lmtest","logistf","gridExtra")

new_pkgs <- pkgs[!pkgs %in% installed.packages()[,"Package"]]
if (length(new_pkgs)) install.packages(new_pkgs, repos="https://cloud.r-project.org")
invisible(lapply(pkgs, library, character.only=TRUE))

# ── USER SETTINGS ────────────────────────────────────────────
data_dir     <- "data"      # folder containing the four input files listed above
out_dir      <- "output"    # folder for the Word report and figures
fig_dir_word <- file.path(out_dir, "Figures")          # PNG  — embedded in Word
fig_dir_pdf  <- file.path(out_dir, "Figures_PDF")     # PDF — print-quality figures
dir.create(fig_dir_word, showWarnings=FALSE, recursive=TRUE)
dir.create(fig_dir_pdf,  showWarnings=FALSE, recursive=TRUE)

# ── FIGURE STYLE ─────────────────────────────────────────────
# Colour palette for publication figures
FIG_NAVY  <- "#0c385c"   # primary dark colour
FIG_BLUE  <- "#1770b8"   # primary light colour
FIG_MAUVE <- "#6b8ac5"   # secondary
FIG_LIGHT <- "#d0d9f0"   # accent / fills
FIG_AMBER <- "#e07b00"   # supplementary (for 3+ colour needs)
FIG_GRAY  <- "#888888"   # neutral / reference lines

# 6-colour palette for Figure 1 (individual markers)
FIG_PALETTE6 <- c(FIG_BLUE, FIG_NAVY, FIG_AMBER, FIG_MAUVE, FIG_GRAY, "#444444")

# Figure dimensions (single-column = 174 mm; double = 84 mm)
# Converted to inches for ggsave: 174 mm ≈ 6.85 in; 84 mm ≈ 3.31 in
FW_SINGLE <- 174 / 25.4   # 6.85 in — for ROC curves (single column)
FW_DOUBLE <-  84 / 25.4   # 3.31 in — for boxplot (double column)
FH_STD    <- 140 / 25.4   # 5.51 in — standard height (≤234 mm)

# Base theme: Helvetica/Arial, 9pt, clean
FIG_THEME <- theme_classic(base_size=9, base_family="sans") +
  theme(
    legend.background = element_rect(fill=alpha("white",0.85), colour=NA),
    legend.key.size   = unit(4, "mm"),
    legend.text       = element_text(size=7),
    axis.title        = element_text(size=8),
```

```
    axis.text         = element_text(size=7),
    plot.title        = element_blank(),   # no titles inside figures
    plot.subtitle     = element_blank()
  )

# Dual-save helper: PDF for print/submission, PNG for Word document.
# The PDF is written with cairo_pdf, which embeds the fonts and keeps
# Unicode symbols (e.g. <=, >=, en dashes, hyphens) intact; the default
# pdf() device drops or substitutes some of them.
save_fig <- function(plot, pdf_name, word_name,
                     width=FW_SINGLE, height=FH_STD) {
  ggsave(file.path(fig_dir_pdf,  paste0(pdf_name,  ".pdf")),
         plot, width=width, height=height, bg="white", device=cairo_pdf)
  ggsave(file.path(fig_dir_word, paste0(word_name, ".png")),
         plot, width=width, height=height, dpi=300, bg="white")
}

# ── MICE helper ───────────────────────────────────────────────
pool_glm <- function(formula, imp_obj, extra=NULL) {
  fits <- lapply(seq_len(imp_obj$m), function(i) {
    d <- complete(imp_obj, i)
    if (!is.null(extra)) for (nm in names(extra)) d[[nm]] <- extra[[nm]]
    glm(formula, data=d, family=binomial)
  })
  pool(fits)
}

# ── NRI + IDI bootstrap ───────────────────────────────────────
nri_idi_boot <- function(y, p_old, p_new, n_boot=500, seed=42) {
  nri_fn <- function(yb,pob,pnb) {
    ev <- mean(pnb[yb==1]>pob[yb==1]) - mean(pnb[yb==1]<pob[yb==1])
    ne <- mean(pnb[yb==0]<pob[yb==0]) - mean(pnb[yb==0]>pob[yb==0])
    c(nri=ev+ne, nri_plus=ev, nri_minus=ne)
  }
  idi_fn <- function(yb,pob,pnb)
    (mean(pnb[yb==1])-mean(pnb[yb==0])) -
    (mean(pob[yb==1])-mean(pob[yb==0]))
  pt_nri <- nri_fn(y,p_old,p_new)
  pt_idi <- idi_fn(y,p_old,p_new)
  set.seed(seed)
  boot <- replicate(n_boot, {
    idx <- sample(length(y),replace=TRUE)
    c(nri_fn(y[idx],p_old[idx],p_new[idx]),
      idi=idi_fn(y[idx],p_old[idx],p_new[idx]))
  })
  p0 <- function(b) max(2*min(mean(b<=0),mean(b>=0)), 1/length(b))
  list(nri=round(pt_nri["nri"],3), nri_plus=round(pt_nri["nri_plus"],3),
       nri_minus=round(pt_nri["nri_minus"],3),
       nri_ci=paste0(round(quantile(boot["nri",],0.025),3),"–",
                     round(quantile(boot["nri",],0.975),3)),
       nri_p=round(p0(boot["nri",]),3),
       idi=round(pt_idi,3),
       idi_ci=paste0(round(quantile(boot["idi",],0.025),3),"–",
                     round(quantile(boot["idi",],0.975),3)),
       idi_p=round(p0(boot["idi",]),3))
}

fmt_p <- function(p) ifelse(p < 0.001, "<0.001", sprintf("%.3f", p))

# ================================================================
# 1. LOAD MAIN COHORT
# ================================================================
cohort <- read_csv(file.path(data_dir, "cohort_final.csv"),
                   show_col_types=FALSE) %>%
  clean_names() %>%
  mutate(
    expire_inhosp = as.integer(died_inhosp=="Died"|died_inhosp==1),
    expire_30day  = as.integer(died_30day =="Died"|died_30day ==1),
    died_inhosp_f = factor(ifelse(expire_inhosp==1,"Died","Survived"),
                           levels=c("Survived","Died")),
    mechanism     = factor(mechanism,
                           levels=c("Blunt","Penetrating","Other/Unknown")),
    male_sex      = as.integer(gender %in% c("M","Male","male")),
    blunt_mech    = as.integer(mechanism=="Blunt")
  )

cat("── File 1 ───────────────────────────────────────────────────\n")
```

```r
cat("n=",nrow(cohort),
    "| In-hospital:",sum(cohort$expire_inhosp),
    sprintf("(%.1f%%)",mean(cohort$expire_inhosp)*100),
    "| 30-day:",sum(cohort$expire_30day),
    sprintf("(%.1f%%)\n",mean(cohort$expire_30day)*100))


# ==================================================================
# 1.5 PARTICIPANT FLOW DIAGRAM (FIGURE S2) + TABLE S2 (EXCLUDED-POPULATION CHARACTERISTICS)
# ==================================================================
# Reads the flowchart step counts exported from Query 2 (File S2;
# BigQuery; admission AND patient counts, computed via anti-joins for
# every box including the excluded ones), combines them with the live
# analytic cohort already loaded in this script, and renders a
# CONSORT-style flow diagram. Only the SQL-side steps come from the
# static Query 2 export; the final two boxes (ISS<16 excluded, final
# cohort) are computed live from `cohort`, so they cannot drift out of
# sync with the actual analysis.

library(ggplot2)
library(dplyr)
library(readr)

fc <- read_csv(file.path(data_dir, "FlowchartCounts.csv"), show_col_types = FALSE)

required_steps <- c(
  "1. Total Adult Admissions", "1b. Non-ICU admissions excluded",
  "2. ICU Admissions", "2b. No trauma ICD-10 code",
  "3. Trauma ICD-10 Code Present (S00-T88)", "3b. Missing ABG <=2h or incomplete data",
  "4. ABG <=2h + Complete Data Available", "4b. Exclusion diagnoses present",
  "5. Final Cohort (Exclusions Removed)"
)
missing_steps <- setdiff(required_steps, fc$step)
if (length(missing_steps) > 0) {
  stop(
    "FlowchartCounts.csv is missing ", length(missing_steps), " expected row(s):\n  ",
    paste(missing_steps, collapse = "\n  "),
    "\n\nThis usually means an outdated 5-row export was used instead of the ",
    "full 9-row output of Query 2 (File S2). Re-run Query 2 in BigQuery ",
    "and re-export the result as FlowchartCounts.csv before continuing."
  )
}

gv <- function(step) fc$admission_count[fc$step == step]
gp <- function(step) fc$patient_count[fc$step == step]
fmt <- function(x) format(x, big.mark = ",")


# Live final-cohort numbers (cohort_final.csv already has the ISS>=16 filter applied)
n_final <- nrow(cohort)
n_pre_iss_adm <- gv("5. Final Cohort (Exclusions Removed)")
n_pre_iss_pt  <- gp("5. Final Cohort (Exclusions Removed)")
n_excl_iss_adm <- n_pre_iss_adm - n_final
n_excl_iss_pt  <- n_pre_iss_pt - n_final

main_steps <- data.frame(
  label = c(
    sprintf("MIMIC-IV v3.1: Total Adult Admissions\nAdmissions: %s\nPatients: %s",
            fmt(gv("1. Total Adult Admissions")), fmt(gp("1. Total Adult Admissions"))),
    sprintf("ICU Admissions\nAdmissions: %s\nPatients: %s",
            fmt(gv("2. ICU Admissions")), fmt(gp("2. ICU Admissions"))),
    sprintf("Trauma ICD-10 Code Present (S00\u2013T88)\nAdmissions: %s\nPatients: %s",
            fmt(gv("3. Trauma ICD-10 Code Present (S00-T88)")), fmt(gp("3. Trauma ICD-10 Code Present (S00-T88)"))),
    sprintf("ABG \u22642 h + Complete Data Available\nAdmissions: %s\nPatients: %s",
            fmt(gv("4. ABG <=2h + Complete Data Available")), fmt(gp("4. ABG <=2h + Complete Data Available"))),
    sprintf("No Exclusion Diagnoses Present\nAdmissions: %s\nPatients: %s",
            fmt(gv("5. Final Cohort (Exclusions Removed)")), fmt(gp("5. Final Cohort (Exclusions Removed)"))),
    sprintf("Final Study Cohort (ISS \u226516)\nAdmissions: %s\nPatients: %s",
            fmt(n_final), fmt(n_final))
  ),
  y = seq(10, 0, length.out = 6),
  stringsAsFactors = FALSE
)
```

```
excl_steps <- data.frame(
  label = c(
    sprintf("Non-ICU admissions excluded\nAdmissions: %s\nPatients: %s",
            fmt(gv("1b. Non-ICU admissions excluded")), fmt(gp("1b. Non-ICU admissions excluded"))),
    sprintf("No trauma ICD-10 code (S00\u2013T88)\nAdmissions: %s\nPatients: %s",
            fmt(gv("2b. No trauma ICD-10 code")), fmt(gp("2b. No trauma ICD-10 code"))),
    sprintf("Missing ABG \u22642 h or incomplete data\nAdmissions: %s\nPatients: %s",
            fmt(gv("3b. Missing ABG <=2h or incomplete data")), fmt(gp("3b. Missing ABG <=2h or incomplete data"))),
    sprintf("Exclusion diagnoses present\nAdmissions: %s\nPatients: %s",
            fmt(gv("4b. Exclusion diagnoses present")), fmt(gp("4b. Exclusion diagnoses present"))),
    sprintf("ISS <16 (minor trauma)\nAdmissions: %s\nPatients: %s",
            fmt(n_excl_iss_adm), fmt(n_excl_iss_pt))
  ),
  y = main_steps$y[1:5] + diff(main_steps$y)[1] / 2,
  stringsAsFactors = FALSE
)

box_w <- 5.8; box_h <- 1.6
excl_box_w <- 5.2; excl_box_h <- 1.15

p <- ggplot() +
  geom_rect(data = main_steps,
            aes(xmin = -box_w/2, xmax = box_w/2, ymin = y - box_h/2, ymax = y + box_h/2),
            fill = FIG_LIGHT, colour = FIG_NAVY, linewidth = 0.5) +
  geom_text(data = main_steps, aes(x = 0, y = y, label = label),
            size = 2.6, lineheight = 0.9, family = "sans") +
  geom_rect(data = excl_steps,
            aes(xmin = box_w/2 + 0.6, xmax = box_w/2 + 0.6 + excl_box_w,
                ymin = y - excl_box_h/2, ymax = y + excl_box_h/2),
            fill = "#f2f2f2", colour = FIG_GRAY, linewidth = 0.4) +
  geom_text(data = excl_steps, aes(x = box_w/2 + 0.6 + excl_box_w/2, y = y, label = label),
            size = 2.3, lineheight = 0.9, family = "sans") +
  geom_segment(data = data.frame(y_start = main_steps$y[-nrow(main_steps)], y_end = main_steps$y[-1]),
               aes(x = 0, xend = 0, y = y_start - box_h/2, yend = y_end + box_h/2 + 0.05),
               arrow = arrow(length = unit(2, "mm"), type = "closed"), colour = FIG_NAVY, linewidth = 0.4) +
  geom_segment(data = data.frame(y = excl_steps$y),
               aes(x = 0, xend = box_w/2 + 0.55, y = y, yend = y),
               arrow = arrow(length = unit(2, "mm"), type = "closed"), colour = FIG_GRAY, linewidth = 0.35) +
  annotate("text", x = 0, y = -0.9, label = "(patients = unique patients across all admissions at that step)",
           size = 2.1, colour = "grey40", family = "sans") +
  coord_cartesian(xlim = c(-box_w/2 - 0.3, box_w/2 + 0.6 + excl_box_w + 0.3),
                  ylim = c(-1.3, 10.5 + box_h/2 + 0.3), expand = FALSE) +
  theme_void()

save_fig(p, "FigureS2", "FigureS2", width = FW_SINGLE * 1.55, height = FH_STD * 1.6)
cat(sprintf("Participant flow diagram saved. ISS<16 excluded: %d (%d pt) | Final cohort: %d\n",
            n_excl_iss_adm, n_excl_iss_pt, n_final))

# ==================================================================
# TABLE S2: CHARACTERISTICS OF PATIENTS EXCLUDED FOR MISSING EARLY ABG
# ==================================================================

# Admission and patient counts for the excluded group are taken from the
# same patient-level anti-join result used for the flow diagram
# (Figure S2), so Table S2 and Figure S2 always agree. A patient counts
# as excluded only if none of their admissions qualified.

excluded_n_adm   <- gv("3b. Missing ABG <=2h or incomplete data")
excluded_n_pt    <- gp("3b. Missing ABG <=2h or incomplete data")
excluded_med_age <- 64
excluded_pct_male <- 56.5
excluded_pct_mortality <- 11.9

final_n <- nrow(cohort)
final_med_age <- median(cohort$age, na.rm = TRUE)
final_pct_male <- round(100 * mean(cohort$male_sex, na.rm = TRUE), 1)
final_pct_mortality <- round(100 * mean(cohort$expire_inhosp, na.rm = TRUE), 1)

table_s2 <- tibble(
  Characteristic = c("Admissions, n", "Unique patients, n", "Median age, years",
                     "Male sex, %", "In-hospital mortality, %"),
  `Excluded (missing early ABG)` = c(
    format(excluded_n_adm, big.mark = ","),
    format(excluded_n_pt, big.mark = ","),
```

```r
    excluded_med_age,
    excluded_pct_male,
    excluded_pct_mortality
  ),
  `Final Cohort` = c(
    format(final_n, big.mark = ","),
    format(final_n, big.mark = ","),
    final_med_age,
    final_pct_male,
    final_pct_mortality
  )
)

print(table_s2)

# Export as a standalone Word table (Table S2), matching the ft_s() styling
# already used elsewhere in this script for Word output
ft_s2 <- flextable(as.data.frame(table_s2)) %>%
  theme_booktabs() %>%
  fontsize(size = 10, part = "all") %>%
  font(fontname = "Times New Roman", part = "all") %>%
  set_table_properties(width = 1, layout = "autofit") %>%
  bold(part = "header")

doc_s2 <- read_docx() %>%
  body_add_par("Table S2. Characteristics of patients excluded for missing an early arterial blood gas, compared with the final study cohort.", style = "heading 2") %>%
  body_add_flextable(ft_s2) %>%
  body_add_par("Excluded-group figures derived from Query 10 (File S2), run directly against MIMIC-IV v3.1 raw tables. Final-cohort figures computed from the analytic dataset (n=134).", style = "Normal")

print(doc_s2, target = file.path(out_dir, "TableS2_Excluded_Characteristics.docx"))

# Also export as a standalone PDF into the same Figures_PDF folder as the
# manuscript figures, matching the Table S1 (existing, external) convention.
tbl_s2_grob <- tableGrob(as.data.frame(table_s2), rows = NULL,
                         theme = ttheme_default(
                           core = list(fg_params = list(hjust = 0, x = 0.05, fontsize = 9)),
                           colhead = list(fg_params = list(fontface = "bold", fontsize = 9))
                         ))
ggsave(file.path(fig_dir_pdf, "TableS2.pdf"), plot = tbl_s2_grob, width = 7, height = 2, bg = "white",
       device = cairo_pdf)

cat(sprintf("Table S2 saved (Word + PDF). Excluded: n=%s adm / %s pt | Final cohort: n=%d\n",
            format(excluded_n_adm, big.mark=","), format(excluded_n_pt, big.mark=","), final_n))


# ================================================================
# 2. LOAD 120M FILE
# ================================================================
cat("\n── File 2: PATIENTS_FINAL_120M.csv ────────────────────────\n")
raw_line <- readLines(file.path(data_dir, "PATIENTS_FINAL_120M.csv"),
                      n=1,encoding="UTF-8",warn=FALSE)
n_semi  <- nchar(raw_line)-nchar(gsub(";","",raw_line))
n_comma <- nchar(raw_line)-nchar(gsub(",","",raw_line))
n_tab   <- nchar(raw_line)-nchar(gsub("\t","",raw_line))
delim_char <- if (n_semi>n_comma&&n_semi>n_tab)";" else
              if (n_tab>n_comma)"\t" else ","
cat("Delimiter:",shQuote(delim_char),"\n")

pts_120m_raw <- read_delim(file.path(data_dir, "PATIENTS_FINAL_120M.csv"),
  delim=delim_char,show_col_types=FALSE,
  locale=locale(encoding="UTF-8")) %>% clean_names()

dx_cols <- grep("^dx",names(pts_120m_raw),value=TRUE,ignore.case=TRUE)
sep_vec <- if (length(dx_cols)>0) {
  as.integer(rowSums(sapply(dx_cols,function(col) {
    vals <- pts_120m_raw[[col]]
    !is.na(vals)&grepl("A40|A41|R6520|R6521",vals,ignore.case=TRUE)
  }))>0)
} else rep(0L,nrow(pts_120m_raw))
cat("Sepsis cases:",sum(sep_vec),"\n")

pts_120m <- pts_120m_raw %>%
  mutate(
    lab_p = parse_date_time(lab_time, orders=c("ymd HMS","ymd HM","ymd")),
    adm_p = parse_date_time(admittime,orders=c("ymd HMS","ymd HM","ymd")),
```

```r
    abg_minutes = as.numeric(difftime(lab_p,adm_p,units="mins")),
    sepsis_flag = sep_vec
  ) %>% select(subject_id,hadm_id,abg_minutes,sepsis_flag)

cohort <- cohort %>% left_join(pts_120m,by=c("subject_id","hadm_id"))
cat("abg_minutes:",
    round(min(cohort$abg_minutes,na.rm=TRUE)),"–",
    round(max(cohort$abg_minutes,na.rm=TRUE)),"min\n")

# ================================================================
# 3. LOAD 60-MINUTE COHORT
# ================================================================
cat("\n── File 3: Patients_ICDPICR_60Minutes.xlsx ─────────────────\n")
pts_60m <- readxl::read_xlsx(file.path(data_dir, "Patients_ICDPICR_60Minutes.xlsx")) %>%
  clean_names() %>%
  select(subject_id,hadm_id,
         abe_60m=alactic_base_excess,
         be_60m=base_excess,lac_60m=lactate)

cohort_1hr <- cohort %>%
  inner_join(pts_60m,by=c("subject_id","hadm_id")) %>%
  mutate(alactic_base_excess=as.numeric(abe_60m),
         base_excess=as.numeric(be_60m),
         lactate=as.numeric(lac_60m))

# Clean date-format artefacts
n_bad_abe <- sum(cohort_1hr$alactic_base_excess > 100 |
                 cohort_1hr$alactic_base_excess < -100, na.rm=TRUE)
n_bad_lac <- sum(cohort_1hr$lactate < 0 | cohort_1hr$lactate > 100, na.rm=TRUE)
cat(sprintf("Date-artifact values set to NA: ABE=%d, Lactate=%d\n",
            n_bad_abe, n_bad_lac))

cohort_1hr <- cohort_1hr %>%
  mutate(
    alactic_base_excess = if_else(alactic_base_excess > 100 | alactic_base_excess < -100,
                                  NA_real_, alactic_base_excess),
    base_excess         = if_else(base_excess > 100 | base_excess < -100,
                                  NA_real_, base_excess),
    lactate             = if_else(lactate < 0 | lactate > 100, NA_real_, lactate)
  )

cohort_nosep <- cohort %>% filter(!is.na(sepsis_flag)&sepsis_flag==0)

# ── N-VERIFICATION: 60-minute effective analytic sample ──────────
# Reports the effective analytic n after exclusion of date-format artefacts
n_total_60m    <- nrow(cohort_1hr)
n_valid_abe_60m<- sum(!is.na(cohort_1hr$alactic_base_excess))
n_valid_lac_60m<- sum(!is.na(cohort_1hr$lactate))
n_complete_60m <- sum(!is.na(cohort_1hr$alactic_base_excess) &
                       !is.na(cohort_1hr$lactate) &
                       !is.na(cohort_1hr$expire_inhosp))
cat(sprintf("\n── 60-min cohort n-verification ────────────────────────────\n"))
cat(sprintf("  Total in 60-min file:                %d\n",  n_total_60m))
cat(sprintf("  Valid ABE (after artefact exclusion): %d\n",  n_valid_abe_60m))
cat(sprintf("  Valid lactate:                        %d\n",  n_valid_lac_60m))
cat(sprintf("  Complete cases (ABE + lactate + outcome): %d  <-- effective analytic n\n",
            n_complete_60m))

cat(sprintf("60-min: n=%d | deaths=%d | Correlation ABE-outcome: %.3f\n",
            nrow(cohort_1hr), sum(cohort_1hr$expire_inhosp),
            cor(cohort_1hr$alactic_base_excess,
                cohort_1hr$expire_inhosp, use="complete.obs")))
cat("No-sepsis: n=",nrow(cohort_nosep),"| deaths:",sum(cohort_nosep$expire_inhosp),"\n")

# ================================================================
# 4. TABLE 1 — BASELINE CHARACTERISTICS
# ================================================================
pvals_t1 <- list(
  age                 = wilcox.test(cohort$age                 ~ cohort$expire_inhosp)$p.value,
  male_sex            = chisq.test( table(cohort$male_sex,   cohort$expire_inhosp), correct=FALSE)$p.value,
  blunt_mech          = chisq.test( table(cohort$blunt_mech, cohort$expire_inhosp), correct=FALSE)$p.value,
  riss                = wilcox.test(cohort$riss                ~ cohort$expire_inhosp)$p.value,
  niss                = wilcox.test(cohort$niss                ~ cohort$expire_inhosp)$p.value,
  alactic_base_excess = wilcox.test(cohort$alactic_base_excess ~ cohort$expire_inhosp)$p.value,
  base_excess         = wilcox.test(cohort$base_excess         ~ cohort$expire_inhosp)$p.value,
  lactate             = wilcox.test(cohort$lactate             ~ cohort$expire_inhosp)$p.value,
  gcs_total           = wilcox.test(cohort$gcs_total ~ cohort$expire_inhosp, na.action=na.omit)$p.value,
```

```
  sbp                 = wilcox.test(cohort$sbp       ~ cohort$expire_inhosp, na.action=na.omit)$p.value,
  hospital_los_days   = wilcox.test(cohort$hospital_los_days   ~ cohort$expire_inhosp)$p.value
)
cat("\nTable 1 p-values (base R):\n")
for (nm in names(pvals_t1)) cat(sprintf("  %-25s %s\n", nm, fmt_p(pvals_t1[[nm]])))

tbl1 <- cohort %>%
  select(died_inhosp_f,age,male_sex,blunt_mech,riss,niss,
         alactic_base_excess,base_excess,lactate,
         gcs_total,sbp,hospital_los_days) %>%
  tbl_summary(
    by=died_inhosp_f,
    statistic=list(all_continuous()  ~"{median} [{p25}–{p75}]",
                   all_categorical() ~"{n} ({p}%)"),
    digits=list(all_continuous()  ~2, all_categorical()~c(0,1)),
    type =list(male_sex~"dichotomous",blunt_mech~"dichotomous"),
    value=list(male_sex~1,blunt_mech~1),
    label=list(
      age                 ~"Age, years",
      male_sex            ~"Male sex",
      blunt_mech          ~"Blunt mechanism",
      riss                ~"ISS",
      niss                ~"NISS",
      alactic_base_excess ~"ABE, mmol/L",
      base_excess         ~"Base excess, mmol/L",
      lactate             ~"Lactate, mmol/L",
      gcs_total           ~"GCS",
      sbp                 ~"SBP, mmHg",
      hospital_los_days   ~"Hospital LOS, days"),
    missing="no"
  ) %>%
  add_p(test=list(all_continuous()~"kruskal.test",
                  all_categorical()~"chisq.test")) %>%
  add_overall() %>%
  modify_header(p.value="**p-value**")

if ("p.value" %in% names(tbl1$table_body)) {
  tbl1$table_body$p.value <- suppressWarnings(as.numeric(tbl1$table_body$p.value))
  for (nm in names(pvals_t1)) {
    rows <- which(tbl1$table_body$variable == nm &
                  tbl1$table_body$row_type  == "label")
    if (length(rows) > 0)
      tbl1$table_body$p.value[rows[1]] <- pvals_t1[[nm]]
  }
  cat("p-values injected (numeric)\n")
}

mortality_tbl <- tibble(
  Outcome=c("In-hospital mortality","30-day mortality"),
  `n (%)`=c(sprintf("%d (%.1f%%)",sum(cohort$expire_inhosp),mean(cohort$expire_inhosp)*100),
            sprintf("%d (%.1f%%)",sum(cohort$expire_30day),mean(cohort$expire_30day)*100))
)

# ==================================================================
# 5. ABE DISTRIBUTION
# ==================================================================
abe_min <- min(cohort$alactic_base_excess,na.rm=TRUE)
abe_max <- max(cohort$alactic_base_excess,na.rm=TRUE)
abe_med <- median(cohort$alactic_base_excess,na.rm=TRUE)
abe_q1  <- quantile(cohort$alactic_base_excess,0.25,na.rm=TRUE)
abe_q3  <- quantile(cohort$alactic_base_excess,0.75,na.rm=TRUE)

abe_dist_tbl <- tibble(
  Statistic=c("Minimum","Q1","Median","Q3","Maximum","IQR"),
  `mmol/L`=round(c(abe_min,abe_q1,abe_med,abe_q3,abe_max,abe_q3-abe_q1),2)
)

smd_by <- function(x, grp) {
  g  <- if (is.factor(grp)) as.character(grp) else as.character(as.integer(grp))
  d  <- data.frame(x=x,g=g) %>% drop_na()
  e1 <- if (is.factor(grp)) "Died" else "1"
  e0 <- if (is.factor(grp)) "Survived" else "0"
  x1 <- d$x[d$g==e1]; x0 <- d$x[d$g==e0]
  sp <- sqrt(((length(x1)-1)*var(x1)+(length(x0)-1)*var(x0)) /
               (length(x1)+length(x0)-2))
  round(abs((mean(x1)-mean(x0))/sp),3)
}
```

```
roc_abe     <- roc(cohort$expire_inhosp,cohort$alactic_base_excess,ci=TRUE,quiet=TRUE)
roc_abe_30d <- roc(cohort$expire_30day, cohort$alactic_base_excess,ci=TRUE,quiet=TRUE)

youden     <- coords(roc_abe,    "best",best.method="youden",
                     ret=c("threshold","sensitivity","specificity","ppv","npv"))
youden_30d <- coords(roc_abe_30d,"best",best.method="youden",
                     ret=c("threshold","sensitivity","specificity","ppv","npv"))

cat(sprintf("\nYouden (in-hosp): %.2f | Sens %.1f%% | Spec %.1f%% | NPV %.1f%%\n",
    youden$threshold,youden$sensitivity*100,youden$specificity*100,youden$npv*100))
cat(sprintf("Youden (30-day):  %.2f | Sens %.1f%% | Spec %.1f%% | NPV %.1f%%\n",
    youden_30d$threshold,youden_30d$sensitivity*100,
    youden_30d$specificity*100,youden_30d$npv*100))

smd_tbl <- tibble(
  Marker=c("ABE","Lactate","Base Excess"),
  `SMD (in-hospital)`=c(
    smd_by(cohort$alactic_base_excess,cohort$died_inhosp_f),
    smd_by(cohort$lactate,            cohort$died_inhosp_f),
    smd_by(cohort$base_excess,        cohort$died_inhosp_f)),
  `SMD (30-day)`=c(
    smd_by(cohort$alactic_base_excess,cohort$expire_30day),
    smd_by(cohort$lactate,            cohort$expire_30day),
    smd_by(cohort$base_excess,        cohort$expire_30day)),
  `p (in-hospital)`=c(
    fmt_p(wilcox.test(cohort$alactic_base_excess~cohort$expire_inhosp)$p.value),
    fmt_p(wilcox.test(cohort$lactate            ~cohort$expire_inhosp)$p.value),
    fmt_p(wilcox.test(cohort$base_excess        ~cohort$expire_inhosp)$p.value)),
  `p (30-day)`=c(
    fmt_p(wilcox.test(cohort$alactic_base_excess~cohort$expire_30day)$p.value),
    fmt_p(wilcox.test(cohort$lactate            ~cohort$expire_30day)$p.value),
    fmt_p(wilcox.test(cohort$base_excess        ~cohort$expire_30day)$p.value))
)

threshold_tbl <- tibble(
  Outcome=c("In-hospital mortality","30-day mortality"),
  Threshold=c(round(youden$threshold,2),round(youden_30d$threshold,2)),
  Sensitivity=paste0(round(c(youden$sensitivity,youden_30d$sensitivity)*100,1),"%"),
  Specificity=paste0(round(c(youden$specificity,youden_30d$specificity)*100,1),"%"),
  PPV=paste0(round(c(youden$ppv,youden_30d$ppv)*100,1),"%"),
  NPV=paste0(round(c(youden$npv,youden_30d$npv)*100,1),"%"),
  `ABE p (Mann-Whitney)`=c(
    round(wilcox.test(alactic_base_excess~expire_inhosp,data=cohort)$p.value,3),
    round(wilcox.test(alactic_base_excess~expire_30day, data=cohort)$p.value,3))
)

# ================================================================
# 6. ROC – INDIVIDUAL MARKERS
# ================================================================
roc_be    <- roc(cohort$expire_inhosp,cohort$base_excess, ci=TRUE,quiet=TRUE)
roc_lac   <- roc(cohort$expire_inhosp,cohort$lactate,     ci=TRUE,quiet=TRUE)
roc_iss   <- roc(cohort$expire_inhosp,cohort$riss,        ci=TRUE,quiet=TRUE)
roc_niss  <- roc(cohort$expire_inhosp,cohort$niss,        ci=TRUE,quiet=TRUE)
roc_triss <- roc(cohort$expire_inhosp,cohort$triss_mort,  ci=TRUE,quiet=TRUE)
roc_list  <- list(ABE=roc_abe,BE=roc_be,Lactate=roc_lac,
                  ISS=roc_iss,NISS=roc_niss,TRISS=roc_triss)

auc_tbl <- imap_dfr(roc_list,~tibble(
  Marker=.y, AUC=round(as.numeric(auc(.x)),3),
  `95% CI`=paste0(round(ci(.x)[1],3),"–",round(ci(.x)[3],3))
))
delong_tbl <- tibble(
  Comparison=c("ABE vs BE","ABE vs Lactate","ABE vs ISS","ABE vs NISS","ABE vs TRISS"),
  DeLong_p=suppressWarnings(round(c(
    roc.test(roc_abe,roc_be,   method="delong")$p.value,
    roc.test(roc_abe,roc_lac,  method="delong")$p.value,
    roc.test(roc_abe,roc_iss,  method="delong")$p.value,
    roc.test(roc_abe,roc_niss, method="delong")$p.value,
    roc.test(roc_abe,roc_triss,method="delong")$p.value),3))
)

# ================================================================
# 7. MULTIPLE IMPUTATION
# ================================================================
imp_data <- cohort %>%
  select(expire_inhosp,alactic_base_excess,base_excess,lactate,
```

```
        age,riss,niss,mechanism,gcs_total,sbp)
cat("\n── Missing data (%) ─────────────────────────────────────────\n")
print(round(colSums(is.na(imp_data))/nrow(imp_data)*100,1))
set.seed(42)
imp <- mice(imp_data,m=20,method="pmm",printFlag=FALSE,seed=42)

# ================================================================
# 8. UNIVARIABLE LOGISTIC REGRESSION
# ================================================================
var_labels <- c(
  alactic_base_excess="ABE (per 1 mmol/L)",
  base_excess="Base excess (per 1 mmol/L)",
  lactate="Lactate (per 1 mmol/L)",
  age="Age (per year)",niss="NISS (per point)",
  riss="ISS (per point)",gcs_total="GCS total",sbp="SBP (per mmHg)"
)
univ_tbl <- map_dfr(names(var_labels),function(v) {
  pooled <- pool_glm(reformulate(v,response="expire_inhosp"),imp)
  summary(pooled,conf.int=TRUE,exponentiate=TRUE) %>%
    filter(term!="(Intercept)") %>%
    transmute(Variable=var_labels[v],OR=round(estimate,3),
              `95% CI`=paste0(round(`2.5 %`,3),"–",round(`97.5 %`,3)),
              p=round(p.value,3)) %>%
    mutate(`OR (95% CI)`=paste0(OR," (",`95% CI`,")")) %>%
    select(Variable,`OR (95% CI)`,p)
})
cat("\n── Univariable ABE:",univ_tbl$`OR (95% CI)`[1],"p=",univ_tbl$p[1],"\n")

# ================================================================
# 9. MULTIVARIABLE MODELS + RAW COEFFICIENTS
# ================================================================
fit_m1    <- pool_glm(expire_inhosp~alactic_base_excess+age+riss+mechanism,imp)
fit_m2    <- pool_glm(expire_inhosp~alactic_base_excess+age+niss+mechanism,imp)
fit_m3    <- pool_glm(expire_inhosp~alactic_base_excess+age+niss+lactate+mechanism,imp)
fit_m2_30 <- pool_glm(expire_30day~alactic_base_excess+age+niss+mechanism,imp,
                      extra=list(expire_30day=cohort$expire_30day))

s_m2   <- summary(fit_m2,conf.int=TRUE,exponentiate=TRUE)
abe_m2 <- s_m2[s_m2$term=="alactic_base_excess",]
cat("── OR check: M2",round(abe_m2$estimate,3),
    "(",round(abe_m2$`2.5 %`,3),"–",round(abe_m2$`97.5 %`,3),
    ") p=",round(abe_m2$p.value,3),"\n")

# Raw coefficients including intercept
cat("\n── Raw coefficients (primary model, fit_m2) ─────────────────\n")
s_raw_m2 <- summary(fit_m2, conf.int=TRUE, exponentiate=FALSE)
print(round(s_raw_m2[, c("term","estimate","std.error","2.5 %","97.5 %","p.value")], 4))

get_coef <- function(s, term_name) s$estimate[s$term == term_name]
get_se   <- function(s, term_name) s$std.error[s$term == term_name]

b0      <- get_coef(s_raw_m2, "(Intercept)")
b_abe   <- get_coef(s_raw_m2, "alactic_base_excess")
b_age   <- get_coef(s_raw_m2, "age")
b_niss  <- get_coef(s_raw_m2, "niss")
b_pen   <- get_coef(s_raw_m2, "mechanismPenetrating")
b_other <- get_coef(s_raw_m2, "mechanismOther/Unknown")
se_b0   <- get_se(s_raw_m2,   "(Intercept)")

cat(sprintf("\nIntercept (β₀): %.4f (SE %.4f)\n", b0, se_b0))
cat(sprintf("Formula: L = %.4f + (%.4f×ABE) + (%.4f×Age) + (%.4f×NISS) + (%.4f×Pen) + (%.4f×Other)\n",
            b0, b_abe, b_age, b_niss, b_pen, b_other))

compute_prob <- function(abe, age, niss, mech="blunt") {
  pen   <- as.integer(mech=="penetrating")
  other <- as.integer(mech=="other")
  L <- b0 + b_abe*abe + b_age*age + b_niss*niss + b_pen*pen + b_other*other
  list(logit=round(L,4), prob=round(1/(1+exp(-L))*100,1))
}
ex1 <- compute_prob(-4.0,50,34,"blunt")
ex2 <- compute_prob(-1.0,35,25,"blunt")
ex3 <- compute_prob(-8.0,65,45,"penetrating")
cat(sprintf("Ex1 (ABE=−4, Age=50, NISS=34, blunt):       L=%.4f → P(death)=%.1f%%\n",ex1$logit,ex1$prob))
cat(sprintf("Ex2 (ABE=−1, Age=35, NISS=25, blunt):       L=%.4f → P(death)=%.1f%%\n",ex2$logit,ex2$prob))
cat(sprintf("Ex3 (ABE=−8, Age=65, NISS=45, penetrating): L=%.4f → P(death)=%.1f%%\n",ex3$logit,ex3$prob))

coef_tbl <- s_raw_m2 %>%
```

```
  mutate(Variable=case_when(
    term=="(Intercept)"             ~"Intercept (β₀)",
    term=="alactic_base_excess"     ~"ABE (per 1 mmol/L)",
    term=="age"                     ~"Age (per year)",
    term=="niss"                    ~"NISS (per point)",
    term=="mechanismPenetrating"    ~"Penetrating mechanism (vs. blunt)",
    term=="mechanismOther/Unknown"  ~"Other/unknown mechanism (vs. blunt)",
    TRUE~term)) %>%
  transmute(
    Variable,
    `β (SE)` = sprintf("%.4f (%.4f)", estimate, std.error),
    OR       = ifelse(term=="(Intercept)","—",sprintf("%.3f", exp(estimate))),
    `95% CI (OR)` = ifelse(term=="(Intercept)","—",
                        sprintf("%.3f–%.3f", exp(`2.5 %`), exp(`97.5 %`))),
    `p-value`= fmt_p(p.value)
  )

example_tbl <- tibble(
  Example=c("Moderate severity, blunt","Mild derangement, blunt","Severe derangement, penetrating"),
  ABE=c(-4.0,-1.0,-8.0), Age=c(50,35,65), NISS=c(34,25,45),
  Mechanism=c("Blunt","Blunt","Penetrating"),
  `Logit (L)`=c(ex1$logit,ex2$logit,ex3$logit),
  `P(death) %`=c(ex1$prob,ex2$prob,ex3$prob)
)

fmt_pool <- function(pooled, label) {
  summary(pooled,conf.int=TRUE,exponentiate=TRUE) %>%
    filter(term!="(Intercept)") %>%
    transmute(
      Model=label,
      Variable=case_when(
        term=="alactic_base_excess"     ~"ABE (per 1 mmol/L)",
        term=="age"                     ~"Age (per year)",
        term=="riss"                    ~"ISS (per point)",
        term=="niss"                    ~"NISS (per point)",
        term=="lactate"                 ~"Lactate (per 1 mmol/L)",
        term=="mechanismPenetrating"    ~"Penetrating (vs. blunt)",
        term=="mechanismOther/Unknown"  ~"Other/unknown (vs. blunt)",
        TRUE~term),
      OR=round(estimate,3),
      `95% CI`=paste0(round(`2.5 %`,3),"–",round(`97.5 %`,3)),
      p=round(p.value,3))
}

multiv_tbl <- bind_rows(
  fmt_pool(fit_m1,"Model 1 (ISS)"),
  fmt_pool(fit_m2,"Model 2 — Primary (NISS)"),
  fmt_pool(fit_m3,"Model 3 (NISS + Lactate)")
)

make_primary_tbl <- function(pooled) {
  row_order <- c("ABE (per 1 mmol/L increase)","Age (per year)","NISS (per point)",
                 "Penetrating mechanism (vs. blunt)","Other/unknown mechanism (vs. blunt)")
  summary(pooled,conf.int=TRUE,exponentiate=TRUE) %>%
    filter(term!="(Intercept)") %>%
    mutate(Variable=case_when(
      term=="alactic_base_excess"    ~"ABE (per 1 mmol/L increase)",
      term=="age"                    ~"Age (per year)",
      term=="niss"                   ~"NISS (per point)",
      term=="mechanismPenetrating"   ~"Penetrating mechanism (vs. blunt)",
      term=="mechanismOther/Unknown" ~"Other/unknown mechanism (vs. blunt)",
      TRUE~term)) %>%
    transmute(Variable,OR=round(estimate,3),
              `95% CI`=paste0(round(`2.5 %`,3),"–",round(`97.5 %`,3)),
              `p-value`=round(p.value,3)) %>%
    slice(match(intersect(row_order,Variable),Variable))
}
primary_tbl_ih <- make_primary_tbl(fit_m2)
primary_tbl_30 <- make_primary_tbl(fit_m2_30)

# ==================================================================
# 10. HOSMER-LEMESHOW (ALL FOUR MODELS)
# ==================================================================
m1_cc          <- glm(expire_inhosp~alactic_base_excess+age+riss+mechanism,data=cohort,family=binomial)
m2_cc          <- glm(expire_inhosp~alactic_base_excess+age+niss+mechanism,data=cohort,family=binomial)
m3_cc          <-
glm(expire_inhosp~alactic_base_excess+age+niss+lactate+mechanism,data=cohort,family=binomial)
```

```
m_triss_abe_cc <- glm(expire_inhosp~triss_mort+alactic_base_excess,data=cohort,family=binomial)

hl_m1        <- hoslem.test(cohort$expire_inhosp,fitted(m1_cc),        g=10)
hl_m2        <- hoslem.test(cohort$expire_inhosp,fitted(m2_cc),        g=10)
hl_m3        <- hoslem.test(cohort$expire_inhosp,fitted(m3_cc),        g=10)
hl_triss_abe <- hoslem.test(cohort$expire_inhosp,fitted(m_triss_abe_cc),g=10)

hl_tbl <- tibble(
  Model=c("Model 1 (ISS)","Model 2 – Primary (NISS)",
          "Model 3 (NISS + Lactate)","TRISS + ABE"),
  `χ²`=round(c(hl_m1$statistic,hl_m2$statistic,hl_m3$statistic,hl_triss_abe$statistic),2),
  df  =c(hl_m1$parameter,hl_m2$parameter,hl_m3$parameter,hl_triss_abe$parameter),
  p   =round(c(hl_m1$p.value,hl_m2$p.value,hl_m3$p.value,hl_triss_abe$p.value),3),
  Calibration=ifelse(c(hl_m1$p.value,hl_m2$p.value,hl_m3$p.value,hl_triss_abe$p.value)>0.05,
                     "Adequate","Poor")
)
cat("\n── Hosmer-Lemeshow ─────────────────────────────────────────\n")
print(as.data.frame(hl_tbl))

# ================================================================

# ================================================================
# 10.5. EXTENDED CALIBRATION METRICS
# ================================================================
# Brier score, calibration-in-the-large (CITL), calibration slope
# and calibration plot (Figure S1) for the primary model (m2_cc).

pred_m2  <- fitted(m2_cc)
obs_m2   <- as.integer(cohort$expire_inhosp)
logit_m2 <- log(pred_m2 / (1 - pred_m2))

# Brier score
brier_score  <- mean((pred_m2 - obs_m2)^2)
brier_null   <- mean(obs_m2) * (1 - mean(obs_m2))
brier_scaled <- 1 - (brier_score / brier_null)

# Calibration-in-the-large: outcome ~ offset(logit(pred)), intercept should be ~0
citl_model <- glm(obs_m2 ~ 1, offset=logit_m2, family=binomial)
citl_val   <- coef(citl_model)[1]
citl_ci    <- suppressMessages(confint(citl_model))

# Calibration slope: outcome ~ logit(pred), slope should be ~1
cslope_model  <- glm(obs_m2 ~ logit_m2, family=binomial)
cal_slope     <- coef(cslope_model)[2]
cal_int_val   <- coef(cslope_model)[1]
cal_slope_ci  <- suppressMessages(confint(cslope_model)["logit_m2",])

cat("\n── Extended Calibration (Primary Model, complete case n=134) ────\n")
cat(sprintf("Brier score:            %.4f (null=%.4f)\n", brier_score, brier_null))
cat(sprintf("Scaled Brier:           %.4f (0=no skill; higher=better)\n", brier_scaled))
cat(sprintf("CITL:                   %.4f (95%% CI %.4f to %.4f; ideal=0)\n",
            citl_val, citl_ci[1], citl_ci[2]))
cat(sprintf("Calibration slope:      %.4f (95%% CI %.4f to %.4f; ideal=1)\n",
            cal_slope, cal_slope_ci[1], cal_slope_ci[2]))

cal_metrics_tbl <- tibble(
  Metric         = c("Brier score","Scaled Brier score (0\u2013+1)",
                     "Calibration-in-the-large (CITL)","Calibration slope"),
  Value          = round(c(brier_score, brier_scaled, citl_val, cal_slope), 4),
  `95% CI`       = c("–","–",
                     sprintf("%.4f to %.4f", citl_ci[1], citl_ci[2]),
                     sprintf("%.4f to %.4f", cal_slope_ci[1], cal_slope_ci[2])),
  `Ideal value`  = c("<0.25",">0","0","1"),
  Note           = c("Mean squared error of predictions",
                     "Skill vs. null intercept-only model",
                     "Systematic over/under-prediction",
                     "Spread; <1 = overfit/overconfident")
)

# ================================================================
# 10.6. BOOTSTRAP OPTIMISM CORRECTION
# ================================================================
# Harrell's 500-iteration bootstrap for the apparent C-statistic.
# Primary model only; all primary predictors are complete (n=134)
# so bootstrap is performed on complete-case data without imputation.

boot_cc <- cohort %>%
```

```
  filter(!is.na(alactic_base_excess),!is.na(age),!is.na(niss),
         !is.na(mechanism),!is.na(expire_inhosp))
n_boot_cc <- nrow(boot_cc)   # should equal 134

apparent_abe_auc  <- as.numeric(auc(roc(
  boot_cc$expire_inhosp,
  fitted(glm(expire_inhosp~alactic_base_excess+age+niss+mechanism,
             data=boot_cc,family=binomial)), quiet=TRUE)))

apparent_base_auc <- as.numeric(auc(roc(
  boot_cc$expire_inhosp,
  fitted(glm(expire_inhosp~age+niss+mechanism,
             data=boot_cc,family=binomial)), quiet=TRUE)))

set.seed(42)
N_OPT <- 500
opt_abe  <- rep(NA_real_, N_OPT)
opt_base <- rep(NA_real_, N_OPT)

for (b in seq_len(N_OPT)) {
  idx     <- sample(n_boot_cc, replace=TRUE)
  d_boot  <- boot_cc[idx, ]
  d_orig  <- boot_cc
  tryCatch({
    # Primary model (with ABE)
    fb_abe  <- glm(expire_inhosp~alactic_base_excess+age+niss+mechanism,
                   data=d_boot,family=binomial)
    opt_abe[b]  <- as.numeric(auc(roc(d_boot$expire_inhosp,fitted(fb_abe),quiet=TRUE))) -
                   as.numeric(auc(roc(d_orig$expire_inhosp,
                                      predict(fb_abe,newdata=d_orig,type="response"),quiet=TRUE)))
    # Base model (without ABE)
    fb_base <- glm(expire_inhosp~age+niss+mechanism,data=d_boot,family=binomial)
    opt_base[b] <- as.numeric(auc(roc(d_boot$expire_inhosp,fitted(fb_base),quiet=TRUE))) -
                   as.numeric(auc(roc(d_orig$expire_inhosp,
                                      predict(fb_base,newdata=d_orig,type="response"),quiet=TRUE)))
  }, error=function(e) NULL)
}

opt_abe  <- na.omit(opt_abe)
opt_base <- na.omit(opt_base)
mean_opt_abe  <- mean(opt_abe)
mean_opt_base <- mean(opt_base)
auc_corr_abe  <- apparent_abe_auc  - mean_opt_abe
auc_corr_base <- apparent_base_auc - mean_opt_base

cat(sprintf("\n── Bootstrap Optimism Correction (%d valid samples) ─────────\n",
            length(opt_abe)))
cat(sprintf("Primary model  — apparent AUC: %.3f | optimism: %.4f | corrected: %.3f\n",
            apparent_abe_auc,  mean_opt_abe,  auc_corr_abe))
cat(sprintf("Base model     — apparent AUC: %.3f | optimism: %.4f | corrected: %.3f\n",
            apparent_base_auc, mean_opt_base, auc_corr_base))
cat(sprintf("Incremental \u0394AUC (optimism-corrected): %.3f\n",
            auc_corr_abe - auc_corr_base))

bootstrap_tbl <- tibble(
  Model             = c("Base model (age + NISS + mechanism)",
                        "Primary model (base + ABE)",
                        "Incremental \u0394AUC (ABE contribution)"),
  `Apparent AUC`    = c(round(apparent_base_auc,3), round(apparent_abe_auc,3), "—"),
  `Mean optimism`   = c(round(mean_opt_base,4),     round(mean_opt_abe,4),     "—"),
  `Corrected AUC`   = c(round(auc_corr_base,3),     round(auc_corr_abe,3),
                         round(auc_corr_abe-auc_corr_base,3))
)

# 11. INCREMENTAL VALUE
# ================================================================
m_base_cc <- glm(expire_inhosp~age+niss+mechanism,data=cohort,family=binomial)
m_abe_cc  <- glm(expire_inhosp~alactic_base_excess+age+niss+mechanism,data=cohort,family=binomial)
m_base_30 <- glm(expire_30day ~age+niss+mechanism,data=cohort,family=binomial)
m_abe_30  <- glm(expire_30day ~alactic_base_excess+age+niss+mechanism,data=cohort,family=binomial)

roc_base_m    <- roc(cohort$expire_inhosp,fitted(m_base_cc),ci=TRUE,quiet=TRUE)
roc_abe_m     <- roc(cohort$expire_inhosp,fitted(m_abe_cc), ci=TRUE,quiet=TRUE)
roc_base_m_30 <- roc(cohort$expire_30day, fitted(m_base_30),ci=TRUE,quiet=TRUE)
roc_abe_m_30  <- roc(cohort$expire_30day, fitted(m_abe_30), ci=TRUE,quiet=TRUE)

lrt_incr    <- lrtest(m_base_cc,m_abe_cc)
```

```
lrt_incr_30 <- lrtest(m_base_30,m_abe_30)
delong_incr    <- roc.test(roc_base_m,   roc_abe_m,   method="delong")
delong_incr_30 <- roc.test(roc_base_m_30,roc_abe_m_30,method="delong")

cat("\n── NRI/IDI bootstrap ────────────────────────────────────────\n")
ni_ih <- nri_idi_boot(cohort$expire_inhosp,fitted(m_base_cc),fitted(m_abe_cc),seed=42)
ni_30 <- nri_idi_boot(cohort$expire_30day, fitted(m_base_30),fitted(m_abe_30),seed=43)
cat(sprintf("In-hosp NRI=%.3f (%s) p=%s | IDI=%.3f (%s) p=%s\n",
    ni_ih$nri,ni_ih$nri_ci,ni_ih$nri_p,ni_ih$idi,ni_ih$idi_ci,ni_ih$idi_p))
cat(sprintf("30-day  NRI=%.3f (%s) p=%s | IDI=%.3f (%s) p=%s\n",
    ni_30$nri,ni_30$nri_ci,ni_30$nri_p,ni_30$idi,ni_30$idi_ci,ni_30$idi_p))

fmt_auc_ci <- function(r)
  paste0(round(as.numeric(auc(r)),3),
         " (",round(ci(r)[1],3),"–",round(ci(r)[3],3),")")

incr_tbl <- tibble(
  Metric=c("AUC – Base model (95% CI)","AUC – Base + ABE (95% CI)","ΔAUC",
           "DeLong p","LRT χ²","LRT p",
           "NRI","NRI 95% CI","NRI p",
           "NRI+ (events)","NRI– (non-events)",
           "IDI","IDI 95% CI","IDI p"),
  `In-hospital`=c(
    fmt_auc_ci(roc_base_m),fmt_auc_ci(roc_abe_m),
    round(as.numeric(auc(roc_abe_m))-as.numeric(auc(roc_base_m)),3),
    round(delong_incr$p.value,3),
    round(lrt_incr$Chisq[2],3),round(lrt_incr$`Pr(>Chisq)`[2],3),
    ni_ih$nri,ni_ih$nri_ci,ni_ih$nri_p,
    ni_ih$nri_plus,ni_ih$nri_minus,
    ni_ih$idi,ni_ih$idi_ci,ni_ih$idi_p),
  `30-day`=c(
    fmt_auc_ci(roc_base_m_30),fmt_auc_ci(roc_abe_m_30),
    round(as.numeric(auc(roc_abe_m_30))-as.numeric(auc(roc_base_m_30)),3),
    round(delong_incr_30$p.value,3),
    round(lrt_incr_30$Chisq[2],3),round(lrt_incr_30$`Pr(>Chisq)`[2],3),
    ni_30$nri,ni_30$nri_ci,ni_30$nri_p,
    ni_30$nri_plus,ni_30$nri_minus,
    ni_30$idi,ni_30$idi_ci,ni_30$idi_p)
)

# =================================================================
# 12. TRISS INTEGRATION
# =================================================================
m_triss_only    <- glm(expire_inhosp~triss_mort,data=cohort,family=binomial)

# ── TRISS n-check: how many patients have complete TRISS data ────
n_triss_complete <- sum(!is.na(cohort$triss_mort))
n_triss_missing  <- sum(is.na(cohort$triss_mort))
cat(sprintf("\n── TRISS completeness: %d/%d patients (%.1f%%) have valid TRISS\n",
            n_triss_complete, nrow(cohort),
            n_triss_complete/nrow(cohort)*100))
cat(sprintf("   (%d patients missing TRISS – primarily due to missing GCS)\n",
            n_triss_missing))

m_triss_only_30 <- glm(expire_30day ~triss_mort,data=cohort,family=binomial)
m_triss_abe_30  <- glm(expire_30day ~triss_mort+alactic_base_excess,data=cohort,family=binomial)

roc_triss_m     <- roc(cohort$expire_inhosp,fitted(m_triss_only),    ci=TRUE,quiet=TRUE)
roc_triss_abe_m <- roc(cohort$expire_inhosp,fitted(m_triss_abe_cc),  ci=TRUE,quiet=TRUE)
roc_triss_m_30  <- roc(cohort$expire_30day, fitted(m_triss_only_30), ci=TRUE,quiet=TRUE)
roc_triss_abe_30<- roc(cohort$expire_30day, fitted(m_triss_abe_30),  ci=TRUE,quiet=TRUE)

lrt_triss_ih    <- lrtest(m_triss_only,   m_triss_abe_cc)
lrt_triss_30    <- lrtest(m_triss_only_30,m_triss_abe_30)
delong_triss_ih <- roc.test(roc_triss_m,   roc_triss_abe_m, method="delong")
delong_triss_30 <- roc.test(roc_triss_m_30,roc_triss_abe_30,method="delong")

ni_triss_ih <- nri_idi_boot(cohort$expire_inhosp,
                             fitted(m_triss_only),fitted(m_triss_abe_cc),seed=44)
ni_triss_30 <- nri_idi_boot(cohort$expire_30day,
                             fitted(m_triss_only_30),fitted(m_triss_abe_30),seed=45)

cat("\n── TRISS NRI/IDI ────────────────────────────────────────────\n")
cat(sprintf("In-hosp NRI=%.3f (%s) p=%s | IDI=%.3f (%s) p=%s\n",
    ni_triss_ih$nri,ni_triss_ih$nri_ci,ni_triss_ih$nri_p,
    ni_triss_ih$idi,ni_triss_ih$idi_ci,ni_triss_ih$idi_p))
cat(sprintf("30-day  NRI=%.3f (%s) p=%s | IDI=%.3f (%s) p=%s\n",
```

```r
    ni_triss_30$nri,ni_triss_30$nri_ci,ni_triss_30$nri_p,
    ni_triss_30$idi,ni_triss_30$idi_ci,ni_triss_30$idi_p))

triss_tbl <- tibble(
  Outcome=c("In-hospital","In-hospital","30-day","30-day"),
  Model  =c("TRISS alone","TRISS + ABE","TRISS alone","TRISS + ABE"),
  AUC=c(round(as.numeric(auc(roc_triss_m)),3),
        round(as.numeric(auc(roc_triss_abe_m)),3),
        round(as.numeric(auc(roc_triss_m_30)),3),
        round(as.numeric(auc(roc_triss_abe_30)),3)),
  `95% CI`=c(paste0(round(ci(roc_triss_m)[1],3),   "–",round(ci(roc_triss_m)[3],3)),
             paste0(round(ci(roc_triss_abe_m)[1],3),"–",round(ci(roc_triss_abe_m)[3],3)),
             paste0(round(ci(roc_triss_m_30)[1],3), "–",round(ci(roc_triss_m_30)[3],3)),
             paste0(round(ci(roc_triss_abe_30)[1],3),"–",round(ci(roc_triss_abe_30)[3],3))),
  DeLong_p=c("–",round(delong_triss_ih$p.value,3),"–",round(delong_triss_30$p.value,3)),
  LRT_p   =c("–",round(lrt_triss_ih$`Pr(>Chisq)`[2],3),
             "–",round(lrt_triss_30$`Pr(>Chisq)`[2],3)),
  NRI     =c("–",ni_triss_ih$nri,"–",ni_triss_30$nri),
  NRI_CI  =c("–",ni_triss_ih$nri_ci,"–",ni_triss_30$nri_ci),
  NRI_p   =c("–",ni_triss_ih$nri_p,"–",ni_triss_30$nri_p),
  IDI     =c("–",ni_triss_ih$idi,"–",ni_triss_30$idi),
  IDI_CI  =c("–",ni_triss_ih$idi_ci,"–",ni_triss_30$idi_ci),
  IDI_p   =c("–",ni_triss_ih$idi_p,"–",ni_triss_30$idi_p)
)

# ================================================================
# 12.1 TRISS COMPLETE-CASE SENSITIVITY ANALYSIS (OBSERVED GCS ONLY)
# ================================================================
# GCS is missing in 47% of patients
# (predominantly due to intubation at admission), which is missing-not-
# at-random. The primary TRISS analysis uses multiply-imputed GCS for
# these patients. This sensitivity analysis restricts to patients with
# OBSERVED (non-imputed) GCS only, to check whether the TRISS-integration
# findings are robust to this missingness mechanism. triss_mort for these
# patients already reflects their true observed GCS (imputation only
# fills in what is missing elsewhere), so no recomputation is needed –
# only restriction of the analysis sample.

cohort_gcs_cc <- cohort %>% filter(!is.na(gcs_total))
n_gcs_cc <- nrow(cohort_gcs_cc)
n_gcs_cc_deaths <- sum(cohort_gcs_cc$expire_inhosp)
cat(sprintf("TRISS complete-case (observed GCS only): n=%d of %d (%.1f%%); %d deaths (%.1f%%)\n",
            n_gcs_cc, nrow(cohort), n_gcs_cc/nrow(cohort)*100,
            n_gcs_cc_deaths, n_gcs_cc_deaths/n_gcs_cc*100))

m_triss_only_cc2 <- glm(expire_inhosp~triss_mort, data=cohort_gcs_cc, family=binomial)
m_triss_abe_cc2  <- glm(expire_inhosp~triss_mort+alactic_base_excess, data=cohort_gcs_cc, family=binomial)

roc_triss_cc2     <- roc(cohort_gcs_cc$expire_inhosp, fitted(m_triss_only_cc2), ci=TRUE, quiet=TRUE)
roc_triss_abe_cc2 <- roc(cohort_gcs_cc$expire_inhosp, fitted(m_triss_abe_cc2),  ci=TRUE, quiet=TRUE)

lrt_triss_cc2    <- lrtest(m_triss_only_cc2, m_triss_abe_cc2)
delong_triss_cc2 <- roc.test(roc_triss_cc2, roc_triss_abe_cc2, method="delong")
ni_triss_cc2     <- nri_idi_boot(cohort_gcs_cc$expire_inhosp,
                                 fitted(m_triss_only_cc2), fitted(m_triss_abe_cc2), seed=46)

cat(sprintf("TRISS alone:  AUC=%.3f (%.3f\u2013%.3f)\n",
            as.numeric(auc(roc_triss_cc2)), ci(roc_triss_cc2)[1], ci(roc_triss_cc2)[3]))
cat(sprintf("TRISS + ABE:  AUC=%.3f (%.3f\u2013%.3f)\n",
            as.numeric(auc(roc_triss_abe_cc2)), ci(roc_triss_abe_cc2)[1], ci(roc_triss_abe_cc2)[3]))
cat(sprintf("DeLong p=%s | LRT p=%s | NRI=%.3f (%s) p=%s | IDI=%.3f (%s) p=%s\n",
            fmt_p(delong_triss_cc2$p.value), fmt_p(lrt_triss_cc2$`Pr(>Chisq)`[2]),
            ni_triss_cc2$nri, ni_triss_cc2$nri_ci, ni_triss_cc2$nri_p,
            ni_triss_cc2$idi, ni_triss_cc2$idi_ci, ni_triss_cc2$idi_p))

triss_cc_tbl <- tibble(
  Model = c("TRISS alone", "TRISS + ABE"),
  `n (complete-case)` = rep(n_gcs_cc, 2),
  AUC = c(round(as.numeric(auc(roc_triss_cc2)),3), round(as.numeric(auc(roc_triss_abe_cc2)),3)),
  `95% CI` = c(paste0(round(ci(roc_triss_cc2)[1],3),"\u2013",round(ci(roc_triss_cc2)[3],3)),
               paste0(round(ci(roc_triss_abe_cc2)[1],3),"\u2013",round(ci(roc_triss_abe_cc2)[3],3))),
  DeLong_p = c("\u2014", fmt_p(delong_triss_cc2$p.value)),
  LRT_p    = c("\u2014", fmt_p(lrt_triss_cc2$`Pr(>Chisq)`[2])),
  NRI      = c("\u2014", ni_triss_cc2$nri),
  NRI_p    = c("\u2014", ni_triss_cc2$nri_p),
  IDI      = c("\u2014", ni_triss_cc2$idi),
  IDI_p    = c("\u2014", ni_triss_cc2$idi_p)
```

```r
)
print(as.data.frame(triss_cc_tbl))




# ==================================================================
# 12.5 DECISION CURVE ANALYSIS
# ==================================================================
# Assesses whether ABE changes clinical decisions
# (net benefit) across plausible risk thresholds, rather than relying
# solely on AUC/NRI/IDI. Two comparisons: base model vs. base+ABE, and
# TRISS vs. TRISS+ABE.
if (!require("dcurves")) install.packages("dcurves", repos="https://cloud.r-project.org")
library(dcurves)
library(patchwork)  # for side-by-side panels; install if needed

cohort_dca <- cohort %>%
  mutate(
    pred_base       = predict(m_base_cc, type = "response"),
    pred_abe        = predict(m_abe_cc, type = "response"),
    pred_triss      = predict(m_triss_only, type = "response"),
    pred_triss_abe  = predict(m_triss_abe_cc, type = "response")
  )

# ── Panel (a): base model vs. base + ABE ──────────────────────
dca_base_fit <- dca(
  expire_inhosp ~ pred_base + pred_abe,
  data = cohort_dca,
  label = list(pred_base = "Base model", pred_abe = "Base + ABE")
)
panel_a <- plot(dca_base_fit, smooth = TRUE) +
  FIG_THEME +
  labs(title = NULL) +
  theme(legend.position = c(0.7, 0.75))

# ── Panel (b): TRISS vs. TRISS + ABE ───────────────────────────
dca_triss_fit <- dca(
  expire_inhosp ~ pred_triss + pred_triss_abe,
  data = cohort_dca,
  label = list(pred_triss = "TRISS", pred_triss_abe = "TRISS + ABE")
)
panel_b <- plot(dca_triss_fit, smooth = TRUE) +
  FIG_THEME +
  labs(title = NULL) +
  theme(legend.position = c(0.7, 0.75))

fig_dca <- panel_a + panel_b +
  plot_annotation(tag_levels = "a", tag_prefix = "(", tag_suffix = ")")

save_fig(fig_dca, "FigureS4", "FigureS4", width = FW_SINGLE * 1.7, height = FH_STD)

# ── Report net benefit at a few clinically anchored thresholds ──
# 15% ≈ near the Youden-derived risk cut; 29% ≈ observed cohort prevalence
report_thresholds <- c(0.10, 0.15, 0.20, 0.29, 0.35)

nb_base  <- as_tibble(dca_base_fit) %>%
  filter(round(threshold, 2) %in% report_thresholds) %>%
  select(threshold, label, net_benefit)
nb_triss <- as_tibble(dca_triss_fit) %>%
  filter(round(threshold, 2) %in% report_thresholds) %>%
  select(threshold, label, net_benefit)

cat("\n── Decision Curve Analysis: Net Benefit at Selected Thresholds ──\n")
print(nb_base)
print(nb_triss)

# ==================================================================
# 13. SENSITIVITY ANALYSES
# ==================================================================
cat("\n── Sensitivity 1: 60-min ABG ───────────────────────────────\n")
roc_1hr    <- roc(cohort_1hr$expire_inhosp,cohort_1hr$alactic_base_excess,
                  ci=TRUE,quiet=TRUE)
youden_1hr <- coords(roc_1hr,"best",best.method="youden",
                     ret=c("threshold","sensitivity","specificity","ppv","npv"))

m_firth_1hr <- logistf(expire_inhosp~alactic_base_excess+age+niss+mechanism,
```

```
                        data=cohort_1hr,pl=TRUE,
                        control=logistf.control(maxit=200))

abe_coef_firth <- if (!is.na(m_firth_1hr$coefficients["alactic_base_excess"]) &&
                       abs(m_firth_1hr$coefficients["alactic_base_excess"]) > 0.001) {
  list(or=exp(m_firth_1hr$coefficients["alactic_base_excess"]),
       lo=exp(m_firth_1hr$ci.lower["alactic_base_excess"]),
       hi=exp(m_firth_1hr$ci.upper["alactic_base_excess"]),
       p=m_firth_1hr$prob["alactic_base_excess"],
       method="Firth")
} else NULL

m_1hr_glm <- suppressWarnings(
  glm(expire_inhosp~alactic_base_excess+age+niss+mechanism,
      data=cohort_1hr,family=binomial,
      control=glm.control(maxit=200,epsilon=1e-10)))
s_glm <- coef(summary(m_1hr_glm))
abe_coef_glm <- if ("alactic_base_excess" %in% rownames(s_glm) &&
                     !is.na(s_glm["alactic_base_excess","Estimate"]) &&
                     abs(s_glm["alactic_base_excess","Estimate"]) > 0.001) {
  list(or=exp(s_glm["alactic_base_excess","Estimate"]),
       lo=exp(s_glm["alactic_base_excess","Estimate"]-1.96*s_glm["alactic_base_excess","Std. Error"]),
       hi=exp(s_glm["alactic_base_excess","Estimate"]+1.96*s_glm["alactic_base_excess","Std. Error"]),
       p=s_glm["alactic_base_excess","Pr(>|z|)"],
       method="GLM")
} else NULL

model_1hr <- if (!is.null(abe_coef_firth)) abe_coef_firth else
             if (!is.null(abe_coef_glm))   abe_coef_glm   else {
               warning("Both models degenerate.")
               list(or=NA,lo=NA,hi=NA,p=NA,method="FAILED")
             }

or_1hr    <- round(model_1hr$or,3)
ci_1hr_lo <- round(model_1hr$lo,3)
ci_1hr_hi <- round(model_1hr$hi,3)
p_1hr     <- round(model_1hr$p,3)
cat(sprintf("Selected: %s | OR=%.3f (%.3f–%.3f) p=%.4f\n",
            model_1hr$method,or_1hr,ci_1hr_lo,ci_1hr_hi,p_1hr))
cat(sprintf("AUC=%.3f (%.3f–%.3f) | Threshold=%.2f | Sens %.1f%% | NPV %.1f%%\n",
    as.numeric(auc(roc_1hr)),ci(roc_1hr)[1],ci(roc_1hr)[3],
    youden_1hr$threshold,youden_1hr$sensitivity*100,youden_1hr$npv*100))

cat("\n── Sensitivity 2: Sepsis excluded ─────────────────────────\n")
roc_nosep <- roc(cohort_nosep$expire_inhosp,cohort_nosep$alactic_base_excess,
                 ci=TRUE,quiet=TRUE)
m_nosep   <- suppressWarnings(
  glm(expire_inhosp~alactic_base_excess+age+niss+mechanism,
      data=cohort_nosep,family=binomial))
s_nosep     <- coef(summary(m_nosep))
or_nosep    <- round(exp(s_nosep["alactic_base_excess","Estimate"]),3)
se_nosep    <- s_nosep["alactic_base_excess","Std. Error"]
ci_nosep_lo <- round(exp(s_nosep["alactic_base_excess","Estimate"]-1.96*se_nosep),3)
ci_nosep_hi <- round(exp(s_nosep["alactic_base_excess","Estimate"]+1.96*se_nosep),3)
p_nosep     <- round(s_nosep["alactic_base_excess","Pr(>|z|)"],3)
cat(sprintf("OR=%.3f (%.3f–%.3f) p=%.3f | AUC=%.3f\n",
    or_nosep,ci_nosep_lo,ci_nosep_hi,p_nosep,as.numeric(auc(roc_nosep))))

sens_tbl <- tibble(
  Analysis     =c("60-min ABG window†","Sepsis excluded"),
  n            =c(nrow(cohort_1hr),nrow(cohort_nosep)),
  Deaths       =c(sum(cohort_1hr$expire_inhosp),sum(cohort_nosep$expire_inhosp)),
  Mortality_pct=round(c(mean(cohort_1hr$expire_inhosp),
                         mean(cohort_nosep$expire_inhosp))*100,1),
  `AUC (95% CI)`=c(
    paste0(round(as.numeric(auc(roc_1hr)),3),
           " (",round(ci(roc_1hr)[1],3),"–",round(ci(roc_1hr)[3],3),")"),
    paste0(round(as.numeric(auc(roc_nosep)),3),
           " (",round(ci(roc_nosep)[1],3),"–",round(ci(roc_nosep)[3],3),")")),
  Threshold    =c(round(youden_1hr$threshold,2),round(youden$threshold,2)),
  NPV_pct      =round(c(youden_1hr$npv*100,NA),1),
  `OR (95% CI)`=c(
    sprintf("%.3f (%.3f–%.3f)",or_1hr,  ci_1hr_lo,  ci_1hr_hi),
    sprintf("%.3f (%.3f–%.3f)",or_nosep,ci_nosep_lo,ci_nosep_hi)),
  p_ABE=c(p_1hr,p_nosep)
)
```

```r
# ================================================================
# 13.1 SENSITIVITY ANALYSIS: ALCOHOL-RELATED CODES EXCLUDED
# ================================================================
# Acute alcohol intoxication (F10.0),
# alcohol abuse (F10.1), and toxic effects of alcohol (T51.x) were not
# applied as exclusion criteria in the primary cohort. This sensitivity
# analysis excludes patients with any such code and re-fits the primary
# model, to bound the potential influence of ethanol-related lactate
# elevation on ABE. Mirrors the sepsis_flag construction in Section 2.

alc_cols <- grep("^dx", names(pts_120m_raw), value = TRUE, ignore.case = TRUE)
alc_vec <- if (length(alc_cols) > 0) {
  as.integer(rowSums(sapply(alc_cols, function(col) {
    vals <- pts_120m_raw[[col]]
    !is.na(vals) & grepl("F100|F101|T51", vals, ignore.case = TRUE)
  })) > 0)
} else rep(0L, nrow(pts_120m_raw))

pts_120m_alc <- pts_120m_raw %>%
  mutate(alcohol_flag = alc_vec) %>%
  select(subject_id, hadm_id, alcohol_flag)

cohort <- cohort %>% left_join(pts_120m_alc, by = c("subject_id","hadm_id"))
cat(sprintf("Alcohol-related codes present: %d of %d admissions\n",
            sum(cohort$alcohol_flag, na.rm=TRUE), nrow(cohort)))

cohort_noalc <- cohort %>% filter(!is.na(alcohol_flag) & alcohol_flag == 0)

roc_noalc <- roc(cohort_noalc$expire_inhosp, cohort_noalc$alactic_base_excess, ci=TRUE, quiet=TRUE)
m_noalc   <- suppressWarnings(
  glm(expire_inhosp~alactic_base_excess+age+niss+mechanism,
      data=cohort_noalc, family=binomial))
s_noalc     <- coef(summary(m_noalc))
or_noalc    <- round(exp(s_noalc["alactic_base_excess","Estimate"]),3)
se_noalc    <- s_noalc["alactic_base_excess","Std. Error"]
ci_noalc_lo <- round(exp(s_noalc["alactic_base_excess","Estimate"]-1.96*se_noalc),3)
ci_noalc_hi <- round(exp(s_noalc["alactic_base_excess","Estimate"]+1.96*se_noalc),3)
p_noalc     <- round(s_noalc["alactic_base_excess","Pr(>|z|)"],3)

cat(sprintf("Alcohol-excluded: n=%d | OR=%.3f (%.3f\u2013%.3f) p=%.3f | AUC=%.3f\n",
    nrow(cohort_noalc), or_noalc, ci_noalc_lo, ci_noalc_hi, p_noalc,
    as.numeric(auc(roc_noalc))))

# ── Extend the existing sensitivity-analyses table with this third row ──
# (Assumes sens_tbl was already built in Section 13 with 60-min and
# sepsis-excluded rows; re-run that tibble() call with this addition.)
sens_tbl <- tibble(
  Analysis     = c("60-min ABG window\u2020","Sepsis excluded","Alcohol-related codes excluded"),
  n            = c(nrow(cohort_1hr), nrow(cohort_nosep), nrow(cohort_noalc)),
  Deaths       = c(sum(cohort_1hr$expire_inhosp), sum(cohort_nosep$expire_inhosp), sum(cohort_noalc$expire_inhosp)),
  Mortality_pct= round(c(mean(cohort_1hr$expire_inhosp), mean(cohort_nosep$expire_inhosp),
                         mean(cohort_noalc$expire_inhosp))*100, 1),
  `AUC (95% CI)` = c(
    paste0(round(as.numeric(auc(roc_1hr)),3)," (",round(ci(roc_1hr)[1],3),"\u2013",round(ci(roc_1hr)[3],3),")"),
    paste0(round(as.numeric(auc(roc_nosep)),3)," (",round(ci(roc_nosep)[1],3),"\u2013",round(ci(roc_nosep)[3],3),")"),
    paste0(round(as.numeric(auc(roc_noalc)),3)," (",round(ci(roc_noalc)[1],3),"\u2013",round(ci(roc_noalc)[3],3),")")),
  Threshold = c(round(youden_1hr$threshold,2), round(youden$threshold,2), NA),
  NPV_pct   = round(c(youden_1hr$npv*100, NA, NA), 1),
  `OR (95% CI)` = c(
    sprintf("%.3f (%.3f\u2013%.3f)", or_1hr, ci_1hr_lo, ci_1hr_hi),
    sprintf("%.3f (%.3f\u2013%.3f)", or_nosep, ci_nosep_lo, ci_nosep_hi),
    sprintf("%.3f (%.3f\u2013%.3f)", or_noalc, ci_noalc_lo, ci_noalc_hi)),
  p_ABE = c(p_1hr, p_nosep, p_noalc)
)
print(as.data.frame(sens_tbl))

# ================================================================
# 13.2 SENSITIVITY ANALYSIS: ADJUSTED FOR ADMISSION CREATININE (AKI)
# ================================================================
# Acute kidney injury was not excluded
# and may confound ABE via renal (rather than perfusion-related)
# acid-base derangement. This sensitivity model adds admission
# creatinine as a covariate directly, rather than excluding AKI
```

```
# patients (deliberately retained as a direct consequence of
# haemorrhagic shock, per the Discussion).

n_creat_missing <- sum(is.na(cohort$creatinine))
cat(sprintf("Creatinine missing: %d of %d patients (%.1f%%)\n",
            n_creat_missing, nrow(cohort), n_creat_missing/nrow(cohort)*100))

m_creat_cc <- glm(expire_inhosp~alactic_base_excess+age+niss+mechanism+creatinine,
                   data=cohort, family=binomial)
s_creat    <- coef(summary(m_creat_cc))
n_creat_cc <- nobs(m_creat_cc)

or_creat_abe    <- round(exp(s_creat["alactic_base_excess","Estimate"]),3)
se_creat_abe    <- s_creat["alactic_base_excess","Std. Error"]
ci_creat_abe_lo <- round(exp(s_creat["alactic_base_excess","Estimate"]-1.96*se_creat_abe),3)
ci_creat_abe_hi <- round(exp(s_creat["alactic_base_excess","Estimate"]+1.96*se_creat_abe),3)
p_creat_abe     <- round(s_creat["alactic_base_excess","Pr(>|z|)"],3)

or_creat_cr    <- round(exp(s_creat["creatinine","Estimate"]),3)
se_creat_cr    <- s_creat["creatinine","Std. Error"]
ci_creat_cr_lo <- round(exp(s_creat["creatinine","Estimate"]-1.96*se_creat_cr),3)
ci_creat_cr_hi <- round(exp(s_creat["creatinine","Estimate"]+1.96*se_creat_cr),3)
p_creat_cr     <- round(s_creat["creatinine","Pr(>|z|)"],3)

cat(sprintf("Creatinine-adjusted (n=%d complete case): ABE OR=%.3f (%.3f\u2013%.3f) p=%.3f | Creatinine OR=%.3f (%.3f\u2013%.3f) p=%.3f\n",
            n_creat_cc, or_creat_abe, ci_creat_abe_lo, ci_creat_abe_hi, p_creat_abe,
            or_creat_cr, ci_creat_cr_lo, ci_creat_cr_hi, p_creat_cr))

creat_tbl <- tibble(
  Variable = c("ABE (per 1 mmol/L)", "Creatinine (per 1 mg/dL)"),
  `n (complete case)` = rep(n_creat_cc, 2),
  OR = c(or_creat_abe, or_creat_cr),
  `95% CI` = c(sprintf("%.3f\u2013%.3f", ci_creat_abe_lo, ci_creat_abe_hi),
               sprintf("%.3f\u2013%.3f", ci_creat_cr_lo, ci_creat_cr_hi)),
  `p-value` = c(fmt_p(p_creat_abe), fmt_p(p_creat_cr))
)
print(as.data.frame(creat_tbl))




# ================================================================
# 5.1 THRESHOLD METRIC CONFIDENCE INTERVALS (BOOTSTRAP)
# ================================================================
# The Youden threshold is derived and
# evaluated on the same data, and PPV/NPV are prevalence-dependent.
# This adds bootstrap 95% CIs for sensitivity, specificity, PPV and NPV
# at the Youden-optimal threshold, for both the primary and 60-minute
# cohorts. Both sets of thresholds remain exploratory (derived and
# evaluated on the same data) regardless of the CIs reported here.

fmt_ci_coord <- function(ci_obj, metric) {
  v <- ci_obj[[metric]][1, ]
  sprintf("%.1f%% (%.1f\u2013%.1f%%)", v["50%"]*100, v["2.5%"]*100, v["97.5%"]*100)
}

# Passing the already-computed threshold directly (rather than x="best")
# avoids ci.coords() re-deriving "best" on its own and failing if the
# bootstrap ever produces tied-best thresholds in a replicate.
set.seed(42)
ci_youden <- ci.coords(roc_abe, x=youden$threshold, input="threshold",
                        ret=c("sensitivity","specificity","ppv","npv"), boot.n=2000)
set.seed(42)
ci_youden_1hr <- ci.coords(roc_1hr, x=youden_1hr$threshold, input="threshold",
                            ret=c("sensitivity","specificity","ppv","npv"), boot.n=2000)

# Display the already-computed point estimate (matching the manuscript's
# existing text exactly) and take only the CI bounds from the bootstrap.
fmt_ci_coord <- function(point_pct, ci_obj, metric) {
  v <- ci_obj[[metric]][1, ]
  sprintf("%.1f%% (%.1f\u2013%.1f%%)", point_pct, v["2.5%"]*100, v["97.5%"]*100)
}

threshold_ci_tbl <- tibble(
  Cohort = c("Primary (\u22642 h, n=134)", "60-minute (n=54)"),
  Threshold = c(round(youden$threshold,2), round(youden_1hr$threshold,2)),
```

```r
  `Sensitivity (95% CI)` = c(fmt_ci_coord(youden$sensitivity*100, ci_youden, "sensitivity"),
                             fmt_ci_coord(youden_1hr$sensitivity*100, ci_youden_1hr, "sensitivity")),
  `Specificity (95% CI)` = c(fmt_ci_coord(youden$specificity*100, ci_youden, "specificity"),
                             fmt_ci_coord(youden_1hr$specificity*100, ci_youden_1hr, "specificity")),
  `PPV (95% CI)` = c(fmt_ci_coord(youden$ppv*100, ci_youden, "ppv"),
                     fmt_ci_coord(youden_1hr$ppv*100, ci_youden_1hr, "ppv")),
  `NPV (95% CI)` = c(fmt_ci_coord(youden$npv*100, ci_youden, "npv"),
                     fmt_ci_coord(youden_1hr$npv*100, ci_youden_1hr, "npv"))
)
cat("Threshold-derived metrics with bootstrap 95% CIs (both exploratory \u2014\n")
cat("derived and evaluated on the same data):\n")
print(as.data.frame(threshold_ci_tbl))


# ==================================================================
# 14. FIGURES — PUBLICATION STYLE
# ==================================================================
# Figure requirements:
#   - No titles or captions inside the figure (add in manuscript text)
#   - Font: Helvetica / Arial ("sans" on Windows)
#   - Colours: FIG_NAVY, FIG_BLUE, FIG_MAUVE, FIG_LIGHT, FIG_AMBER
#   - Single-column width: 174 mm (6.85 in); double-column: 84 mm (3.31 in)
#   - Save as PDF for print/submission + PNG for Word document
#   - File names: Fig1.pdf (main); FigureS1.pdf, FigureS2.pdf, FigureS3.pdf, FigureS4.pdf (supplementary)
# ==================================================================
lbl_ci <- function(r,nm)
  sprintf("%s  %.3f (%.3f–%.3f)",nm,as.numeric(auc(r)),ci(r)[1],ci(r)[3])

# ── Fig 1 (MAIN MANUSCRIPT) — Individual markers ROC ──────────
# single-column (174 mm wide), no title, Helvetica, six-colour palette
fig1 <- ggroc(
  setNames(roc_list, mapply(lbl_ci, roc_list, names(roc_list))),
  legacy.axes=TRUE, linewidth=0.7) +
  geom_abline(slope=1,intercept=0,linetype="dashed",color=FIG_GRAY,linewidth=0.5) +
  scale_color_manual(values=FIG_PALETTE6) +
  labs(x="1 \u2212 Specificity", y="Sensitivity", color=NULL) +
  FIG_THEME +
  theme(legend.position=c(0.70,0.26))

save_fig(fig1, "Fig1", "Fig1", width=FW_SINGLE, height=FH_STD)

# ── Figure S3 — Sensitivity cohort comparison ROC ────────────
fig4 <- ggroc(
  setNames(list(roc_abe, roc_1hr),
           c(lbl_ci(roc_abe,"Primary cohort (2 h)"),
             lbl_ci(roc_1hr,"60-min cohort"))),
  legacy.axes=TRUE, linewidth=0.7) +
  geom_abline(slope=1,intercept=0,linetype="dashed",colour=FIG_GRAY,linewidth=0.5) +
  scale_color_manual(values=c(FIG_GRAY, FIG_AMBER)) +
  labs(x="1 \u2212 Specificity", y="Sensitivity", color=NULL) +
  FIG_THEME +
  theme(legend.position=c(0.58,0.12))

save_fig(fig4, "FigureS3", "FigureS3", width=FW_SINGLE, height=FH_STD)

cat(sprintf("\nFigures saved:\n  Publication (PDF):  %s\n  Word (PNG): %s\n",
            fig_dir_pdf, fig_dir_word))

# ── Figure S1 — Calibration plot (primary model) ─────────────
# Smoothed calibration curve (LOESS) with rug plot.
# Annotated with key calibration metrics.
fig_cal <- ggplot(data.frame(pred=pred_m2, obs=as.factor(obs_m2)),
                  aes(x=pred)) +
  geom_rug(aes(colour=obs), alpha=0.40, sides="b",
           length=unit(0.025, "npc")) +
  geom_smooth(aes(y=as.integer(as.character(obs))),
              method="loess", span=0.75, se=TRUE,
              colour=FIG_BLUE, fill=FIG_LIGHT,
              linewidth=0.7, alpha=0.25) +
  geom_abline(slope=1, intercept=0, linetype="dashed",
              colour=FIG_GRAY, linewidth=0.5) +
  scale_colour_manual(values=c("0"=FIG_MAUVE,"1"=FIG_NAVY), guide="none") +
  scale_x_continuous(limits=c(0,1), breaks=seq(0,1,0.2),
                     labels=scales::percent_format(accuracy=1)) +
  scale_y_continuous(limits=c(0,1), breaks=seq(0,1,0.2),
                     labels=scales::percent_format(accuracy=1)) +
  labs(x="Predicted probability of in-hospital mortality",
```

```r
       y="Observed event proportion") +
  annotate("text", x=0.62, y=0.10, hjust=0, size=2.5,
           colour="grey30", family="sans",
           label=sprintf("Brier = %.3f\nCal. slope = %.3f\nCITL = %.4f",
                          brier_score, cal_slope, citl_val)) +
  FIG_THEME

save_fig(fig_cal, "FigureS1", "FigureS1", width=FW_SINGLE, height=FH_STD)

cat("  Main manuscript: Fig1.pdf\n")
cat("  Supplementary:   FigureS1.pdf (calibration plot), FigureS2.pdf (flow diagram),\n")
cat("                   FigureS3.pdf (sensitivity cohort), FigureS4.pdf (decision curve analysis)\n")

# ==================================================================
# 15. BUILD WORD DOCUMENT
# ==================================================================
doc <- read_docx()
ft_s <- function(df) {
  flextable(as.data.frame(df)) %>%
    theme_booktabs() %>% fontsize(size=10,part="all") %>%
    font(fontname="Times New Roman",part="all") %>%
    set_table_properties(width=1,layout="autofit") %>% bold(part="header")
}
h1      <- function(doc,t) body_add_par(doc,t,style="heading 1")
h2      <- function(doc,t) body_add_par(doc,t,style="heading 2")
add_tbl <- function(doc,df,cap=NULL) {
  if (!is.null(cap)) doc <- h2(doc,cap)
  doc <- body_add_flextable(doc,ft_s(df))
  body_add_par(doc,"",style="Normal")
}
note <- function(doc,t) body_add_par(doc,t,style="Normal")

tbl1_ft <- as_flex_table(tbl1) %>%
  fontsize(size=10,part="all") %>%
  font(fontname="Times New Roman",part="all") %>%
  set_table_properties(width=1,layout="autofit")
cat("Table 1 columns:",paste(tbl1_ft$col_keys,collapse=", "),"\n")

doc <- doc %>%
  body_add_par("Alactic Base Excess – Statistical Analysis",style="heading 1") %>%
  body_add_par(paste("Generated:",format(Sys.time(),"%d %B %Y %H:%M")),style="Normal") %>%
  body_add_par(sprintf("Cohort: n=%d | In-hospital: %d (%.1f%%) | 30-day: %d (%.1f%%)",
               nrow(cohort),sum(cohort$expire_inhosp),mean(cohort$expire_inhosp)*100,
               sum(cohort$expire_30day),mean(cohort$expire_30day)*100),style="Normal") %>%
  body_add_par("",style="Normal") %>%

  h1("1. Baseline Characteristics") %>%
  body_add_flextable(tbl1_ft) %>%
  body_add_par("",style="Normal") %>%
  add_tbl(mortality_tbl,"Mortality Outcomes") %>%
  note("p-values: Kruskal-Wallis (continuous); chi-squared (categorical).") %>%
  body_add_par("",style="Normal") %>%

  h1("2. ABE Distribution") %>%
  add_tbl(abe_dist_tbl,"ABE Summary Statistics (n=134)") %>%
  add_tbl(smd_tbl,"Standardised Mean Differences and p-values by Outcome") %>%
  add_tbl(threshold_tbl,"Youden Threshold Analysis") %>%
  body_add_par("",style="Normal") %>%

  h1("3. ROC – Individual Markers") %>%
  add_tbl(auc_tbl,"AUC (In-Hospital Mortality)") %>%
  add_tbl(delong_tbl,"DeLong Pairwise Comparisons vs ABE") %>%

  h1("4. Univariable Logistic Regression (Pooled MI, n=134)") %>%
  add_tbl(univ_tbl) %>%

  h1("5. Multivariable Logistic Regression") %>%
  add_tbl(multiv_tbl,"All Three Models (Pooled MI)") %>%
  note("Primary model: Model 2 (NISS). Reference category: Blunt mechanism.") %>%
  body_add_par("",style="Normal") %>%
  add_tbl(primary_tbl_ih,"Table 2a. Primary Model – In-Hospital Mortality") %>%
  note("Pooled from 20 imputed datasets (Rubin's rules).") %>%
  body_add_par("",style="Normal") %>%
  add_tbl(primary_tbl_30,"Table 2b. Primary Model – 30-Day Mortality") %>%
  note("Pooled from 20 imputed datasets (Rubin's rules).") %>%
  body_add_par("",style="Normal") %>%
```

```r
  h1("6. Hosmer-Lemeshow Calibration (Complete Case, n=134)") %>%
  add_tbl(hl_tbl) %>%
  note("p > 0.05 = adequate calibration.") %>%
  body_add_par("",style="Normal") %>%

  h1("7. Incremental Value of ABE") %>%
  add_tbl(incr_tbl) %>%
  note("NRI/IDI: 500-iteration bootstrap; p-values test H0=0. Base: age+NISS+mechanism.") %>%
  body_add_par("",style="Normal") %>%

  h1("8. TRISS Integration") %>%
  add_tbl(triss_tbl,"TRISS Alone vs TRISS + ABE (both outcomes)") %>%
  note("NRI and IDI: 500-iteration bootstrap; p-values test H0=0.") %>%
  body_add_par("",style="Normal") %>%

  h1("9. Sensitivity Analyses") %>%
  add_tbl(sens_tbl) %>%
  note(paste0("† 60-min: Firth penalized logistic regression (pl=TRUE). ",
            "7 ABE and 51 lactate values excluded as date-format artefacts.")) %>%
  note(sprintf("60-min Youden: %.2f mmol/L | Sens %.1f%% | Spec %.1f%% | NPV %.1f%%",
    youden_1hr$threshold,youden_1hr$sensitivity*100,
    youden_1hr$specificity*100,youden_1hr$npv*100)) %>%
  body_add_par("",style="Normal") %>%

  h1("10. Primary Model — Formula, Coefficients and Worked Examples") %>%
  note(sprintf(paste0("Formula: L = %.4f + (%.4f\u00d7ABE)",
                    " + (%.4f\u00d7Age) + (%.4f\u00d7NISS)",
                    " + (%.4f\u00d7Penetrating) + (%.4f\u00d7Other)"),
             b0, b_abe, b_age, b_niss, b_pen, b_other)) %>%
  note("P(death) = 1 / (1 + e^(-L))") %>%
  body_add_par("",style="Normal") %>%
  add_tbl(coef_tbl,"10.1 Raw Coefficient Table (β and OR)") %>%
  note(sprintf("Intercept β₀ = %.4f (SE %.4f); 95%% CI: %.4f to %.4f",
             b0,se_b0,b0-1.96*se_b0,b0+1.96*se_b0)) %>%
  body_add_par("",style="Normal") %>%
  add_tbl(example_tbl,"10.2 Worked Examples") %>%
  body_add_par("",style="Normal") %>%


  h1("11. Extended Calibration (Primary Model)") %>%
  add_tbl(cal_metrics_tbl,"Calibration Metrics: Primary Model (n=134, complete case)") %>%
  note(paste0("Brier score: mean squared error; ideal <0.25. ",
            "Scaled Brier: skill vs. null model; ideal >0. ",
            "CITL (calibration-in-the-large): systematic over/under-prediction; ideal 0. ",
            "Calibration slope: spread of predictions; ideal 1; <1 indicates overfitting.")) %>%
  body_add_par("",style="Normal") %>%

  h1("12. Bootstrap Optimism Correction (500 iterations)") %>%
  add_tbl(bootstrap_tbl,
    paste0("Bootstrap Optimism-Corrected AUC (n=",n_boot_cc," complete cases, 500 bootstrap samples)")) %>%
  note(paste0("Harrell's bootstrap optimism correction. ",
            "Apparent AUC reflects development-data performance. ",
            "Corrected AUC adjusts for expected overfitting. ",
            "All primary predictors complete; bootstrap performed on complete-case data.")) %>%
  body_add_par("",style="Normal") %>%

  h1("13. N-Verification Summary") %>%
  note(sprintf("TRISS: %d of %d patients (%.1f%%) have complete TRISS data. Missing patients lacked GCS at admission (intubated).",
             n_triss_complete, nrow(cohort), n_triss_complete/nrow(cohort)*100)) %>%
  note(sprintf("60-min cohort: %d total; %d valid ABE; %d valid lactate; %d complete cases (effective analytic n).",
             n_total_60m, n_valid_abe_60m, n_valid_lac_60m, n_complete_60m)) %>%
  body_add_par("",style="Normal") %>%

  h1("14. Table S2: Excluded-Population Characteristics") %>%
  body_add_flextable(ft_s2) %>%
  note("Characteristics of patients excluded for missing an early arterial blood gas (Query 10, File S2), compared with the final study cohort (n=134). Also exported standalone as TableS2_Excluded_Characteristics.docx.") %>%
  body_add_par("",style="Normal") %>%

  h1("15. Figures (PNG — for Word; PDF in Figures_PDF folder for print/submission)") %>%
  note(paste0("Publication-quality figure files: Fig1.pdf (main manuscript), ",
            "FigureS1.pdf to FigureS4.pdf (Figures S1-S4)")) %>%
  body_add_par("",style="Normal") %>%

```

```r
  h1("16. TRISS Complete-Case Sensitivity Analysis (Observed GCS Only)") %>%
  add_tbl(triss_cc_tbl) %>%
  note(sprintf("Restricted to %d of %d patients (%.1f%%) with observed (non-imputed) GCS, given GCS missingness is not at random (predominantly intubation).",
               n_gcs_cc, nrow(cohort), n_gcs_cc/nrow(cohort)*100)) %>%
  body_add_par("",style="Normal") %>%

    h1("17. Threshold-Derived Metrics with Bootstrap 95% CIs") %>%
  add_tbl(threshold_ci_tbl) %>%
  note("Thresholds derived via the Youden index and evaluated on the same data; exploratory pending external validation.") %>%
  body_add_par("",style="Normal") %>%

  h1("18. Creatinine-Adjusted Sensitivity Model") %>%
  add_tbl(creat_tbl) %>%
  note("ABE association with mortality adjusted for admission creatinine (AKI), rather than excluding AKI patients.") %>%
  body_add_par("",style="Normal")


# Figure captions (captions in manuscript text, not in figure)
fig_captions <- list(
  "Fig1"     = "Fig. 1 ROC curves for individual markers against in-hospital mortality. AUC values are shown with 95% CIs (DeLong method). ABE alactic base excess, AUC area under the receiver operating characteristic curve, BE base excess, CI confidence interval, ISS Injury Severity Score, NISS New Injury Severity Score, TRISS Trauma and Injury Severity Score",
  "FigureS1" = "Figure S1. Calibration plot for the primary multivariable model (ABE + age + NISS + mechanism), in-hospital mortality, complete-case analysis (n=134). Smoothed calibration curve derived by LOESS regression. Dashed diagonal = perfect calibration. Rug marks indicate individual patients (dark = died, light = survived). Annotated calibration statistics: Brier score, calibration slope, and calibration-in-the-large (CITL). ABE alactic base excess, CITL calibration-in-the-large, NISS New Injury Severity Score",
  "FigureS2" = "Figure S2. CONSORT-style participant flow diagram showing step-by-step admission and patient counts, from the total adult MIMIC-IV v3.1 admission pool through to the final study cohort (n=134).",
  "FigureS3" = sprintf("Figure S3. Comparison of ABE discriminatory performance: primary cohort (ABG within 120 min, n=134) versus pre-resuscitation sensitivity cohort (ABG within 60 min, n=%d). ABE alactic base excess, ABG arterial blood gas, AUC area under the ROC curve", n_valid_abe_60m),
  "FigureS4" = "Figure S4. Decision curve analysis showing net benefit across a range of threshold probabilities for (a) the base clinical model with and without ABE, and (b) TRISS with and without ABE, for in-hospital mortality."
)

# Each figure's true saved aspect ratio (width, height in inches), so embedding
# in this Word summary scales correctly instead of distorting wider figures.
fig_dims <- list(
  "Fig1"     = c(w = FW_SINGLE,        h = FH_STD),
  "FigureS1" = c(w = FW_SINGLE,        h = FH_STD),
  "FigureS2" = c(w = FW_SINGLE * 1.55, h = FH_STD * 1.6),
  "FigureS3" = c(w = FW_SINGLE,        h = FH_STD),
  "FigureS4" = c(w = FW_SINGLE * 1.7,  h = FH_STD)
)
embed_width <- 6  # inches; reasonable width for embedding in this report

for (fig_name in names(fig_captions)) {
  png_file <- file.path(fig_dir_word, paste0(fig_name, ".png"))
  if (file.exists(png_file)) {
    dims <- fig_dims[[fig_name]]
    embed_height <- embed_width * (dims["h"] / dims["w"])
    doc <- doc %>%
      h2(fig_name) %>%
      note(fig_captions[[fig_name]]) %>%
      body_add_img(png_file, width = embed_width, height = as.numeric(embed_height)) %>%
      body_add_par("",style="Normal")
  }
}

out_path <- file.path(out_dir,"ABE_Analysis_Output.docx")
print(doc,target=out_path)

cat("\n==============================================================\n")
cat("✓  Analysis complete.\n")
cat("   Word document    :",out_path,"\n")
cat("   Publication figures (PDF):",fig_dir_pdf,"\n")
cat("   Word figures (PNG):",fig_dir_word,"\n")
cat("==============================================================\n")
```
