# Supplementary material for: Alactic Base Excess as a Predictor of Mortality in Trauma ICU Patients: Retrospective Study and Evaluation of Integration into Trauma Scoring Systems
Source: Life (Basel). 2026 Oct 10;16(10):1701. doi: 10.3390/life16101701 (PMC13706226; doi:10.3390/life16101701)
Supplement: Supplementary file 1 [file life-16-01701-s001.zip › FileS2.pdf]

# BigQuery SQL Extraction Queries — MIMIC-IV v3.1

File S2 · Schöllhorn FJ, Azamfirei L, Branea OE · Google BigQuery Standard SQL

This document contains all BigQuery SQL queries used to extract and process data from MIMIC-IV v3.1 (PhysioNet; project physionet-data). Queries are presented in execution order. The intermediate ICDPIC-R step (injury severity scoring in R) must be completed before running Queries 1–7. All queries reference the table thesis-488517.thesis_data_us.icdpicr_cohort, which is the ICDPIC-R output uploaded back to BigQuery.

## Execution Pipeline

| Step | Query | Output / Purpose |
|---|---|---|
| 1 | Cohort Data (Master extraction) | PATIENTS_FINAL_120M.csv → feeds ICDPIC-R in R |
| ↓ | ICDPIC-R (in R) | Calculates ISS, NISS, RTS, TRISS, mechanism → uploaded back to BigQuery as icdpicr_cohort |
| 2 | Cohort Flowchart | Step-by-step admission AND patient counts, every box including excluded (Figure S2) |
| 3 | Outcomes + Hospital LOS | Joined into cohort_final.csv: died_inhosp, died_30day, hospital_los_days |
| 4 | ICU LOS | Joined into cohort_final.csv: icu_los_days |
| 5 | Admission Vitals | Joined into cohort_final.csv: HR, SBP, MAP, $SpO_2$, temperature |
| 6 | GCS | Joined into cohort_final.csv: gcs_verbal, gcs_motor, gcs_eye, gcs_total |
| 7 | Admission Labs | Joined into cohort_final.csv: Hb, Hct, platelets, INR, creatinine, BUN |
| 8 | BE + Lactate check (verification) | Quality-control check only (not used in analysis) |
| 9 | 60-min sensitivity | Patients_ICDPICR_60Minutes.xlsx (pre-resuscitation sensitivity cohort) |
| 10 | Excluded-population characteristics | Age, sex, mortality of admissions excluded for missing an early ABG (Table S2) |

## Query 1 — Cohort Data — Master Extraction Query

*Output: Export result as: PATIENTS_FINAL_120M.csv | Requires: Run before any other query*

Extracts the base cohort: all adult ICU patients with trauma ICD-10 codes (S00–T88), valid base excess and lactate within ±60–120 min of admission, and no exclusion diagnoses. Calculates ABE = base_excess + lactate. Aggregates ICD codes for ICDPIC-R processing.

```
WITH base_population AS (
  SELECT
    adm.subject_id,
    adm.hadm_id,
    adm.admittime,
    pat.anchor_age AS age,
    pat.gender
  FROM `physionet-data.mimiciv_3_1_hosp.admissions` adm
  JOIN `physionet-data.mimiciv_3_1_hosp.patients` pat ON adm.subject_id = pat.subject_id
  WHERE pat.anchor_age >= 18
),
icu_filter AS (
  SELECT DISTINCT hadm_id FROM `physionet-data.mimiciv_3_1_icu.icustays`
),
trauma_filter AS (
  SELECT DISTINCT hadm_id
```

```
  FROM `physionet-data.mimiciv_3_1_hosp.diagnoses_icd`
  WHERE icd_code LIKE 'S%' OR icd_code LIKE 'T%'
),
exclusion_ids AS (
  SELECT DISTINCT hadm_id
  FROM `physionet-data.mimiciv_3_1_hosp.diagnoses_icd`
  WHERE
    REGEXP_CONTAINS(icd_code, '^N18[3456]')
    OR icd_code = 'Z992'
    OR REGEXP_CONTAINS(icd_code, '^E101|^E111|^E121|^E131|^E141|^E1065|^E110|^E130')
    OR REGEXP_CONTAINS(icd_code, '^E222|^E870|^E835|^E20|^E21|^E24|^E26|^E27')
    OR REGEXP_CONTAINS(icd_code, '^K720|^K729|^K73|^K746|^K7689')
    OR icd_code = 'E244'
    OR REGEXP_CONTAINS(icd_code, '^T383|^T39011|^T511|^T528|^T573|^T5801')
    OR icd_code LIKE 'J44%'
    OR icd_code = 'J9611'
    OR icd_code = 'R572'
    OR icd_code = 'Z9911'
    OR REGEXP_CONTAINS(icd_code, '^E71[123]|^G713')
    OR icd_code = 'Z331'
    OR icd_code LIKE 'O%'
),
first_labs AS (
  SELECT
    le.hadm_id,
    MAX(CASE WHEN itemid = 50802 THEN valuenum END) AS base_excess,
    MAX(CASE WHEN itemid = 50813 THEN valuenum END) AS lactate,
    MIN(charttime) AS lab_time
  FROM `physionet-data.mimiciv_3_1_hosp.labevents` le
  JOIN base_population bp ON le.hadm_id = bp.hadm_id
  WHERE itemid IN (50802, 50813)
    AND DATETIME_DIFF(le.charttime, bp.admittime, MINUTE) BETWEEN -60 AND 120
  GROUP BY le.hadm_id
  HAVING base_excess IS NOT NULL AND lactate IS NOT NULL
),
dx_agg AS (
  -- Aggregate all ICD codes into a comma-separated string for ICDPIC-R
  SELECT
    hadm_id,
    STRING_AGG(icd_code, ',' ORDER BY seq_num) AS icd_codes
  FROM `physionet-data.mimiciv_3_1_hosp.diagnoses_icd`
  GROUP BY hadm_id
)
SELECT
  p.subject_id,
  p.hadm_id,
  p.age,
  p.gender,
  l.base_excess,
  l.lactate,
  (l.base_excess + l.lactate) AS alactic_base_excess,
  l.lab_time,
  p.admittime,
  d.icd_codes
FROM base_population p
INNER JOIN icu_filter i ON p.hadm_id = i.hadm_id
INNER JOIN trauma_filter t ON p.hadm_id = t.hadm_id
INNER JOIN first_labs l ON p.hadm_id = l.hadm_id
INNER JOIN dx_agg d ON p.hadm_id = d.hadm_id
WHERE p.hadm_id NOT IN (SELECT hadm_id FROM exclusion_ids);
```

## Query 2 — Cohort Flowchart — Full Box-Level Counts

*Output: Export result as FlowchartCounts.csv*

Produces admission AND patient counts for every box in the participant flow diagram (Figure S2), including the excluded side-boxes — computed via direct anti-joins rather than subtraction, since patient-level subtraction is not always valid when a patient has multiple admissions spanning both sides of a step (confirmed necessary at the missing-ABG step).

```
-- Query 2: Cohort Flowchart — full box-level counts, admission AND
-- patient level, for both "kept" and "excluded" boxes at every step.
-- Patient-level counts use direct anti-join queries rather than subtraction,
-- since a small number of patients have multiple admissions that can span
-- both sides of a given step (confirmed necessary at the "missing ABG" step).
-- Output: Export result as FlowchartCounts.csv
```

```
WITH base_admissions AS (
  SELECT adm.hadm_id, adm.subject_id
  FROM `physionet-data.mimiciv_3_1_hosp.admissions` adm
  JOIN `physionet-data.mimiciv_3_1_hosp.patients` pat ON adm.subject_id = pat.subject_id
  WHERE pat.anchor_age >= 18
),
icu_patients AS (
  SELECT DISTINCT hadm_id FROM `physionet-data.mimiciv_3_1_icu.icustays`
),
trauma_patients AS (
  SELECT DISTINCT hadm_id
  FROM `physionet-data.mimiciv_3_1_hosp.diagnoses_icd`
  WHERE icd_code LIKE 'S%' OR icd_code LIKE 'T%'
),
valid_labs AS (
  SELECT le.hadm_id,
         MAX(CASE WHEN itemid = 50802 THEN valuenum END) AS base_excess,
         MAX(CASE WHEN itemid = 50813 THEN valuenum END) AS lactate
  FROM `physionet-data.mimiciv_3_1_hosp.labevents` le
  JOIN `physionet-data.mimiciv_3_1_hosp.admissions` adm ON le.hadm_id = adm.hadm_id
  WHERE itemid IN (50802, 50813)
    AND DATETIME_DIFF(le.charttime, adm.admittime, MINUTE) BETWEEN -60 AND 120
  GROUP BY le.hadm_id
  HAVING base_excess IS NOT NULL AND lactate IS NOT NULL
),
exclusions AS (
  SELECT DISTINCT hadm_id
  FROM `physionet-data.mimiciv_3_1_hosp.diagnoses_icd`
  WHERE REGEXP_CONTAINS(icd_code, '^N18[3456]')
     OR icd_code = 'Z992'
     OR REGEXP_CONTAINS(icd_code, '^E101|^E111|^E121|^E131|^E141|^E1065|^E110|^E130')
     OR REGEXP_CONTAINS(icd_code, '^E222|^E870|^E835|^E20|^E21|^E24|^E26|^E27')
     OR REGEXP_CONTAINS(icd_code, '^K720|^K729|^K73|^K746|^K7689')
     OR icd_code = 'E244'
     OR REGEXP_CONTAINS(icd_code, '^T383|^T39011|^T511|^T528|^T573|^T5801')
     OR icd_code LIKE 'J44%'
     OR icd_code = 'J9611'
     OR icd_code = 'R572'
     OR icd_code = 'Z9911'
     OR REGEXP_CONTAINS(icd_code, '^E71[123]|^G713')
     OR icd_code = 'Z331'
     OR icd_code LIKE 'O%'
),
-- Step-by-step admission sets (each a strict subset of the previous)
s1 AS (SELECT * FROM base_admissions),
s2 AS (SELECT * FROM s1 WHERE hadm_id IN (SELECT hadm_id FROM icu_patients)),
s3 AS (SELECT * FROM s2 WHERE hadm_id IN (SELECT hadm_id FROM trauma_patients)),
s4 AS (SELECT * FROM s3 WHERE hadm_id IN (SELECT hadm_id FROM valid_labs)),
s5 AS (SELECT * FROM s4 WHERE hadm_id NOT IN (SELECT hadm_id FROM exclusions))

SELECT '1. Total Adult Admissions' AS step,
       COUNT(DISTINCT hadm_id) AS admission_count, COUNT(DISTINCT subject_id) AS patient_count
FROM s1
UNION ALL
SELECT '1b. Non-ICU admissions excluded',
       (SELECT COUNT(DISTINCT hadm_id) FROM s1) - (SELECT COUNT(DISTINCT hadm_id) FROM s2),
       (SELECT COUNT(DISTINCT subject_id) FROM s1 WHERE subject_id NOT IN (SELECT subject_id FROM
s2))
UNION ALL
SELECT '2. ICU Admissions', COUNT(DISTINCT hadm_id), COUNT(DISTINCT subject_id) FROM s2
UNION ALL
SELECT '2b. No trauma ICD-10 code',
       (SELECT COUNT(DISTINCT hadm_id) FROM s2) - (SELECT COUNT(DISTINCT hadm_id) FROM s3),
       (SELECT COUNT(DISTINCT subject_id) FROM s2 WHERE subject_id NOT IN (SELECT subject_id FROM
s3))
UNION ALL
SELECT '3. Trauma ICD-10 Code Present (S00-T88)', COUNT(DISTINCT hadm_id), COUNT(DISTINCT
subject_id) FROM s3
UNION ALL
SELECT '3b. Missing ABG <=2h or incomplete data',
       (SELECT COUNT(DISTINCT hadm_id) FROM s3) - (SELECT COUNT(DISTINCT hadm_id) FROM s4),
       (SELECT COUNT(DISTINCT subject_id) FROM s3 WHERE subject_id NOT IN (SELECT subject_id FROM
s4))
UNION ALL
SELECT '4. ABG <=2h + Complete Data Available', COUNT(DISTINCT hadm_id), COUNT(DISTINCT subject_id)
FROM s4
```

```
UNION ALL
SELECT '4b. Exclusion diagnoses present',
       (SELECT COUNT(DISTINCT hadm_id) FROM s4) - (SELECT COUNT(DISTINCT hadm_id) FROM s5),
       (SELECT COUNT(DISTINCT subject_id) FROM s4 WHERE subject_id NOT IN (SELECT subject_id FROM
s5))
UNION ALL
SELECT '5. Final Cohort (Exclusions Removed)', COUNT(DISTINCT hadm_id), COUNT(DISTINCT subject_id)
FROM s5;
```

## Query 3 — Outcomes and Hospital Length of Stay

*Output: Join into cohort_final.csv | Requires: icdpicr_cohort in BigQuery*

Extracts in-hospital mortality (hospital_expire_flag), 30-day mortality (derived from date of death), and hospital LOS in days. Filters to the ICDPIC-R–processed cohort.

```
-- Query 3: Outcomes and Hospital Length of Stay
SELECT
  a.subject_id,
  a.hadm_id,
  a.admittime,
  a.dischtime,
  a.deathtime,
  a.hospital_expire_flag,
  p.dod,
  DATETIME_DIFF(
    CAST(a.dischtime AS DATETIME),
    CAST(a.admittime AS DATETIME),
    HOUR
  ) / 24.0 AS hospital_los_days,
  CASE
    WHEN p.dod IS NOT NULL
      AND DATE_DIFF(p.dod, DATE(CAST(a.admittime AS DATETIME)), DAY) <= 30
    THEN 1 ELSE 0
  END AS mortality_30day
FROM `physionet-data.mimiciv_3_1_hosp.admissions` a
INNER JOIN `thesis-488517.thesis_data_us.icdpicr_cohort` c
  ON a.hadm_id = CAST(c.hadm_id AS INT64)
LEFT JOIN `physionet-data.mimiciv_3_1_hosp.patients` p
  ON a.subject_id = p.subject_id;
```

## Query 4 — ICU Length of Stay

*Output: Join into cohort_final.csv | Requires: icdpicr_cohort in BigQuery*

Sums ICU stay duration (in days) across all ICU admissions within a hospitalisation for each patient in the processed cohort.

```
-- Query 4: ICU Length of Stay
SELECT
  i.subject_id,
  i.hadm_id,
  SUM(i.los) AS icu_los_days
FROM `physionet-data.mimiciv_3_1_icu.icustays` i
INNER JOIN `thesis-488517.thesis_data_us.icdpicr_cohort` c
  ON i.hadm_id = CAST(c.hadm_id AS INT64)
GROUP BY i.subject_id, i.hadm_id;
```

## Query 5 — Admission Vital Signs

*Output: Join into cohort_final.csv | Requires: icdpicr_cohort in BigQuery*

Retrieves the first recorded value within 6 hours of admission for: heart rate, systolic/diastolic/mean arterial blood pressure, respiratory rate, $SpO_2$, and temperature. Uses chartevents (ICU charting system).

```
-- Query 5: Admission Vital Signs (first value within 6 hours of admission)
WITH vitals_raw AS (
  SELECT
    ce.subject_id,
    ce.hadm_id,
    ce.itemid,
    ce.valuenum,
    ce.charttime,
    a.admittime,
    ROW_NUMBER() OVER (
      PARTITION BY ce.hadm_id, ce.itemid
```

```
      ORDER BY ce.charttime ASC
    ) AS rn
  FROM `physionet-data.mimiciv_3_1_icu.chartevents` ce
  INNER JOIN `thesis-488517.thesis_data_us.icdpicr_cohort` c
    ON ce.hadm_id = CAST(c.hadm_id AS INT64)
  JOIN `physionet-data.mimiciv_3_1_hosp.admissions` a
    ON ce.hadm_id = a.hadm_id
  WHERE ce.valuenum IS NOT NULL
    AND DATETIME_DIFF(ce.charttime, CAST(a.admittime AS DATETIME), MINUTE) BETWEEN 0 AND 360
    AND ce.itemid IN (
      220045,          -- Heart rate
      220179, 220050,  -- Systolic BP (non-invasive, invasive)
      220180, 220051,  -- Diastolic BP
      220052, 220181,  -- Mean arterial pressure
      220210,          -- Respiratory rate
      220277,          -- SpO2
      223762, 223761   -- Temperature (Celsius, Fahrenheit)
    )
)
SELECT
  subject_id,
  hadm_id,
  MAX(CASE WHEN itemid = 220045 THEN valuenum END) AS heart_rate,
  MAX(CASE WHEN itemid IN (220179, 220050) THEN valuenum END) AS sbp,
  MAX(CASE WHEN itemid IN (220180, 220051) THEN valuenum END) AS dbp,
  MAX(CASE WHEN itemid IN (220052, 220181) THEN valuenum END) AS map,
  MAX(CASE WHEN itemid = 220210 THEN valuenum END) AS resp_rate,
  MAX(CASE WHEN itemid = 220277 THEN valuenum END) AS spo2,
  MAX(CASE WHEN itemid IN (223762, 223761) THEN valuenum END) AS temperature
FROM vitals_raw
WHERE rn = 1
GROUP BY subject_id, hadm_id;
```

## Query 6 — Glasgow Coma Scale

*Output: Join into cohort_final.csv | Requires: icdpicr_cohort in BigQuery*

Retrieves verbal, motor, and eye components of GCS (itemIDs 223900, 223901, 220739) within 2 hours of admission. Calculates gcs_total as the sum. Missing where patients were intubated at admission (47% of cohort).

```
-- Query 6: Glasgow Coma Scale (first value within 2 hours of admission)
WITH gcs_raw AS (
  SELECT
    ce.subject_id,
    ce.hadm_id,
    ce.itemid,
    ce.valuenum,
    ce.charttime,
    a.admittime,
    ROW_NUMBER() OVER (
      PARTITION BY ce.hadm_id, ce.itemid
      ORDER BY ce.charttime ASC
    ) AS rn
  FROM `physionet-data.mimiciv_3_1_icu.chartevents` ce
  INNER JOIN `thesis-488517.thesis_data_us.icdpicr_cohort` c
    ON ce.hadm_id = CAST(c.hadm_id AS INT64)
  JOIN `physionet-data.mimiciv_3_1_hosp.admissions` a
    ON ce.hadm_id = a.hadm_id
  WHERE ce.valuenum IS NOT NULL
    AND DATETIME_DIFF(ce.charttime, CAST(a.admittime AS DATETIME), MINUTE) BETWEEN 0 AND 120
    AND ce.itemid IN (
      223900,  -- GCS verbal
      223901,  -- GCS motor
      220739   -- GCS eye opening
    )
)
SELECT
  subject_id,
  hadm_id,
  MAX(CASE WHEN itemid = 223900 THEN valuenum END) AS gcs_verbal,
  MAX(CASE WHEN itemid = 223901 THEN valuenum END) AS gcs_motor,
  MAX(CASE WHEN itemid = 220739 THEN valuenum END) AS gcs_eye,
  MAX(CASE WHEN itemid = 223900 THEN valuenum END) +
  MAX(CASE WHEN itemid = 223901 THEN valuenum END) +
  MAX(CASE WHEN itemid = 220739 THEN valuenum END) AS gcs_total
FROM gcs_raw
WHERE rn = 1
```

```
GROUP BY subject_id, hadm_id;
```

## Query 7 — Admission Laboratory Values

*Output: Join into cohort_final.csv | Requires: icdpicr_cohort in BigQuery*

Retrieves first values within 6 hours of admission for: haemoglobin, haematocrit, platelets, INR, creatinine, and BUN. Used for descriptive characterisation; not included as model predictors.

```
-- Query 7: Admission Laboratory Values (first value within 6 hours of admission)
WITH labs_raw AS (
  SELECT
    le.subject_id,
    le.hadm_id,
    le.itemid,
    le.valuenum,
    le.charttime,
    a.admittime,
    ROW_NUMBER() OVER (
      PARTITION BY le.hadm_id, le.itemid
      ORDER BY le.charttime ASC
    ) AS rn
  FROM `physionet-data.mimiciv_3_1_hosp.labevents` le
  INNER JOIN `thesis-488517.thesis_data_us.icdpicr_cohort` c
    ON le.hadm_id = CAST(c.hadm_id AS INT64)
  JOIN `physionet-data.mimiciv_3_1_hosp.admissions` a
    ON le.hadm_id = a.hadm_id
  WHERE le.valuenum IS NOT NULL
    AND DATETIME_DIFF(
      CAST(le.charttime AS DATETIME),
      CAST(a.admittime AS DATETIME),
      MINUTE) BETWEEN 0 AND 360
    AND le.itemid IN (
      51222,  -- Haemoglobin
      51221,  -- Haematocrit
      51265,  -- Platelets
      51237,  -- INR
      50912,  -- Creatinine
      51006   -- BUN
    )
)
SELECT
  subject_id,
  hadm_id,
  MAX(CASE WHEN itemid = 51222 THEN valuenum END) AS haemoglobin,
  MAX(CASE WHEN itemid = 51221 THEN valuenum END) AS haematocrit,
  MAX(CASE WHEN itemid = 51265 THEN valuenum END) AS platelets,
  MAX(CASE WHEN itemid = 51237 THEN valuenum END) AS inr,
  MAX(CASE WHEN itemid = 50912 THEN valuenum END) AS creatinine,
  MAX(CASE WHEN itemid = 51006 THEN valuenum END) AS bun
FROM labs_raw
WHERE rn = 1
GROUP BY subject_id, hadm_id;
```

## Query 8 — Base Excess and Lactate Verification Check

*Output: Quality-control verification only — not used in analysis*

Checks raw base excess and lactate values returned within the 120-minute window, ordered by highest lactate. Used to verify extraction logic and detect date-format artefacts before finalising cohort_final.csv.

```
-- Query 8 (Verification): Base Excess and Lactate -- Quality Control Check
-- Used to verify extraction and detect date-format artefacts. Not used in analysis.
WITH abg_raw AS (
  SELECT
    le.subject_id,
    le.hadm_id,
    le.itemid,
    le.valuenum,
    le.charttime,
    a.admittime,
    ROW_NUMBER() OVER (
      PARTITION BY le.hadm_id, le.itemid
      ORDER BY le.charttime ASC
    ) AS rn
  FROM `physionet-data.mimiciv_3_1_hosp.labevents` le
  INNER JOIN `thesis-488517.thesis_data_us.icdpicr_cohort` c
```

```
    ON le.hadm_id = CAST(c.hadm_id AS INT64)
  JOIN `physionet-data.mimiciv_3_1_hosp.admissions` a
    ON le.hadm_id = a.hadm_id
  WHERE le.valuenum IS NOT NULL
    AND DATETIME_DIFF(
      CAST(le.charttime AS DATETIME),
      CAST(a.admittime AS DATETIME),
      MINUTE) BETWEEN 0 AND 120
    AND le.itemid IN (50802, 50813, 52442)
)
SELECT
  subject_id,
  hadm_id,
  MAX(CASE WHEN itemid = 50802 THEN valuenum END) AS base_excess_bg,
  MAX(CASE WHEN itemid IN (50813, 52442) THEN valuenum END) AS lactate_bg,
  COUNT(DISTINCT itemid) AS n_items_found
FROM abg_raw
WHERE rn = 1
GROUP BY subject_id, hadm_id
ORDER BY lactate_bg DESC;
```

## Query 9 — 60-Minute Sensitivity Cohort

*Output: Export result as: Patients_ICDPICR_60Minutes.xlsx | Requires: icdpicr_cohort in BigQuery*

Re-extracts base excess and lactate restricting the window to ≤60 minutes from admission (pre-resuscitation sensitivity analysis). 7 ABE values excluded post-export as date-format artefacts; effective analytic n = 54.

```
-- Query 9: Base Excess and Lactate -- 60-minute admission window
-- Pre-resuscitation sensitivity analysis cohort
WITH abg_raw AS (
  SELECT
    le.subject_id,
    le.hadm_id,
    le.itemid,
    le.valuenum,
    le.charttime,
    a.admittime,
    ROW_NUMBER() OVER (
      PARTITION BY le.hadm_id, le.itemid
      ORDER BY le.charttime ASC
    ) AS rn
  FROM `physionet-data.mimiciv_3_1_hosp.labevents` le
  INNER JOIN `thesis-488517.thesis_data_us.icdpicr_cohort` c
    ON le.hadm_id = CAST(c.hadm_id AS INT64)
  JOIN `physionet-data.mimiciv_3_1_hosp.admissions` a
    ON le.hadm_id = a.hadm_id
  WHERE le.valuenum IS NOT NULL
    AND DATETIME_DIFF(
      CAST(le.charttime AS DATETIME),
      CAST(a.admittime AS DATETIME),
      MINUTE) BETWEEN 0 AND 60
    AND le.itemid IN (
      50802,  -- Base Excess
      50813,  -- Lactate
      52442   -- Lactate (alternate itemID)
    )
)
SELECT
  subject_id,
  hadm_id,
  MAX(CASE WHEN itemid = 50802 THEN valuenum END) AS base_excess_1h,
  MAX(CASE WHEN itemid IN (50813, 52442) THEN valuenum END) AS lactate_1h
FROM abg_raw
WHERE rn = 1
GROUP BY subject_id, hadm_id
ORDER BY hadm_id;
```

## Query 10 — Characteristics of Patients Excluded for Missing Early ABG

*Output: Reported directly in manuscript text and Table S2 | Requires: no dependency on icdpicr_cohort — runs against raw MIMIC-IV tables only*

Characterises the 15,987 admissions with a trauma ICD-10 diagnosis and ICU stay that lacked a valid ABE+lactate measurement within −60 to +120 minutes of admission, reporting age, sex, and in-hospital mortality for comparison against

the final analytic cohort (Table 1), and patient count using the same strict definition as Figure S2. Used to assess selection bias introduced by the early-ABG inclusion criterion.

```
-- Query 10: Characteristics of Patients Excluded for Missing Early ABG
-- Characterises the 15,987 admissions with a trauma
-- ICD-10 diagnosis and ICU stay that lacked a valid ABE+lactate measurement within
-- -60 to +120 minutes of admission. Patient-level count uses the same strict
-- definition as Query 2 (patients with ZERO qualifying admissions, not just at
-- least one non-qualifying admission), so this stays consistent with Figure S2
-- (13,788 patients).

WITH base_population AS (
  SELECT
    adm.subject_id,
    adm.hadm_id,
    adm.admittime,
    adm.hospital_expire_flag,
    pat.anchor_age AS age,
    pat.gender
  FROM `physionet-data.mimiciv_3_1_hosp.admissions` adm
  JOIN `physionet-data.mimiciv_3_1_hosp.patients` pat ON adm.subject_id = pat.subject_id
  WHERE pat.anchor_age >= 18
),
icu_filter AS (
  SELECT DISTINCT hadm_id FROM `physionet-data.mimiciv_3_1_icu.icustays`
),
trauma_filter AS (
  SELECT DISTINCT hadm_id
  FROM `physionet-data.mimiciv_3_1_hosp.diagnoses_icd`
  WHERE icd_code LIKE 'S%' OR icd_code LIKE 'T%'
),
valid_labs AS (
  SELECT le.hadm_id,
         MAX(CASE WHEN itemid = 50802 THEN valuenum END) AS base_excess,
         MAX(CASE WHEN itemid = 50813 THEN valuenum END) AS lactate
  FROM `physionet-data.mimiciv_3_1_hosp.labevents` le
  JOIN `physionet-data.mimiciv_3_1_hosp.admissions` adm ON le.hadm_id = adm.hadm_id
  WHERE itemid IN (50802, 50813)
    AND DATETIME_DIFF(le.charttime, adm.admittime, MINUTE) BETWEEN -60 AND 120
  GROUP BY le.hadm_id
  HAVING base_excess IS NOT NULL AND lactate IS NOT NULL
),
trauma_icu_admissions AS (
  SELECT * FROM base_population
  WHERE hadm_id IN (SELECT hadm_id FROM icu_filter)
    AND hadm_id IN (SELECT hadm_id FROM trauma_filter)
),
eligible_no_abg_admissions AS (
  -- Admissions excluded for missing early ABG (15,987 admissions)
  SELECT * FROM trauma_icu_admissions
  WHERE hadm_id NOT IN (SELECT hadm_id FROM valid_labs)
),
patients_with_any_qualifying_admission AS (
  SELECT DISTINCT subject_id FROM trauma_icu_admissions
  WHERE hadm_id IN (SELECT hadm_id FROM valid_labs)
),
excluded_patients_strict AS (
  -- Patients with ZERO qualifying admissions (matches Figure S2's 13,788)
  SELECT DISTINCT subject_id
  FROM eligible_no_abg_admissions
  WHERE subject_id NOT IN (SELECT subject_id FROM patients_with_any_qualifying_admission)
)
SELECT
  (SELECT COUNT(DISTINCT hadm_id) FROM eligible_no_abg_admissions) AS n_admissions,
  (SELECT COUNT(*) FROM excluded_patients_strict) AS n_patients,
  ROUND(AVG(age), 1) AS mean_age,
  APPROX_QUANTILES(age, 2)[OFFSET(1)] AS median_age,
  ROUND(100 * SUM(CASE WHEN gender = 'M' THEN 1 ELSE 0 END) / COUNT(*), 1) AS pct_male,
  SUM(hospital_expire_flag) AS n_deaths,
  ROUND(100 * SUM(hospital_expire_flag) / COUNT(*), 1) AS pct_mortality
FROM eligible_no_abg_admissions;
```
