# Supplementary material for: Alactic Base Excess as a Predictor of Mortality in Trauma ICU Patients: Retrospective Study and Evaluation of Integration into Trauma Scoring Systems
Source: Life (Basel). 2026 Oct 10;16(10):1701. doi: 10.3390/life16101701 (PMC13706226; doi:10.3390/life16101701)
Supplement: Supplementary file 1 [file life-16-01701-s001.zip › FileS3.pdf]

STROBE Statement — Cohort Study Checklist

File S3 · Schöllhorn FJ, Azamfirei L, Branea OE · Alactic Base Excess as a Predictor of Mortality in Trauma ICU Patients

Item No.	Recommendation	Page No.	Reported as / relevant text
**TITLE & ABSTRACT**			
**1(a)**	Indicate the study's design with a commonly used term in the title or the abstract	**1**	“Retrospective Study” stated explicitly in the title: “Alactic Base Excess as a Predictor of Mortality in Trauma ICU Patients: Retrospective Study and Evaluation of Integration into Trauma Scoring Systems”
**1(b)**	Provide an informative and balanced summary of what was done and what was found	**1**	Single unstructured abstract paragraph summarising design, cohort, key findings (including the lactate-overlap and decision curve analysis results), and the hypothesis-generating framing.
**INTRODUCTION**			
**2**	Explain the scientific background and rationale for the investigation being reported	**2**	Introduction outlines global trauma mortality burden, the limitations of lactate and BE individually, and the evidence gap for ABE specifically in severe trauma.
**3**	State specific objectives, including any prespecified hypotheses	**2**	“The goals of this study were to: (1) determine whether ABE at admission is an independent predictor… and (2) assess whether ABE provides incremental prognostic value…”
**METHODS**			
**4**	Present key elements of study design early in the paper	**2**	“This retrospective, observational cohort study used… MIMIC-IV v3.1…” (Section 2.1).
**5**	Describe the setting, locations, and relevant dates, including periods of recruitment, exposure, follow-up, and data collection	**2**	BIDMC, Boston, USA; 2008–2022 (Section 2.1).
**6(a)**	Give the eligibility criteria, and the sources and methods of selection of participants	**3**	Inclusion/exclusion criteria (Section 2.3). Exclusions use a three-tier hierarchy (Table S1): Tier 1 direct/established effect, Tier 2 plausible/indirect, Tier 3 no established effect.
**6(b)**	Cohort study — for matched studies, give matching criteria and number of exposed/unexposed	**N/A**	N/A — not a matched design.
**7**	Clearly define all outcomes, exposures, predictors, potential confounders, and effect modifiers. Give diagnostic criteria, if applicable	**3–4**	Predictor (ABE) and co-predictors age/NISS/mechanism defined (Section 2.5); outcomes defined (Section 2.6); eligibility-related confounders addressed via exclusion criteria (Section 2.3).
**8***	For each variable of interest, give sources of data and details of methods of assessment (measurement). Describe comparability of assessment methods if more than one group	**3–4**	MIMIC-IV v3.1 extracted via BigQuery SQL (File S2). ABE from labevents; ISS/NISS via ICDPIC-R; TRISS via Boyd et al. coefficients (Section 2.4–2.5).
**9**	Describe any efforts to address potential sources of bias	**3**	Date-format artefact screening (confirmed applied to both cohorts, zero records affected in the primary cohort); multivariable adjustment; MICE for missing covariates (Section 2.4).
**10**	Explain how the study size was arrived at	**4**	No formal sample size calculation; all eligible patients included (n=134, 39 events); events-per-variable 7.8, acknowledged as at the lower boundary of convention (Section 2.7).
**11**	Explain how quantitative variables were handled in the analyses. If applicable, describe which groupings were chosen and why	**4**	Continuous predictors entered untransformed; Youden-derived threshold reported descriptively, not used in regression models (Section 2.7).
**12(a)**	Describe all statistical methods, including those used to control for confounding	**4–5**	Multivariable logistic regression (3 models); MICE (m=20, Rubin's rules); DeLong's method; Hosmer-Lemeshow, Brier, CITL, calibration slope; LRT/NRI/IDI; decision curve analysis (Section 2.7).
**12(b)**	Describe any methods used to examine subgroups and interactions	**5**	Five prespecified sensitivity analyses (Section 2.7); no formal interaction analyses.
**12(c)**	Explain how missing data were addressed	**4**	MICE, m=20, predictive mean matching, pooled by Rubin's rules. GCS missing 47% (intubation-related), SBP 10.4%; all primary predictors complete.

Item No.	Recommendation	Page No.	Reported as / relevant text
**12(d)**	Cohort study — if applicable, explain how loss to follow-up was addressed	**3, 5**	Retrospective closed-database study; in-hospital outcomes complete for all 134 patients; 30-day mortality via MIMIC-IV dod field. No loss to follow-up.
**12(e)**	Describe any sensitivity analyses	**5**	Five sensitivity analyses: 60-minute ABG window (Firth penalised regression); sepsis-diagnosis exclusion; complete-case TRISS (observed GCS only); alcohol-code exclusion; creatinine-adjusted model.
**RESULTS**			
**13(a)**	Report the numbers of individuals at each stage of the study	**5**	Of 17,599 trauma-coded ICU admissions, 134 met all inclusion criteria; full step-by-step counts in the participant flow diagram (Figure S2).
**13(b)**	Give reasons for non-participation at each stage	**5**	Reasons given at each step of the flow diagram (Figure S2): non-ICU admission, no trauma code, missing early ABG, exclusion diagnoses, ISS <16.
**13(c)**	Consider use of a flow diagram	**5**	Participant flow diagram provided as Figure S2.
**14(a)***	Give characteristics of study participants and information on exposures and potential confounders	**5–6**	Table 1: baseline characteristics stratified by in-hospital mortality outcome (age, sex, mechanism, ISS, NISS, ABE, BE, lactate, GCS, SBP, LOS).
**14(b)***	Indicate the number of participants with missing data for each variable of interest	**4, 6**	GCS missing in 47% (n=63, predominantly intubation), SBP missing in 10.4% (n=14); all other variables complete (Section 2.7; Table 1 footnote).
**14(c)***	Cohort study — summarise follow-up time	**5**	Complete in-hospital follow-up for all 134 patients; hospital LOS median 10.5 days [IQR 3.4–20.8] (Table 1).
**15***	Report numbers of outcome events or summary measures	**5**	In-hospital mortality 29.1% (n=39); 30-day mortality 31.3% (n=42) (Results 3.1, Table 1).
**16(a)**	Give unadjusted estimates and, if applicable, confounder-adjusted estimates and their precision. Clarify confounders adjusted for and why	**6–7**	Univariable OR 0.870 (p=0.007; Results 3.2). Adjusted primary-model OR 0.840 (95% CI 0.745–0.946; p=0.004; Table 2).
**16(b)**	Report category boundaries when continuous variables were categorized	**6**	Youden threshold −1.45 mmol/L (sensitivity 76.9% [95% CI 64.1–89.7%], specificity 52.6% [95% CI 42.1–63.2%], PPV 40.0% [95% CI 33.8–47.1%], NPV 84.7% [95% CI 77.2–92.5%]); not used for model classification (Results 3.2).
**16(c)**	If relevant, consider translating estimates of relative risk into absolute risk for a meaningful time period	**7**	Prediction equation (Equation 2) allows individual probability calculation; explicitly stated as reported for transparency only, not for clinical use without external validation (Results 3.3).
**17**	Report other analyses done — e.g., analyses of subgroups and interactions, and sensitivity analyses	**5–12**	TRISS integration incl. complete-case sensitivity analysis (Results 3.5, Table 4); decision curve analysis (Results 3.4); bootstrap optimism correction (Results 3.4); extended calibration metrics (Results 3.3); alcohol-exclusion and creatinine-adjustment sensitivity analyses (Results 3.6).
**DISCUSSION**			
**18**	Summarise key results with reference to study objectives	**10**	Opening Discussion paragraph: ABE independently associated with in-hospital mortality (OR 0.840); first ABE evaluation in a severe-trauma-defined cohort.
**19**	Discuss limitations of the study, taking into account sources of potential bias or imprecision. Discuss both direction and magnitude of any potential bias	**11–12**	Single-centre cohort; no external validation; GCS imputation; TRISS coefficient vintage; selection into early sampling; alcohol/AKI addressed via sensitivity analyses; no correction for multiple comparisons; no fairness analysis across subgroups (Section 4.1).
**20**	Give a cautious overall interpretation of results considering objectives, limitations, multiplicity of analyses, results from similar studies, and other relevant evidence	**10–12**	ABE's prognostic content overlaps substantially with lactate; incremental value is modest and statistically fragile; findings are hypothesis-generating and should not be applied clinically before prospective, multicentre validation (Discussion and Conclusion).
**21**	Discuss the generalisability (external validity) of the study results	**11–12**	Findings apply only to patients undergoing similarly early metabolic assessment, not all severe trauma ICU admissions;

Item No.	Recommendation	Page No.	Reported as / relevant text
			BIDMC-specific protocols may not generalise; prospective multicentre validation identified as the essential next step.
**OTHER INFORMATION**			
**22**	Give the source of funding and the role of the funders for the present study and, if applicable, for the original study on which the present article is based	**12**	“This research received no external funding. The authors intend to apply for open-access publishing fee support from George Emil Palade University…” No conflicts of interest declared.

** In a single-cohort study with no separate exposure groups, items marked * are reported for the total cohort; outcome stratification (survived vs. died) is presented in Table 1. Note: this checklist covers the cohort-study component of the manuscript; prediction-model reporting follows the TRIPOD+AI statement, provided separately as File S4.*
