# Supplementary material for: Alactic Base Excess as a Predictor of Mortality in Trauma ICU Patients: Retrospective Study and Evaluation of Integration into Trauma Scoring Systems
Source: Life (Basel). 2026 Oct 10;16(10):1701. doi: 10.3390/life16101701 (PMC13706226; doi:10.3390/life16101701)
Supplement: Supplementary file 1 [file life-16-01701-s001.zip › FileS4.pdf]

TRIPOD+AI Checklist

File S4 · Schöllhorn FJ, Azamfirei L, Branea OE · Alactic Base Excess as a Predictor of Mortality in Trauma ICU Patients · Version: 11-January-2024

D = items relevant only to development; E = items relating solely to evaluation; D;E = items applicable to both. This is a development-only study (no external evaluation cohort); items marked E alone are N/A throughout.

Section/Topic	Item	D/E	Checklist Item	Reported on page
**TITLE**				
Title	**1**	D;E	Identify the study as developing or evaluating the performance of a multivariable prediction model, the target population, and the outcome to be predicted	**1**
**ABSTRACT**				
Abstract	**2**	D;E	See TRIPOD+AI for Abstracts checklist	**1**
**INTRODUCTION**				
Background	**3a**	D;E	Explain the healthcare context and rationale for developing or evaluating the prediction model, including references to existing models	**2**
	**3b**	D;E	Describe the target population and the intended purpose of the prediction model in the context of the care pathway, including its intended users	**2**
	**3c**	D;E	Describe any known health inequalities between sociodemographic groups	**N/A**
Objectives	**4**	D;E	Specify the study objectives, including whether the study describes development or validation of a prediction model (or both)	**2**
**METHODS**				
Data	**5a**	D;E	Describe the sources of data separately for development and evaluation datasets, the rationale for using these data, and representativeness	**2**
	**5b**	D;E	Specify the dates of the collected participant data, including start and end of participant accrual, and end of follow-up if applicable	**2**
Participants	**6a**	D;E	Specify key elements of the study setting, including the number and location of centres	**2**
	**6b**	D;E	Describe the eligibility criteria for study participants	**3**
	**6c**	D;E	Give details of any treatments received, and how they were handled, if relevant	**N/A**
Data preparation	**7**	D;E	Describe any data pre-processing and quality checking, including whether this was similar across sociodemographic groups	**3**
Outcome	**8a**	D;E	Clearly define the outcome predicted and the time horizon, including how/when assessed and the rationale for choosing it	**4**
	**8b**	D;E	If outcome assessment requires subjective interpretation, describe assessor qualifications	**N/A**
	**8c**	D;E	Report any actions to blind assessment of the outcome	**N/A**
Predictors	**9a**	D	Describe the choice of initial predictors and any pre-selection before model building	**4**
	**9b**	D;E	Clearly define all predictors, including how and when they were measured	**3–4**
	**9c**	D;E	If predictor measurement requires subjective interpretation, describe assessor qualifications	**N/A**
Sample size	**10**	D;E	Explain how the study size was arrived at and justify sufficiency; include sample size calculation details	**4**
Missing data	**11**	D;E	Describe how missing data were handled; provide reasons for omitting any data	**4**
Analytical methods	**12a**	D	Describe how the data were used (development/evaluation) in the analysis, including any partitioning	**4**
	**12b**	D	Describe how predictors were handled in the analyses (functional form, rescaling, transformation, standardisation)	**4**
	**12c**	D	Specify the type of model, rationale, all model-building steps including hyperparameter tuning, and internal validation method	**4**
	**12d**	D;E	Describe if/how heterogeneity in parameter values and performance was handled across clusters	**N/A**
	**12e**	D;E	Specify all measures/plots used to evaluate model performance and, if relevant, to compare multiple models	**4–5**
	**12f**	E	Describe any model updating arising from model evaluation	**N/A**
	**12g**	E	For model evaluation, describe how model predictions were calculated (formula, code, object, API)	**7**
Class imbalance	**13**	D;E	If class imbalance methods were used, state why and how, and any recalibration methods	**N/A**
Fairness	**14**	D;E	Describe any approaches used to address model fairness and their rationale	**12**

Section/Topic	Item	D/E	Checklist Item	Reported on page
Model output	**15**	D	Specify the output of the prediction model and provide rationale for any classification thresholds	**6–7**
Training vs evaluation	**16**	D;E	Identify any differences between development and evaluation data in setting, eligibility, outcome, predictors	**2**
Ethical approval	**17**	D;E	Name the institutional review board/ethics committee and describe consent or waiver	**2–3, 12–13**
**OPEN SCIENCE**				
Funding	**18a**	D;E	Give the source of funding and the role of the funders	**12**
Conflicts of interest	**18b**	D;E	Declare any conflicts of interest and financial disclosures for all authors	**13**
Protocol	**18c**	D;E	Indicate where the study protocol can be accessed, or state that none was prepared	**3**
Registration	**18d**	D;E	Provide registration information, or state the study was not registered	**3**
Data sharing	**18e**	D;E	Provide details of the availability of the study data	**13**
Code sharing	**18f**	D;E	Provide details of the availability of the analytical code	**12–13**
**PATIENT & PUBLIC INVOLVEMENT**				
PPI	**19**	D;E	Provide details of patient and public involvement, or state no involvement	**N/A**
**RESULTS**				
Participants	**20a**	D;E	Describe the flow of participants, including those with/without the outcome; a diagram may be helpful	**5, Figure S2**
	**20b**	D;E	Report characteristics overall and by source/setting, including key dates, predictors, treatments, sample size, outcome events, follow-up time, missing data	**5, Table 1**
	**20c**	E	For model evaluation, compare the distribution of important predictors with the development data	**N/A**
Model development	**21**	D;E	Specify the number of participants and outcome events in each analysis	**3–5, 9–10**
Model specification	**22**	D	Provide details of the full prediction model to allow predictions in new individuals and enable third-party evaluation	**7**
Model performance	**23a**	D;E	Report model performance estimates with confidence intervals, including key subgroups; plots may help	**5–10**
	**23b**	D;E	If examined, report heterogeneity in performance across clusters	**N/A**
Model updating	**24**	E	Report results from any model updating, including the updated model and performance	**N/A**
**DISCUSSION**				
Interpretation	**25**	D;E	Give an overall interpretation of the main results, including fairness, in the context of objectives and prior studies	**10**
Limitations	**26**	D;E	Discuss limitations (non-representative sample, sample size, overfitting, missing data) and their effects on bias, uncertainty, generalisability	**11–12**
Usability in current care	**27a**	D	Describe how poor-quality or unavailable input data should be assessed and handled when implementing the model	**11**
	**27b**	D	Specify whether users must interact with input-data handling or model use, and what expertise is required	**11**
	**27c**	D;E	Discuss next steps for future research, with a view to applicability and generalisability	**11–12**

From: Collins GS, Moons KGM, Dhiman P, et al. BMJ 2024;385:e078378. doi:10.1136/bmj-2023-078378
