# Supplementary material for: Alactic Base Excess as a Predictor of Mortality in Trauma ICU Patients: Retrospective Study and Evaluation of Integration into Trauma Scoring Systems
Source: Life (Basel). 2026 Oct 10;16(10):1701. doi: 10.3390/life16101701 (PMC13706226; doi:10.3390/life16101701)
Supplement: Supplementary file 1 [file life-16-01701-s001.zip › Table S1.pdf]

Table S1. Exclusion diagnoses with ICD-10-CM codes, tier classification, and rationale.

Category / Diagnosis	ICD-10-CM Code(s)	Tier	Rationale for exclusion
Chronic Kidney Disease CKD stages 3–5 and ESRD	N18.3, N18.4, N18.5, N18.6	**1**	*CKD causes metabolic acidosis through impaired tubular acid excretion and loss of bicarbonate reabsorption. In stages 3–5 and ESRD, ABE is chronically depressed independent of traumatic physiology, making it impossible to distinguish injury-related metabolic derangement from baseline renal acidosis.*
Chronic Kidney Disease Dependence on renal dialysis	Z99.2	**1**	*Dialysis-dependent patients have absent or minimal residual renal function; their acid-base balance is governed by dialysis scheduling and chronic metabolic acidosis between sessions, entirely unrelated to the traumatic mechanism.*
Diabetic Emergencies DKA — type 1, type 2, malnutrition-related, other/unspecified DM	E10.1, E11.1, E12.1, E13.1, E14.1	**1**	*DKA produces severe high anion-gap metabolic acidosis via accumulation of ketone bodies (beta-hydroxybutyrate, acetoacetate). This profoundly lowers BE and ABE through a mechanism entirely unrelated to haemorrhagic shock or traumatic tissue hypoperfusion.*
Diabetic Emergencies T1DM with hyperglycaemia (without ketoacidosis)	E10.65	**2**	*Hyperglycaemia without overt ketoacidosis does not directly cause metabolic acidosis to the same degree as DKA; severe hyperglycaemia introduces osmotic fluid shifts and can mask/unmask electrolyte abnormalities affecting acid-base interpretation. Excluded for consistency with the broader DKA group — ICD-10 coding does not permit reliable distinction from partial/early DKA.*
Diabetic Emergencies T2DM / other DM with hyperosmolarity (HHS)	E11.0, E13.0	**2**	*HHS is characterised by extreme hyperglycaemia and hyperosmolarity without significant ketoacidosis, not producing metabolic acidosis to the same degree as DKA. Excluded because HHS may co-exist with mild acidosis and ICD-10 coding cannot reliably distinguish pure HHS from mixed DKA/HHS presentations.*
Endocrine / Electrolyte Hypoparathyroidism	E20.x	**1**	*Causes chronic hypocalcaemia and can produce metabolic acidosis through impaired renal tubular acid excretion, directly lowering BE and ABE independent of traumatic physiology.*
Endocrine / Electrolyte Hyperparathyroidism and other parathyroid disorders	E21.x	**1**	*Primary hyperparathyroidism causes hypercalcaemia and affects renal bicarbonate handling, producing mild hyperchloraemic metabolic acidosis or metabolic alkalosis that directly confounds ABE.*
Endocrine / Electrolyte Cushing's syndrome, all types (incl. E24.4 alcohol-induced pseudo-Cushing's)	E24.x	**1**	*Cortisol excess produces mineralocorticoid-like effects (hypokalaemia, sodium retention) and metabolic alkalosis, directly elevating BE and ABE. Alcohol-induced pseudo-Cushing's carries the additional confound of ethanol metabolism producing acetate and lactate.*
Endocrine / Electrolyte Hyperaldosteronism (Conn's and secondary)	E26.x	**1**	*Aldosterone excess drives excessive distal tubular sodium–potassium exchange, causing chronic hypokalaemia and metabolic alkalosis with elevated bicarbonate, directly confounding BE and ABE interpretation.*
Endocrine / Electrolyte Other disorders of adrenal gland (Addison's, adrenocortical insufficiency, adrenal crisis)	E27.x	**1**	*Adrenocortical insufficiency causes type 4 renal tubular acidosis via aldosterone deficiency, producing hyperkalaemia and metabolic acidosis that directly lowers ABE independent of traumatic injury.*
Endocrine / Electrolyte SIADH	E22.2	**3**	*SIADH primarily causes euvolaemic hyponatraemia through inappropriate water retention and does not directly cause metabolic acidosis. Excluded because severe hyponatraemia alters intracellular electrolyte balance and can indirectly affect acid-base interpretation via cellular ion exchange.*
Endocrine / Electrolyte Hyperosmolality and hypernatraemia	E87.0	**3**	*Hypernatraemia itself does not directly cause metabolic acidosis through the same mechanisms as renal or hepatic disease. Excluded because clinically significant hyperosmolality from non-traumatic causes may reflect co-existing severe metabolic disturbance that independently alters acid-base homeostasis.*
Endocrine / Electrolyte Disorders of calcium metabolism (hyper-/hypocalcaemia)	E83.5x	**3**	*Calcium disorders do not directly cause metabolic acidosis through the same mechanisms as renal or hepatic disease. Excluded because the heterogeneity of this code group (malignancy-related, pancreatitis-related, etc.) warranted conservative exclusion.*
Hepatic Disease Acute hepatic failure; hepatic failure, unspecified	K72.0, K72.9	**1**	*The liver is the principal site of lactate clearance via the Cori cycle and gluconeogenesis. Hepatic failure impairs lactate metabolism, directly raising serum lactate and thereby altering the ABE calculation (ABE = BE + lactate).*
Hepatic Disease Chronic hepatitis, all types	K73.x	**2**	*Mild-to-moderate chronic hepatitis may not significantly impair lactate clearance, which requires loss of >80% of functional hepatocyte mass. Excluded conservatively because any degree of chronic hepatic inflammation introduces uncertainty about lactate handling capacity, and ICD-10 coding does not permit assessment of severity.*
Hepatic Disease Other and unspecified cirrhosis of liver	K74.6	**1**	*Established cirrhosis causes impaired lactate clearance (reduced functional hepatocyte mass), portal hypertension, and secondary hyperaldosteronism with metabolic alkalosis — all directly confounding ABE interpretation.*

Category / Diagnosis	ICD-10-CM Code(s)	Tier	Rationale for exclusion
Hepatic Disease Other specified diseases of liver (heterogeneous category)	K76.89	**2**	*This ICD-10 category encompasses a wide range of hepatic pathologies of variable severity and aetiology. Excluded conservatively because the specific conditions coded cannot be reliably characterised by code alone, and any significant hepatic pathology may impair lactate metabolism.*
Toxic / Poisoning Poisoning by insulin and oral hypoglycaemic drugs	T38.3x	**1**	*Insulin overdose causes severe hypoglycaemia-induced lactic acidosis and cellular energy failure, directly lowering ABE through a mechanism unrelated to haemorrhagic shock.*
Toxic / Poisoning Poisoning by aspirin (salicylate toxicity), accidental	T39.011x	**1**	*Salicylate toxicity causes a characteristic mixed high anion-gap metabolic acidosis and respiratory alkalosis via CNS stimulation and uncoupling of mitochondrial oxidative phosphorylation, dramatically altering BE and ABE.*
Toxic / Poisoning Toxic effects of methanol	T51.1x	**1**	*Methanol is metabolised to formic acid, causing a severe high anion-gap metabolic acidosis with profoundly depressed ABE values entirely unrelated to traumatic tissue hypoperfusion.*
Toxic / Poisoning Toxic effects of other organic solvents	T52.8x	**1**	*Organic solvents cause metabolic acidosis through hepatotoxicity, nephrotoxicity, and metabolic conversion to acidic byproducts, confounding ABE through non-traumatic mechanisms.*
Toxic / Poisoning Toxic effects of hydrogen cyanide	T57.3x	**1**	*Cyanide inhibits cytochrome c oxidase (Complex IV), causing histotoxic hypoxia and severe lactic acidosis with very low ABE values driven by cellular respiration inhibition — a mechanism distinct from haemorrhagic shock.*
Toxic / Poisoning Toxic effects of carbon monoxide (motor vehicle exhaust)	T58.01x	**1**	*Carbon monoxide causes tissue hypoxia via carboxyhaemoglobin formation, producing lactic acidosis and reduced ABE. Although CO poisoning frequently co-occurs with trauma (MVA), the toxicological mechanism is distinct from pure haemorrhagic shock.*
Respiratory COPD, all stages	J44.0, J44.1, J44.9	**2**	*CO_2 retention in COPD leads to compensatory renal bicarbonate retention, elevating BE and ABE independent of traumatic metabolism. Excluded because even mild COPD with subclinical CO_2 retention causes compensatory metabolic alkalosis, and severity cannot be reliably distinguished from ICD-10 codes alone.*
Respiratory Chronic respiratory failure with hypoxia	J96.11	**1**	*Chronic respiratory failure causes sustained CO_2 retention and compensatory renal metabolic alkalosis (chronic bicarbonate retention), directly elevating BE and ABE independent of traumatic physiology.*
Other Septic shock	R57.2	**1**	*Septic shock causes profound lactic acidosis through tissue hypoperfusion, cytokine-mediated mitochondrial dysfunction, and impaired lactate clearance — a well-established, direct mechanism. Retained as Tier 1 on mechanism grounds; the practical concern is disentangling concurrent sepsis from trauma in patients with both (consistent with the dedicated sensitivity analysis, n=126 excluding A40/A41, R6520/R6521, which confirmed sepsis coding did not materially alter primary findings).*
Other Dependence on respirator/ventilator (pre-existing)	Z99.11	**2**	*Pre-existing home ventilator dependence (e.g., ALS, SMA, high cervical cord injury) indicates severe underlying disease with chronic respiratory failure and potential compensatory metabolic alkalosis, in the same manner as J96.11 but with additional heterogeneity of underlying cause.*
Other Disorders of branched-chain amino acid and fatty acid metabolism (organic acidaemias)	E71.1x, E71.2x, E71.3x	**1**	*Inborn errors of organic acid and fatty acid metabolism cause intrinsic accumulation of organic acids (propionic acid, methylmalonic acid, isovaleric acid), producing severe chronic metabolic acidosis with very low ABE values entirely unrelated to acute traumatic injury.*
Other Mitochondrial myopathy (incl. MELAS, MERRF, Leigh syndrome)	G71.3	**1**	*Mitochondrial respiratory chain disorders cause chronic lactic acidosis through impaired oxidative phosphorylation, producing persistently depressed ABE values independent of any acute physiological stress from trauma.*
Other Pregnant state, incidental	Z33.1	**2**	*Pregnancy causes physiological respiratory alkalosis with compensatory mild metabolic acidosis, shifting the reference range for BE and ABE. Standard trauma scoring systems (ISS, NISS, TRISS) were not developed or validated for pregnant patients, and haemodynamic responses to haemorrhage differ substantially in pregnancy.*
Other All obstetric and obstetric-related conditions	O00–O9A	**2**	*All obstetric conditions compound the physiological alterations of pregnancy described above. ABE reference ranges and TRISS scoring coefficients used in this study were derived from non-pregnant adult populations and are not applicable to obstetric patients.*

Tier 1: direct and well-established effect on lactate and/or base excess. Tier 2: plausible but indirect, variable or usually modest effect; excluded conservatively. Tier 3: no established direct effect on lactate or base excess; excluded to limit physiological heterogeneity. In MIMIC-IV, ICD-10 codes are assigned at discharge and do not indicate whether a condition was present on admission.
