# Supplementary material for: Alactic Base Excess as a Predictor of Mortality in Trauma ICU Patients: Retrospective Study and Evaluation of Integration into Trauma Scoring Systems
Source: Life (Basel). 2026 Oct 10;16(10):1701. doi: 10.3390/life16101701 (PMC13706226; doi:10.3390/life16101701)
Supplement: Supplementary file 1 [file life-16-01701-s001.zip › TableS2.pdf]

Characteristic	Excluded (missing early ABG)	Final Cohort
Admissions, n	15,987	134
Unique patients, n	13,788	134
Median age, years	64	47
Male sex, %	56.5	75.4
In-hospital mortality, %	11.9	29.1
